# Supplementary material for: Pleistocene sediment DNA reveals hominin and faunal turnovers at Denisova Cave
Source: Nature. 2021 Jun 23;595(7867):399–403. doi: 10.1038/s41586-021-03675-0 (PMC8277575; doi:10.1038/s41586-021-03675-0)
Supplement: Supplementary file 1 — This file contains a Supplementary Discussion, Supplementary References, Supplementary Tables 1-10 and Supplementary Figures 1-44. [file 41586_2021_3675_MOESM1_ESM.pdf]

nature portfolio

https://doi.org/10.1038/s41586-021-03675-0

Supplementary information

Pleistocene sediment DNA reveals hominin and faunal turnovers at Denisova Cave

In the format provided by the authors and unedited

Supplementary Information

SUPPLEMENTARY SECTION 1: COLLECTION OF SEDIMENT SAMPLES FOR ANCIENT DNA ANALYSIS

Supplementary Discussion……… 5

Supplementary Figure 1| Stratigraphic sequence of southeast profile in Main Chamber …………………………. 6

Supplementary Figure 2| Stratigraphic sequences of East Chamber……………………………………………………….. 7

Supplementary Figure 3| Stratigraphic sequences of South Chamber…………………………………………………… 8

SUPPLEMENTARY SECTION 2: STRATIGRAPHY, CHRONOLOGY, ARCHAEOLOGICAL AND ENVIRONMENTAL CONTEXTS

Supplementary Discussion……… 9

SUPPLEMENTARY SECTION 3: ANCIENT DNA PRESERVATION IN SEDIMENTS

Supplementary Discussion……… 12

Supplementary Table 1| Results of Spearman's correlation test evaluating different factors potential impact on DNA preservation in sediment…………………………………………………………………………………………………… 16

Supplementary Figure 4| Measured pH values and the number of ancient mammalian mtDNA fragments recovered ……… 17

Supplementary Figure 5| Library preparation efficiency as a function of sediment samples' pH……………… 17

Supplementary Table 2| Summary of sediment characteristics …………………………………………………………… 18

Supplementary Figure 6| Inferred 5' deamination rates of ancient mtDNA fragments assigned to Bovidae, Canidae, Hyaenidae and Ursidae stratified by extreme phosphatization…………………………………………… 19

Supplementary Figure 7| Average fragment size, number of ancient mtDNA fragments and library preparation efficiency stratified by the presence of extreme phosphatization…………………………………… 20

Supplementary Table 3| p-values from two-sided Wilcoxon test comparing inferred 5' deamination rates for different clast sizes for Canidae, Bovidae, Hyaenidae, and Ursidae……………………………………………21

Supplementary Table 4| p-values from two-sided Wilcoxon test comparing average DNA fragments size, ancient sequences recovered and inferred library preparation efficiency for different clast sizes……………. 22

Supplementary Figure 8| Inferred 5' deamination rates of ancient sequences assigned to Bovidae, Canidae, Hyaenidae and Ursidae across layers stratified by the clast size of sediments……………………………. 22

Supplementary Figure 9| Average fragments size, number of ancient mtDNA fragments recovered and library preparation efficiency in samples stratified by the clast size of sediments……………………………. 23

Supplementary Table 5| p-values from two-sided Wilcoxon test comparing inferred 5' deamination rates for different sediment colours for Canidae, Bovidae, Hyaenidae, and Ursidae…………………………………. 24

Supplementary Table 6| p-values from two-sided Wilcoxon test comparing average DNA fragments size, ancient sequences recovered and inferred library preparation efficiency for different sediment colours…. 25

Supplementary Figure 10| Inferred 5' deamination rates of ancient sequences assigned to Bovidae, Canidae, Hyaenidae and Ursidae across the layers stratified by the colour of sediments………………………….. 26

Supplementary Figure 11| Average fragments size, number of ancient mtDNA fragments recovered and library preparation efficiency in samples stratified by the colour of sediments……………………………………… 27

SUPPLEMENTARY SECTION 4: IDENTIFICATION OF HUMAN MITOCHONDRIAL LINEAGES

Supplementary Discussion…… 28

Supplementary Figure 12| Number of diagnostic positions determined for each branch in the hominin mtDNA tree for four sets of diagnostic positions ………………………………………………………………………. 30

Supplementary Figure 13| Observed support for different hominin mitochondrial lineages using four different sets of diagnostic positions from an unpublished early modern human bone…………………........... 31

Supplementary Figure 14| Observed support for different hominin mitochondrial lineages using four different sets of diagnostic positions from Denisova cave sediment from Slon et al, 2017……………………… 32

Supplementary Figure 15| Histogram of diagnostic position coverage for all deaminated reads from libraries identified as containing ancient hominid DNA…………………………………………………………….. 33

SUPPLEMENTARY SECTION 5: DETERMINING THE NUMBER OF SEQUENCE VARIANTS

Supplementary Discussion…… 34

Supplementary Table 7| Estimated coverage and number of sequence variants present based on a maximum likelihood analysis for libraries with at least 3-fold coverage………………………………………………. 34

SUPPLEMENTARY SECTION 6: RECONSTRUCTING ARCHAIC HUMAN MTDNA CONSENSUS SEQUENCES

Supplementary Discussion…… 35

Supplementary Table 8| Consensus calls for mitochondrial genomes from libraries identified as containing one major contributor……… 35

SUPPLEMENTARY SECTION 7: BUILDING PHYLOGENETIC TREES

Supplementary Discussion…… 36

Supplementary Table 9| The marginal log likelihoods for trees based on the mtDNA protein-coding genes generated from testing different clock and tree models with a path sampling approach………………………… 37

Supplementary Table 10| The estimated tip dates and divergence times (in years) reported from the Tracer program from BEAST2 for the protein-coding region……………………………………………………………….. 37

Supplementary Figure 16| The phylogenetic mtDNA tree determined from Bayesian analysis with Beast2 using protein-coding genes from the mtDNA genome……………………………………………………………… 38

SUPPLEMENTARY SECTION 8: MTDNA HAPLOTYPE IDENTIFICATION FROM SPARSE DATA USING KALLISTO

Supplementary Discussion…… 39

Supplementary Figure 17| mtDNA phylogenetic tree of 95 hominin mtDNA genomes…………………………… 40

Supplementary Figure 18| kallisto abundance estimates for simulated mtDNA with full knowledge of reference genomes…… 41

Supplementary Figure 19| kallisto abundance estimates for simulated mtDNA with simulated genome removed from reference genomes…………………………………………………………………………………………………… 42

Supplementary Figure 20| Example abundance thresholds for 10 mtDNA references……………………………… 43

Supplementary Figure 21| kallisto mtDNA abundances for Denisova Cave sediments…………………………… 44

Supplementary Figure 22| Probabilistic phylogenetic placement of sediment samples…………………………. 45

SUPPLEMENTARY SECTION 9: IDENTIFICATION OF PREVIOUSLY UNKNOWN HOMININ MTDNA LINEAGES

Supplementary Discussion……… 46

Supplementary Figure 23| Histogram of the number of deaminated fragments in sub-samples containing ancient hominin DNA that were not assigned for a lineage…………………………………………………………… 47

Supplementary Figure 24| Observed support for different mtDNA lineages from sample M76………………… 48

Supplementary Figure 25| Time points relative to the estimated tip date of the Altai (Denisova 5) Neanderthal……… 49

Supplementary Figure 26| The log ratio between support for the non-HST (N) Neanderthal branch and N-HST branch for simulated sequences from an ancestralized Altai mtDNA genome to different points back in time…… 50

SUPPLEMENTARY SECTION 10: FAUNAL MITOCHONDRIAL DNA ANALYSIS

Supplementary Discussion……… 51
Supplementary Figure 27| Proportions of ancient mtDNA fragments of large mammals in South Chamber……… 52

SUPPLEMENTARY SECTION 11: FAUNAL IDENTIFICATIONS ON THE SPECIES LEVEL

Supplementary Discussion……… 53

Supplementary Figure 28| The preservation of ancient bear DNA in the stratigraphy of Main Chamber and southeast and northwest faces of East Chamber……………………………………………………………………………… 55

Supplementary Figure 29| The preservation of ancient elephant DNA in the stratigraphy of Main Chamber and southeast and northwest faces of East Chamber…………………………………………………………………………. 56

Supplementary Figure 30| The preservation of ancient hyaena DNA in the stratigraphy of Main Chamber and southeast and northwest faces of East Chamber…………………………………………………………………………. 56

Supplementary Figure 31| Phylogeny of elephant mitochondrial groups used for determining diagnostic positions……… 57

Supplementary Figure 32| Phylogeny of bear mitochondrial groups used for determining diagnostic positions……… 57

Supplementary Figure 33| Phylogeny of hyaena mitochondrial groups used for determining diagnostic positions……… 58

Supplementary Figure 34| Coverage histogram of the woolly mammoth mtDNA genome using mtDNA fragments from all libraries identified as containing ancient elephantid DNA…………………………………………… 58

Supplementary Figure 35| Coverage histogram of the cave bear mtDNA genome using mtDNA fragments from all libraries identified as containing ancient ursid DNA……………………………………………………………… 59

Supplementary Figure 36| Coverage histogram of the spotted hyaena mtDNA genome using mtDNA fragments from all libraries identified as containing ancient hyaenid DNA……………………………………………… 59

Supplementary Figure 37| Coverage at positions in the mtDNA genome that are diagnostic for each elephantid mitochondrial group……………………………………………………………………………………………………… 60

Supplementary Figure 38| Coverage at positions in the mtDNA that are diagnostic for each bear mitochondrial group…… 61

Supplementary Figure 39| Coverage at positions in the mtDNA that are diagnostic for each hyaena mitochondrial group…… 62

Supplementary Figure 40| Identification of different bear mtDNA groups in the stratigraphy of Main Chamber and southeast and northwest faces of East Chamber………………………………………………………………… 63

Supplementary Figure 41| Identification of different elephant mtDNA groups in the stratigraphy of Main Chamber and southeast and northwest faces of East Chamber………………………………………………………………… 64

Supplementary Figure 42| Phylogeny of cave and spotted hyaena mitochondrial haplogroups used for determining diagnostic positions………………………………………………………………………………………………… 64

Supplementary Figure 43| Number of identified diagnostic positions for mtDNA groups in the elephant, hyaena and bear families……………………………………………………………………………………………………… 65

Supplementary Figure 44| Identification of different hyaena mtDNA groups in the stratigraphy of Main Chamber and southeast and northwest faces of East Chamber………………………………………………………………… 66

SUPPLEMENTARY SECTION 1: COLLECTION OF SEDIMENT SAMPLES FOR ANCIENT DNA ANALYSIS

Sediment samples were collected from Pleistocene deposits in all three chambers at Denisova Cave (Extended Data Figure 2a) between the 14th and 22nd of August 2017. Five sediment profiles were sampled:

1) Main Chamber, southeast profile, exposed after excavations in 2016 (Extended Data Figure 2b; Supplementary Figure 1);
2) East Chamber, southeast profile, exposed after excavations in 2015 (Extended Data Figure 2c; Supplementary Figure 2a);
3) East Chamber, northwest profile, exposed after excavations in 2016 (Extended Data Figure 2d; Supplementary Figure 2b);
4) South Chamber, southeast profile (upper), exposed after excavations in 2017 (Supplementary Figure 3a,b);
5) South Chamber, southeast profile (lower), exposed after excavations in 2016 (Supplementary Figure 3c,d).

All profiles exposed in 2015 and 2016 were cleaned back in 2017 immediately prior to sampling. A total of 728 sediment samples were collected from the exposed Pleistocene deposits: 274, 252 and 202 samples in Main, East and South Chambers, respectively (Supplementary Figures 1–3). In each chamber, samples are numbered in the order they were collected, with the prefix ‘M’, ‘E’ and ‘S’ assigned to samples from Main, East and South Chambers, respectively. Sample collection procedures are described in Methods.

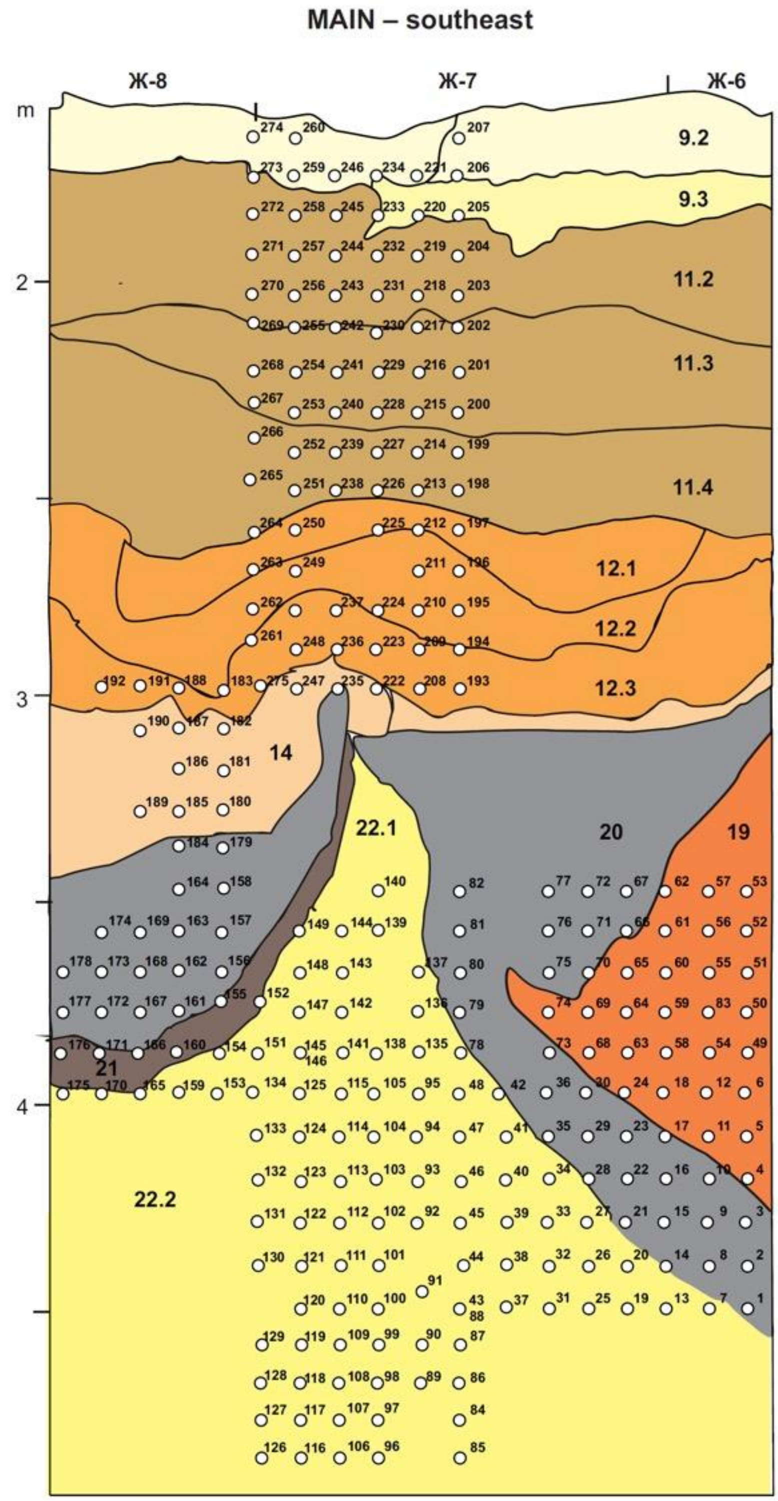

Supplementary Figure 1 | Stratigraphic sequence of southeast profile in Main Chamber after excavation in 2016, indicating locations of sediment samples collected in 2017 after cleaning back this profile. White circles and numbers indicate individual sample locations; all samples from Main Chamber have 'M' preceding the number. Two separate samples were collected from location numbers 43/88 and 145/146.

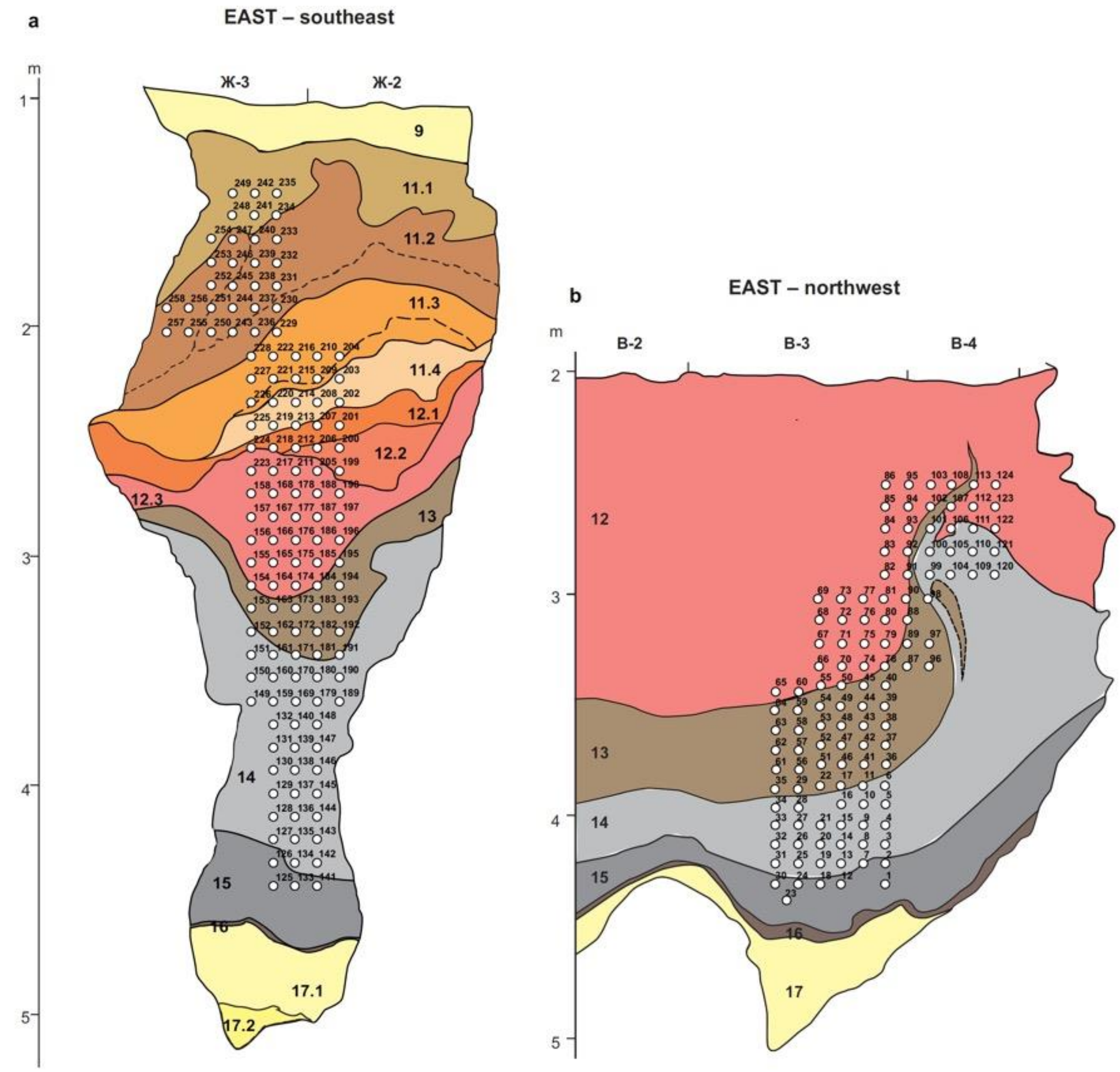

Supplementary Figure 2 | Stratigraphic sequences and locations of sediment samples collected from East Chamber in 2017. White circles and numbers indicate individual sample locations; all samples from East Chamber have 'E' preceding the number. **a**, southeast profile after excavation in 2015. **b**, northwest profile after excavation in 2016. Both profiles were cleaned back in 2017 for sample collection.

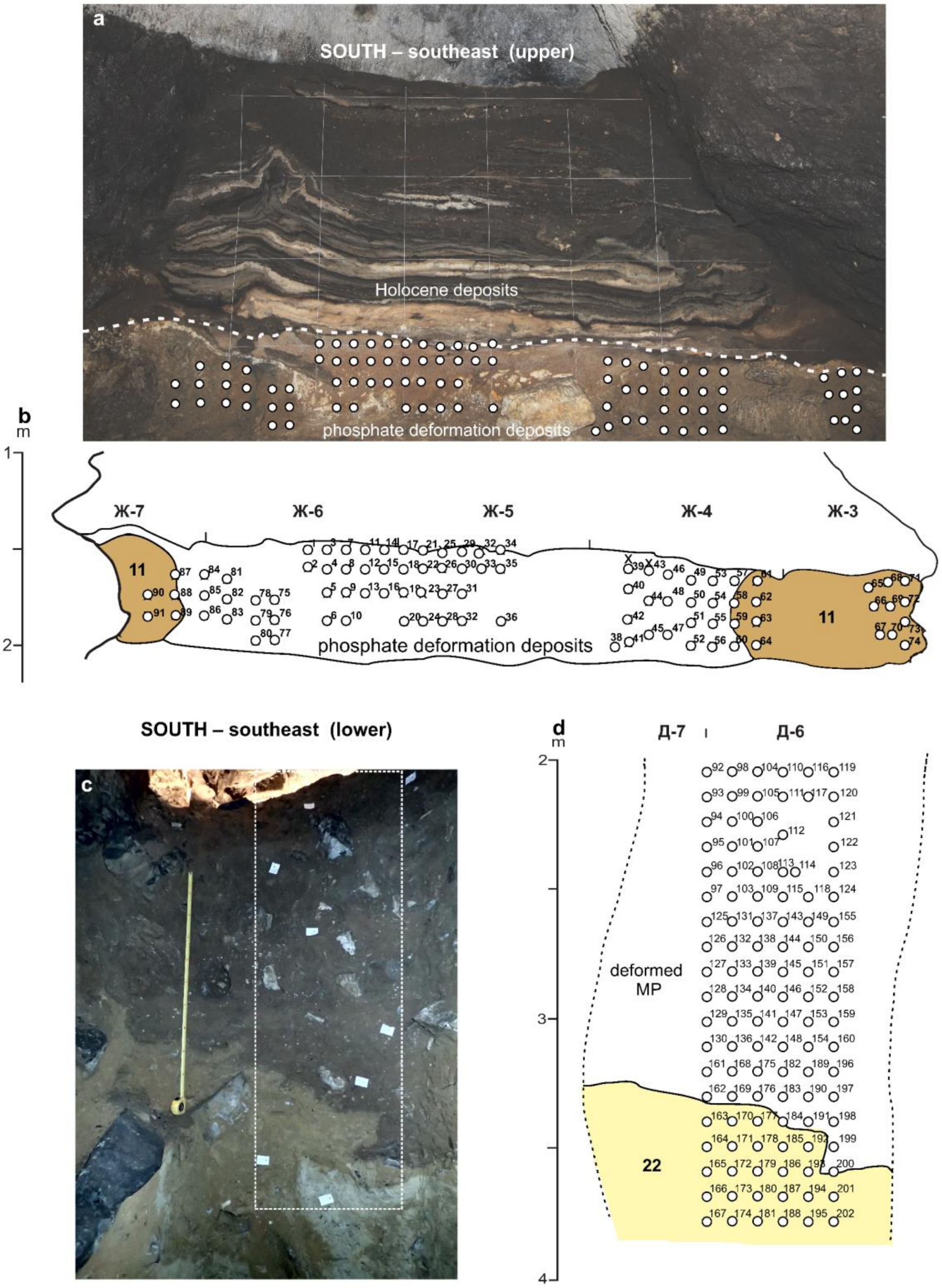

Supplementary Figure 3 | Stratigraphic sequences and locations of sediment samples collected from South Chamber in 2017. White circles and numbers indicate individual sample locations; all samples from South Chamber have 'S' preceding the number. **a**, Photograph of southeast (upper) profile after excavation in 2017 (string lines are 50 cm apart). Dashed line denotes upper boundary of sampled Pleistocene deposits. **b**, Schematic of Pleistocene deposits in panel **a**, showing positions of layer 11 (associated with the IUP) and phosphate deformation deposits (provisionally subdivided into pdd-9 and -12; see Supplementary Section 2 and Supplementary Data File 1). **c**, Photograph of southeast (lower) profile after excavation in 2016; photo taken at an angle and not rectified (scale is 1 m). This profile was cleaned back in 2017 to collect samples from the area delineated by the white box. **d**, Schematic of deposits in panel **c** (MP, Middle Palaeolithic).

SUPPLEMENTARY SECTION 2: STRATIGRAPHY, CHRONOLOGY, ARCHAEOLOGICAL AND ENVIRONMENTAL CONTEXTS

The Pleistocene stratigraphy, chronology, archaeological and environmental contexts for Main and East Chambers are described and discussed in detail in Jacobs et al. (2019)[1] and Douka et al. (2019)[2], together with provisional descriptions of the stratigraphy and sedimentology of the South Chamber profile sampled for optical dating[1]. Further excavations in South Chamber have revealed various stratigraphic complications, however, so analysis and interpretations of its Pleistocene stratigraphy, chronology, archaeology and environmental records are ongoing. We provide below a synopsis for each of the three chambers and highlight additional information of relevance to this study.

2.1 Main Chamber

The Pleistocene deposits in Main Chamber comprise layers 22–9 and their subdivisions. Summary descriptions of the stratigraphy and sedimentology are provided in Jacobs et al. (2019: Supplementary Table 16)[1] and sediment micromorphology observations are provided in Morley et al. (2019)[3] for five samples collected from the southeast and east profiles of the 1984 excavations. Optical dating of 53 sediment samples[1] suggests that the deposits accumulated episodically between the middle of the Middle Pleistocene (start date of 366 ± 43 ka for layer 22.3) and the later part of the Late Pleistocene (end date of 21 ± 8 ka for layer 9.1); here and below, uncertainties on the optical ages are expressed at 95.4% probability.

The southeast profile sampled for sedimentary ancient DNA (aDNA) analysis did not reveal all layers (Supplementary Figure 1); layers 18–15, 13, 11.5 and 11.1 were not exposed at the time of sample collection. A total of 274 individual sediment samples were collected for aDNA analysis, including samples associated with the early Middle Palaeolithic, eMP (layer 22, n = 94; layer 21, n = 6; layer 20, n = 52), the middle Middle Palaeolithic, mMP (layer 19, n = 28; layer 14, n = 11; layer 12, n = 25), the Initial Upper Palaeolithic, IUP (layer 11, n = 47) and the Upper Palaeolithic, UP (layer 9, n = 11). The archaeological associations, environmental contexts and optical ages of these layers are summarised in Jacobs et al. (2019: Figure 3 and Extended Data Table 1)[1].

Key stratigraphic features (Extended Data Figure 2b) include the yellowish, peak-shaped layer 22 near the base of the sequence, the reddish layer 12.3 higher up the sequence, and the distinct, almost horizontal contact between orangey layer 12.1 and greyish layer 11.4 that marks the boundary between the mMP and the IUP, which started 48–45 ka at Denisova Cave[2].

Neanderthal DNA has previously been extracted from three of the sediment samples collected for optical dating from the southeast profile of the 1984 excavations (Jacobs et al., 2019: Extended Data Figure 1a,b)[1]: DCM12-20 (layer 19.1: 129 ± 14 ka), DCM12-18 (layer 17: 138 ± 20 ka) and DCM12-17 (layer 14.3: 98 ± 12 ka)[1,4]. Mitochondrial DNA (mtDNA) was also extracted from a Denisovan molar (*Denisova 2*) recovered from the top of layer 22.1 in 1984[5]. Douka et al. (2019)[2], however, suggest that *Denisova 2* may be intrusive to layer 22.1 and obtained a modelled age of 194–123 ka (95.4% highest posterior density, HPD, interval) for this tooth, which is consistent with the time of deposition of layers 20, 19 and 17.

2.2 East Chamber

The Pleistocene deposits in East Chamber comprise layers 17–9 and their subdivisions. Summary descriptions of the stratigraphy and sedimentology are provided in Jacobs et al. (2019: Supplementary Table 17)[1] and sediment micromorphology descriptions are provided in Morley et al. (2019)[3] for four samples collected from the southeast profile of the 2013 excavations. Optical dating of 37 sediment samples[1] suggests that the deposits accumulated episodically from before 284 ± 32 ka to after 38 ± 9 ka (end dates for deposition of layers 17.1 and 11.1, respectively).

A total of 134 sediment samples for aDNA analysis were collected from the southeast profile exposed after excavation in 2015 (Extended Data Figure 2c; Supplementary Figure 2a). This includes samples associated with the eMP (layer 15, n = 4; layer 14, n = 35), the mMP (layer 13, n = 14; layer 12, n = 33; layer 11.4, n = 8; layer 11.3, n = 11) and the IUP (layer 11.2, n = 22; layer 11.1, n = 7). A further 118 samples were collected from the northwest profile exposed after excavation in 2016 (Supplementary Figure 2b), including samples from layers 15 (n = 8), 14 (n = 33), 13 (n = 36) and 12 (n = 41). Samples were not collected from layers 17, 16 or 9 in either profile. The archaeological associations, environmental contexts and optical ages of these layers are summarised in Jacobs et al. (2019: Figure 3 and Extended Data Table 1)[1].

Key stratigraphic features (Extended Data Figure 2c,d) include the yellowish, culturally sterile layer 17 at the base of the sequence, the blackish colour of layers 15 and 14 that distinguishes them from the overlying, yellowish layer 13, and the distinct charcoal band separating the wedge of pale cream-coloured layer 11.4 from the overlying, reddish layer 11.3. Single-grain equivalent dose (D_e) distributions suggest that deposits associated with layers 17–11.3 are largely intact, with minimal evidence for post-depositional mixing between layers[1]. Post-depositional disturbance (bioturbation) of some parts of layers 11.2, 11.1 and 9 is evident from the macro-stratigraphy and D_e distributions, but undisturbed areas are still present. Sediment samples collected for optical dating from the same profile as that sampled for sedimentary aDNA analysis have D_e distributions consistent with layers 11.4 (DCE16-3), 11.3 (DCE16-2) and 11.1 (DCE16-1) being intact at these locations (Jacobs et al., 2019: Extended Data Figure 3d)[1].

Denisovan DNA was extracted previously from one of the sediment samples collected for optical dating (DCE12-12: 191 ± 26 ka) from layer 15 in the 2012 excavation profile[1,4], providing the earliest evidence for hominin presence in East Chamber. Mitochondrial DNA was also extracted from two Denisovan teeth: *Denisova 8* (from the interface between layers 12 and 11.4) and *Denisova 3* (from layer 11.2). Neanderthal DNA was extracted from two of the sediment samples collected from layer 11.4 for optical dating: DCE14-13 and DCE14-15 (104 ± 12 and 123 ± 22 ka, respectively). The latter sample was erroneously indicated as having originated from layer 14[4] and was later correctly assigned to layer 11.4[1]; it was displayed incorrectly as being from layer 14, however, in Jacobs et al. (2019: Figures 3 and 4)[1] and Douka et al. (2019: Figure 3)[2]. Three Neanderthal bone fragments from East Chamber have also yielded mtDNA: *Denisova 5* (layer 11.4), *Denisova 9* (layer 12.3) and *Denisova 15* (layer 11.4). *Denisova 9* was found in 2011, when layer 12 was not divided into sub-units; its burial depth corresponds most closely to the upper part of layer 12.3 as in Douka et al. (2019)[2], rather than the lower part of layer 12.2 as in Jacobs et al. (2019)[1]. The bone fragment of a Neanderthal–Denisovan offspring (*Denisova 11*) was found in layer 12.3 and has a modelled age of 118–79 ka (95.4% HPD interval)[2].

2.3 South Chamber

Excavations in South Chamber are currently in progress and further work to clarify the stratigraphy and chronology is ongoing. Information about environmental context, including the faunal assemblage, is not available at present and archaeological interpretations are preliminary. The provisional stratigraphic assignments given in Jacobs et al. (2019: Supplementary Table 18)[1] for the profile sampled for optical dating are under revision, and only two of the layers tentatively identified by them—layers 11 and 22, using their numbering system—are considered intact.

Ninety-one sediment samples were collected from the southeast (upper) profile (Supplementary Figure 3a,b). The Pleistocene sediments can be divided into rocky deposits preserved closest to the cave walls, and white-speckled, reddish sediments deposited in between. The rocky deposits have been assigned to layer 11 (Supplementary Figure 3b) and are associated with the IUP; 21 sediment samples were collected from this layer. The intermediate, reddish sediments have been significantly affected by post-depositional phosphatization, and we refer to them as 'phosphate deformation deposits' (pdd) (Supplementary Figure 3a,b). Extensive burrowing near the top of these deposits has also been observed in places. Tentative layer assignments were made in the field, based on associated archaeological finds, and should be considered

provisional: pdd-9 (associated with the UP, n = 13 samples) immediately beneath the Holocene deposits, and pdd-12 (associated with the mMP, n = 57 samples).

A further 111 sediment samples were collected from the southeast (lower) profile (Supplementary Figure 3c,d). These sediments immediately underlie pdd-12 in the upper profile and are associated with mMP assemblages. A distinct stratigraphic boundary is evident between the yellowish deposits of layer 22, from which 25 samples were collected, and the overlying brownish sediments, from which 86 samples were collected. None of these deposits have been visibly impacted by post-depositional phosphatization, but excavations further into these deposits since 2017 have revealed complex post-depositional deformation that prohibits meaningful stratigraphic subdivisions; we refer to these deposits as 'deformed MP' (dMP) (Supplementary Figure 3d).

Owing to uncertainties in the stratigraphic attribution of the pdd (upper profile) and dMP deposits (lower profile), the corresponding samples cannot be interpreted in terms of stratigraphic or chronological patterns in the same way as can be done for Main and East Chambers. Accordingly, the aDNA data from South Chamber are not presented alongside those from Main and East Chambers, but they nonetheless carry valuable information about DNA preservation. None of the aDNA samples from Main and East Chambers were collected from parts of the deposit affected by post-depositional phosphatization, thus providing an opportunity to compare DNA preservation between chambers and between the upper and lower profiles in South Chamber. The aDNA data from South Chamber also provide preliminary insights into the presence or absence of hominin and other mammalian taxa, so a summary of these data is presented in Extended Data Figure 3.

To place the aDNA data for South Chamber in relative stratigraphic and chronological order, we have given tentative layer assignments to samples from the upper profile (pdd-9, layer 11 and pdd-12) and lower profile (dMP and layer 22). The latter samples are assumed to be stratigraphically lower—and therefore older—than those from the upper profile, but some dMP samples may overlap in time with those from pdd-12. The optical ages obtained for layers 22 and 11 indicate the approximate time span of sediment accumulation, with deposition of these layers estimated to have ended 269 ± 97 ka and started after 47 ± 8 ka, respectively[1].

No Neanderthal remains have been found in South Chamber, but a Denisovan tooth (*Denisova 4*) was recovered from layer 11 in 2000. Mitochondrial and nuclear DNA have been extracted from this tooth, which has a modelled age of 84–55 ka (95.4% HPD interval)[2].

SUPPLEMENTARY SECTION 3: ANCIENT DNA PRESERVATION IN SEDIMENTS

The ability to perform high-density sampling of sediment at a single site also enables the examination of potential trends in the preservation and degradation of ancient DNA in relation to both time and different sediment characteristics, while controlling for temperature fluctuation and geographical location. Previous studies relating DNA preservation and sediment are mainly focused on binding properties of DNA to different minerals from laboratory prepared samples[4,6,7]. Studies containing samples collected in the field have been focused on the presence or absence of ancient DNA and do not include in-depth evaluations of DNA preservation and degradation under different conditions or correlations to sediment characteristics[8-10]. Evaluations of DNA degradation from ancient skeletal remains have shown that deamination and fragment size, which are the key parameters frequently used to describe ancient DNA degradation, do not correlate very strongly with sample age in studies involving material from multiple archaeological sites[11,12 13]. Stronger correlations between age and DNA degradation have been observed when analysing material of different ages from a single site[14]. The data set produced in this study thus provides ideal conditions for studying the effect of age, as well as various physical and chemical properties of the samples, on DNA preservation in sediment.

In this section, we use the following four parameters to characterize DNA preservation in sediments from Denisova Cave:

(i) Deamination
The frequency at which cytosines in the reference genome are substituted by thymines (C-to-T substitutions) at the terminal positions of sequence alignments provides a proxy for the level of deamination in a library[15]. The reference in each family that resulted in the most identified fragments was used for each library (Supplementary Data File 1). Initial analyses showed that deamination rates can vary across sequences from different families, even in the same sample, which may be the result of mapping biases (e.g., differences in evolutionary distance between the sequenced fragments and the reference genome), real differences in the properties of DNA molecules, or both. We therefore provide deamination rates separately for the mammalian families that are most abundantly represented throughout the stratigraphy (Bovidae, Canidae, Hyaenidae and Ursidae). We also restricted our analyses to the 5’ ends of sequences, which are less prone to ligation biases in single-stranded library preparation than 3’ ends[16].

(ii) Average fragment length
Average fragment length was computed using all unique mammalian mtDNA sequences from families identified as ancient in each library. Sequences shorter than 35 bp are not used in this calculation, as they were not subjected to taxonomic identification.

(iii) Total number of mammalian mtDNA fragments
This measure reflects the number of unique mammalian mtDNA sequences that were assigned to ancient taxa, normalized by the amount of material (milligrams of sediment) used for DNA extraction. It is important to note that this value is not independent of sequence depth. For some libraries, not enough sequences were generated to ensure the sequencing of all or most unique mtDNA fragments (see Supplementary Data File 1 for duplication rates).

(iv) Inhibition
The co-extraction of inhibitory substances may reduce the efficiency of library preparation, which was determined using the conversion rate of a spiked-in control oligonucleotide [17].

3.1 Dependence of DNA preservation on stratigraphic depth (age)

We tested whether there is a correlation between the relative age of the sediments, as reflected by the layer from which samples were collected, and each of three distinct measures of DNA degradation, using

Spearman's correlation test (data determined not to be normally distributed using a Shapiro-Wilk normality test). First, in order to determine whether deamination rates increase with the relative age of the sediments, we computed the frequency of C-to-T substitutions by layer in the depositional sequence, from top to bottom for each chamber. In all three chambers, and for each ancient mammalian family tested, a significant positive correlation (maximum p-value = 2.7e-08, minimum n = 133) was found between increasing depth in the stratigraphy as represented by layer and deamination rates (Extended Data Figures 3d and 4a, and Supplementary Table 1). Furthermore, we found a significant negative correlation (maximum p-value = 2.0e-25, minimum n = 222) between average fragment size and depth in the stratigraphy for all three chambers (Extended Data Figures 3e and 4b, and Supplementary Table 1), as well as a significant negative correlation (maximum p-value = 0.02, minimum n = 222) between the number of unique ancient mtDNA fragments recovered per milligram of sediment and stratigraphic depth (Extended Data Figures 3f and 4c, and Supplementary Table 1). In summary, significant correlations were found between all parameters of DNA degradation studied here and depth of the sampling locations in the stratigraphy for all three chambers.

3.2. Dependence of DNA preservation on sediment pH

It is known that depurination and other mechanisms contributing to DNA decay proceed faster at low pH[18]. To determine whether the pH of sediment affects the preservation of ancient mammalian DNA at Denisova Cave, the pH was measured for a subset of sediment samples from each layer and chamber. For this purpose, at least 1 g of sediment was aliquoted into a 2 mL tube. The sample was then weighed on a weigh boat and left to air dry at room temperature for about 1.5 hours. The weight after drying was recorded. The dried sediment was then homogenized using a mortar and pestle. Each sediment sample was split in half (based on its weight), transferred into a fresh 2 mL tube and resuspended through vortexing (2–3 minutes) in a volume of water corresponding to approximately twice the sample weight (e.g., 1 ml water for 500 mg sediment). The samples were left to settle for at least 60 minutes and were then centrifuged in a table-top centrifuge (2 minutes at maximum speed). The supernatant was transferred to 5 mL tubes and pH measurements were performed using a Mettler Toledo Seven Easy pH meter. Three measurements were taken for each original sample: one for each half of the sample, and the third after pooling the supernatants from both halves. For some sediment subsamples, the volumes of supernatant remaining after the settling phase were below the specifications of the pH meter, so only the pH values resulting from the pooled supernatants were used for analysis. The associated data can be found in Supplementary Data File 2.

For the vast majority of samples, we obtained pH values in a narrow range between 7.5 and 8 (Supplementary Figure 4). Only four out of 58 samples produced neutral or slightly acidic pH values (between 6 and 7). Of those, only one yielded small amounts of ancient mammalian mtDNA. A significant correlation was found between the amount of DNA recovered and the pH of the sample (Spearman's correlation test: rho value = 0.39, p-value = 0.003, n = 58). When controlling for time, as represented by layer (excluding samples that cover multiple layers), this correlation remains significant (multiple regression analysis: p-value = 0.013, n = 46). We also tested whether the inferred level of inhibition in library preparation correlates with the pH of the samples, to investigate whether low or high pH values may lead to the co-extraction of inhibitory substances, but found no correlation (Supplementary Figure 5) (Spearman's correlation test: rho value = 0.017, p-value = 0.90, n = 57). Taken together, these results indicate that low pH negatively impacts DNA preservation in sediments, in agreement with a previous study on lake sediments[19].

3.3. Dependence of DNA preservation on post-depositional phosphatization

The presence of phosphates can impact the pH of sediments and may interfere with DNA binding to minerals. We tested this by comparing the presence of post-depositional phosphatization in the Pleistocene layers of each chamber (Supplementary Table 2)[1,3,20] to the DNA characteristics described above: the

deamination frequency, average DNA fragment size, number of fragments assigned to ancient taxa per mg of sediment and the inferred inhibition level (Supplementary Figures 6 and 7). Many of the sedimentary layers at Denisova Cave contain localized zones of phosphatization (e.g., phosphatic rinds around limestone clasts[3]), but areas of extensive phosphatization in the Pleistocene deposits are restricted to parts of the uppermost layers in Main Chamber (layer 9)[1], East Chamber (layers 9 and 11.1)[20] and South Chamber (pdd-9 and -12, where 'pdd' denotes phosphate deformation deposits; Extended Data Figure 3a)[1].

There was no significant difference in the amount of ancient DNA recovered or inferred inhibition observed when comparing layers with and without extensive phosphatization when using a Wilcoxon test (data not normally distributed based on a Shapiro-Wilk test) and correcting for multiple testing[21] (amount of ancient DNA: p-value = 0.15, n = 664; inhibition: p-value = 0.7, n = 707, respectively). However, the DNA recovered from these layers had longer average fragment sizes and lower deamination rates (average size: p-value = 3.7E-07, n = 664; 5' deamination: p-value = <2E-16, n = 554 (Canidae), p-value = <2E-16, n = 587 (Bovidae), p-value = <2E-16, n = 473 (Hyaenidae), p-value = 3.5E-15, n = 493 (Ursidae); Wilcoxon test corrected for multiple testing[21]). These trends remain significant when controlling for time, as represented by layer, with a type III anova test (average size: p-value = 3.36E-04, n = 572; 5' deamination: p-value = 8.07E-06, n = 399 (Canidae), p-value = 2.89E-06, n = 410 (Bovidae), p-value = 3.12E-05, n = 362 (Hyaenidae), p-value = 5.1E-12, n = 385 (Ursidae)).The longer fragment sizes may be due to competition between phosphate in the DNA backbone and free phosphate in the sediment for binding to mineral surfaces, or because these are younger layers. The presence of phosphate is linked to the pH values of sediments, as the heavily phosphatized layers produced the lowest measured pH values from all layers across all three chambers (Supplementary Figure 4). Phosphates can acidify neutral or basic soils, so post-depositional phosphatization may be the driving factor behind the observed decrease in pH. However, the Pleistocene layers with the most extensive phosphatization are also located closest to the interface with the overlying Holocene deposits, so the pH of the uppermost Pleistocene sediments may also have been affected by diagenetic changes associated with the microbial degradation of bat guano in the lowermost Holocene deposits[20].

3.4 DNA preservation and clast size

The size range of clastic material comprising each layer could relate to the surface area on sediment particles available for the binding and preservation of DNA. Most of the Pleistocene layers in Denisova Cave are composed of poorly sorted silt and sand grains washed or blown into the cave, or reworked from pre-existing cave sediments, interstratified with variable amounts of angular clasts of spalled limestone (gravel, cobble and boulder in size)[1]. We examined the average DNA fragment size, number of fragments assigned to ancient taxa per mg of sediment, inferred inhibition and deamination for samples collected from various layers in Main and East Chambers (Supplementary Table 2 and Supplementary Figures 8 and 9). DNA was recovered from layers composed predominantly of fine-grained sediments (e.g., layers 9 and 21 in Main Chamber) and from layers containing larger proportions of coarser clasts (e.g., parts of layer 11 in both Main and East Chambers), which indicates that DNA can be preserved in deposits containing clasts of a variety of sizes.

3.5. DNA preservation and sediment colour

The colour of sediment may indicate the presence of different types of organics (e.g., charcoal, bone and coprolite fragments), metals and other substances that could affect DNA preservation. We compared the colour of each layer[1] to the deamination rate, average fragment size, ancient mammalian mtDNA fragments per milligram sediment, and inferred inhibition for samples collected from Main and East Chambers (Supplementary Figures 10 and 11). While no significant trends were found in relation to inferred deamination rates, blackened sediments had significantly shorter and less ancient DNA fragments than other sediment colours, except for rusty ochre or yellow (Supplementary Tables 5 and 6). Blackened sediments showed decreased library preparation efficiency in comparison to all other sediment colours,

except for grey (Supplementary Table 6). It was possible to recover DNA from layers of all colours, but those black or yellow in colour produced the lowest yields. However, each sediment colour was observed in no more than three layers (Supplementary Table 1) and typically deposited over a limited time span. Blackened, yellow and rusty ochre sediments all originate from the lowest layers in Main and East Chambers, so these results are likely driven by the impacts of time. Further data from additional sites are needed, therefore, to ascertain whether the differences observed here are attributable solely to sediment colour or whether they result from uneven sampling of the stratigraphy.

Variables	Family	Chamber	Rho	p-value	n
5' deamination and layer	Canidae	East	0.6	1.75E-22	219
5' deamination and layer	Canidae	Main	0.74	5.19E-40	224
5' deamination and layer	Canidae	South	0.47	1.31E-07	112
5' deamination and layer	Bovidae	East	0.69	2.71E-34	235
5' deamination and layer	Bovidae	Main	0.65	2.01E-25	201
5' deamination and layer	Bovidae	South	0.34	1.80-05	150
5' deamination and layer	Hyaenidae	East	0.73	3.79E-35	202
5' deamination and layer	Hyaenidae	Main	0.46	2.66E-08	133
5' deamination and layer	Hyaenidae	South	0.36	1.80E-05	139
5' deamination and layer	Ursidae	East	0.47	1.12E-11	190
5' deamination and layer	Ursidae	Main	0.76	1.21E-39	204
5' deamination and layer	Ursidae	South	0.41	2.85E-05	100
Average fragment size and layer	NA	East	-0.62	2.02E-25	222
Number of ancient sequences per mg sediment and layer	NA	East	-0.16	1.90E-02	222
Average fragment size and layer	NA	Main	-0.77	4.69E-50	249
Number of ancient sequences per mg sediment and layer	NA	Main	-0.72	6.04E-41	249
Average fragment size and layer	NA	South	-0.58	1.85E-08	79
Number of ancient sequences per mg sediment and layer	NA	South	-0.46	1.66E-05	79

Supplementary Table 1 | The results of Spearman’s correlation test (one-sided), which was performed on various variables in an effort to evaluate different factors potential impact on DNA preservation in sediment. Samples not assigned to a single layer were not included in tests, this includes samples in the dMP section of South Chamber.

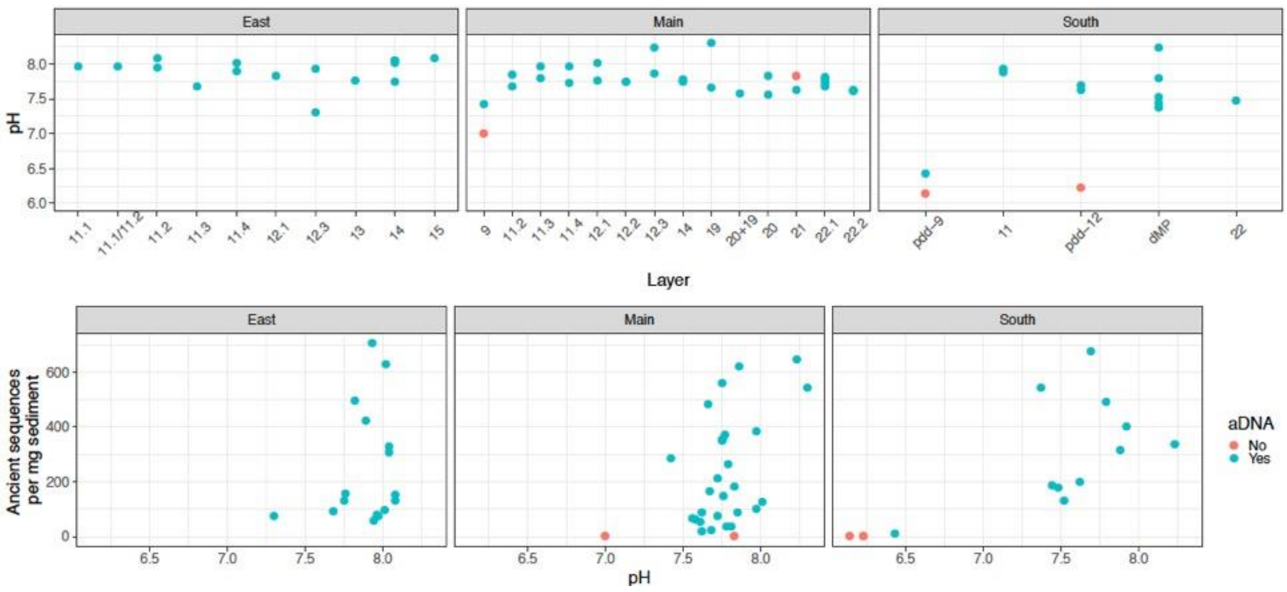

Supplementary Figure 4 | Measured pH values for a sub-set of sediment samples from layers in East, Main and South Chambers (top) and the number of ancient mammalian mtDNA fragments recovered per milligram sediment versus measured pH (bottom). Layers are sorted from top to bottom in the stratigraphy. The dMP (deformed Middle Palaeolithic) layer designation in South Chamber is a section of the stratigraphy where the layer assignments are not finalized, but are part of the Middle Palaeolithic. Layers marked pdd were impacted by post-depositional phosphatization. Each point is coloured depending on if ancient DNA was recovered from the respective sample.

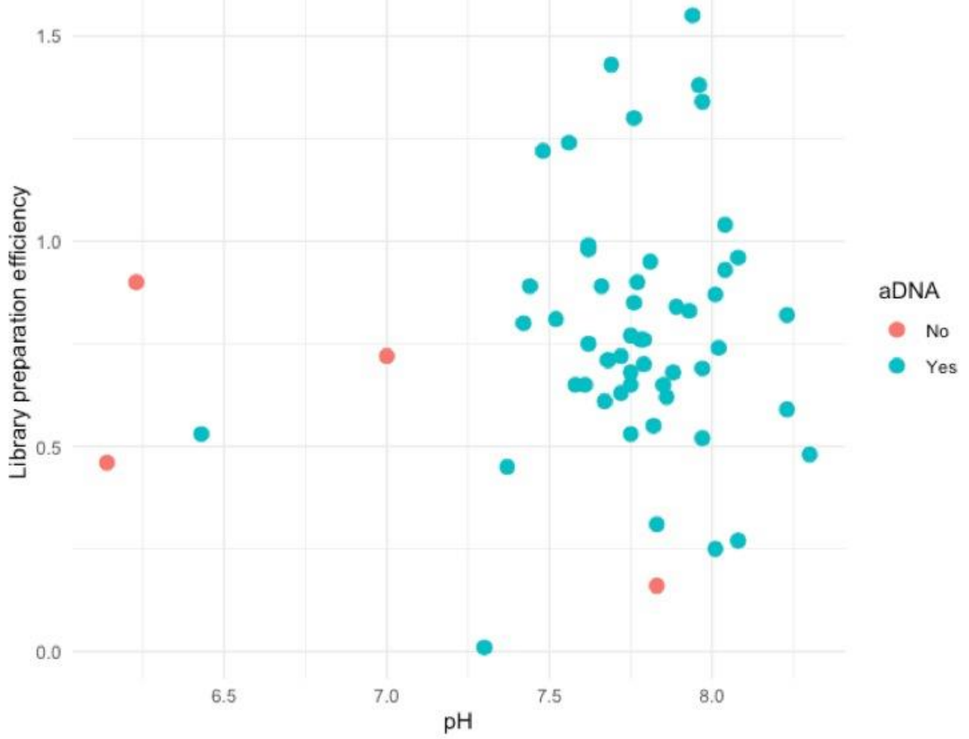

Supplementary Figure 5 | Library preparation efficiency as a function of the pH of sediment samples. Each point represents one sample from Main, East or South Chamber for which pH was determined. Library preparation efficiency was determined by comparing the number of spike-in control molecules recovered in the relevant library to that in the library negative controls. Samples with an efficiency lower than 0.5 are considered to show inhibition. The points are coloured depending on if evidence for ancient DNA preservation was found in the sample. Note that technical variation in qPCR measurements may lead to library preparation efficiencies greater than 1.

Chamber	Layer	Color	Clastic Size	Extensive phosphatization
Main	9	brown	fine	yes
	11.1	grey-redtint	coarse	no
	11.2	grey	-	no
	11.3	grey	-	no
	11.4	greyishbrown	fine	no
	11.5	brown/red	coarse and fine	no
	12.1	greyish brown	coarse and fine	no
	12.2	brown/red	fine	no
	12.3	brown/red	fine	no
	14	-	fine	no
	19	brown/red	-	no
	20	greyish brown		no
	21	black/brown	fine	no
	22.1	rusty ochre	coarse	no
	22.2	yellow	-	no
East	11.1	-	fine	yes
	11.2	brown/red	-	no
	11.3	brown	-	no
	11.4	grey	coarse with boulders	no
	12.1	greyish brown	-	no
	12.2	brown/red	-	no
	12.3	greyish brown	coarse and fine	no
	13	brown	fine	no
	14	brown	-	no
	15	black/brown	coarse and fine	no
South	pdd-9	-	-	yes
	11	-	-	no
	pdd-12	-	-	yes
	dMP	-	-	no
	22	-	-	no

Supplementary Table 2 | Summary of sediment characteristics described in Jacobs et al., 2019 that were used for investigating trends in ancient DNA preservation. Layers with no published information or where sub-units within in the layer had different descriptions were excluded and are represented by “-“.

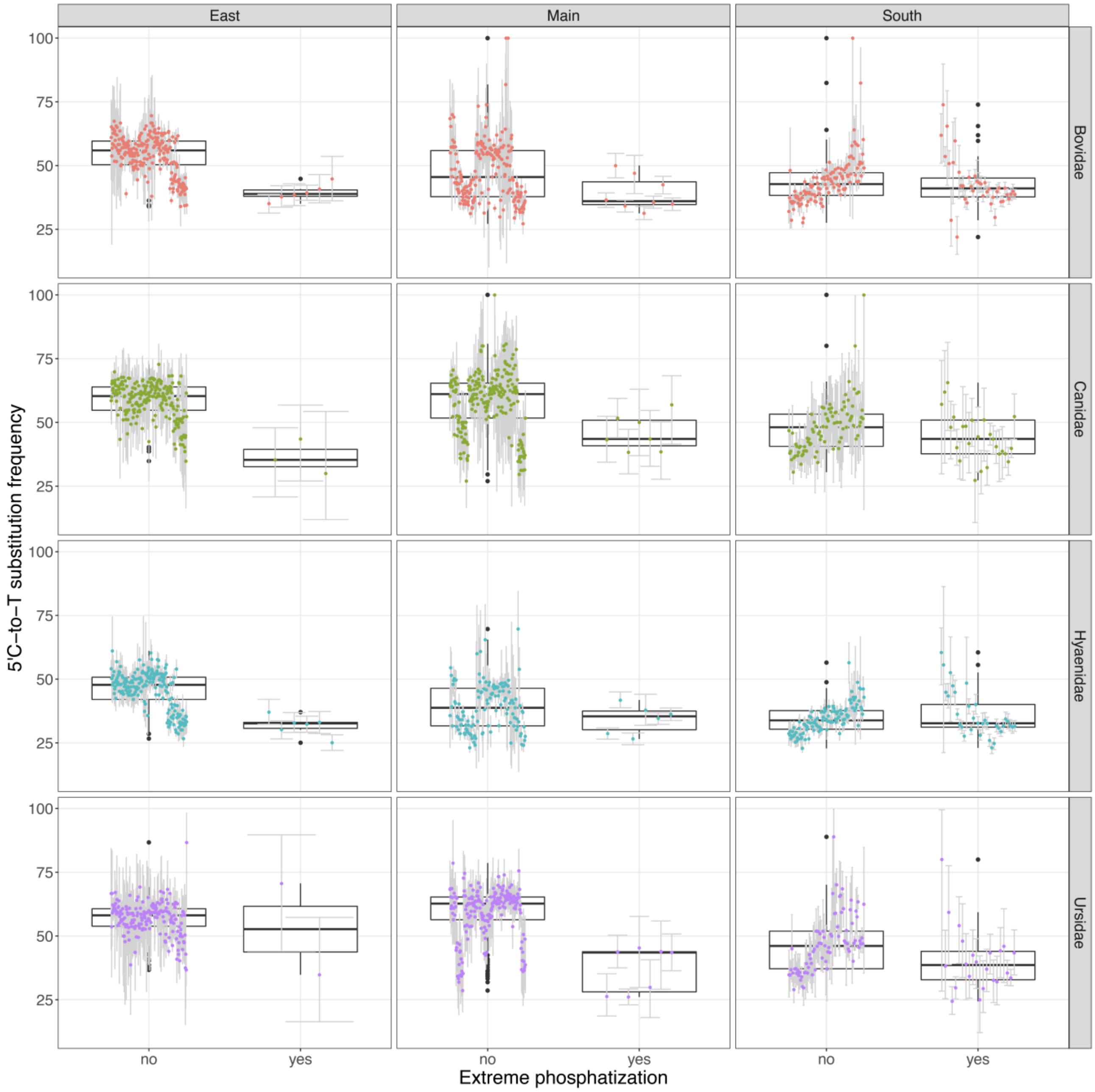

Supplementary Figure 6 | Inferred 5' deamination rates of ancient mtDNA fragments assigned to Bovidae, Canidae, Hyaenidae and Ursidae across the layers in East, Main and South Chambers stratified by extreme phosphatization. Each point (centre of error bar) represents the average observed 5' C to T substitution frequency in a library from a specific layer in the relevant chamber. The grey bars represent the 95% binomial confidence intervals based on the number of alignments starting at a C in the reference genome. The box plots show the distribution of the observed 5' C to T substitution frequencies following the standard Tukey representation (box limits represent the lower and upper quartiles, whiskers represent 1.5 times the interquartile range, outliers are represented by black dots). Wilcoxon test with a correction for multiple testing was used to compare layers with and without extreme phosphatization (p-value = <2E-16, n = 554 (Canidae), p-value = <2E-16, n = 587 (Bovidae), p-value = <2E-16, n = 473 (Hyaenidae), p-value = 3.5E-15, n = 493 (Ursidae)).

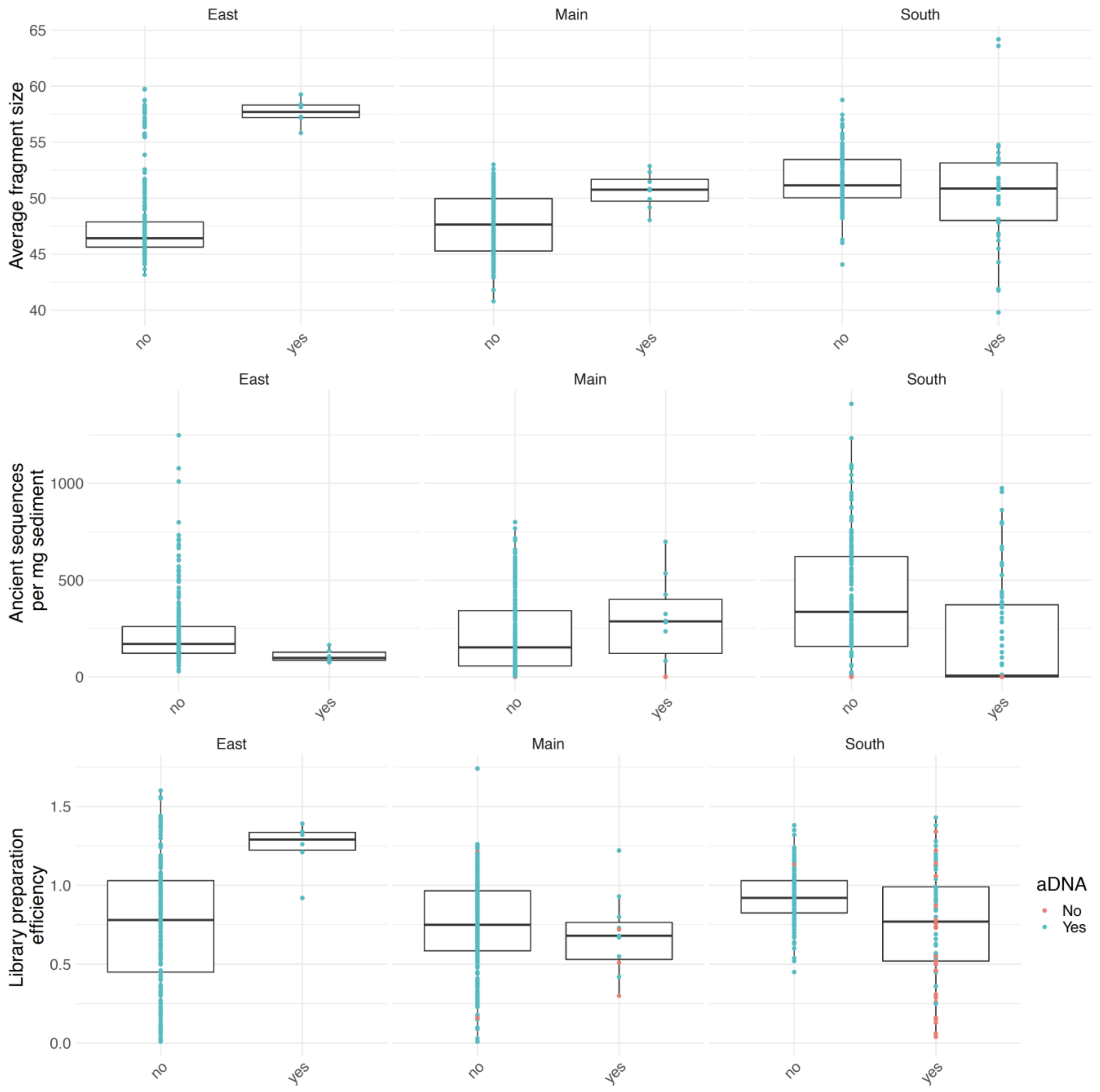

Supplementary Figure 7 | Average fragment size, the number of ancient mtDNA fragments recovered per milligram sediment and library preparation efficiency in samples from East, Main and South Chambers stratified by the presence of extreme phosphatization. Each point represents a single sample and is coloured depending on if that sample was identified as containing ancient DNA. The box plots show the distribution of the observed average fragment lengths, number of fragments assigned to ancient taxa per milligram of sediment, and library preparation efficiency following the standard Tukey representation (box limits represent the lower and upper quartiles, whiskers represent 1.5 times the interquartile range, outliers are represented by black dots). Note that technical variation in qPCR measurements may lead to library preparation efficiencies greater than 1. The difference between layers with and without extreme phosphatization was tested with a Wilcoxon test (corrected for with multiple testing) (average size: p-value = 3.7E-07, n = 664; amount of ancient DNA: p-value = 0.15, n = 664; inhibition: p-value = 0.7, n = 707).

	Course with boulders	Coarse	Coarse and fine
	Canidae (n=442)		
Coarse	4.50E-05		
Coarse and fine	5.20E-04	3.10E-09	
Fine	0.23	6.90E-11	8.50E-03
	Bovidae (n=435)		
Coarse	3.90E-01		
Coarse and fine	1.30E-03	2.01E-01	
Fine	0.841	2.72E-01	4.10E-05
	Hyaenidae (n=334)		
Coarse	4.00E-01		
Coarse and fine	4.30E-05	8.10E-06	
Fine	4.72E-01	8.52E-02	4.00E-04
	Ursidae (n=393)		
Coarse	2.90E-05		
Coarse and fine	9.40E-03	7.60E-16	
Fine	4.36E-01	4.40E-11	1.34E-01

Supplementary Table 3 | p-values from two-sided Wilcoxon test comparing inferred 5' deamination rates for different clast sizes for Canidae, Bovidae, Hyaenidae, and Ursidae. Significant p-values are highlighted in yellow. All p-values have been corrected for multiple testing using the Benjamini and Yekutieli ("BY") method.

	Coarse with boulders	Coarse	Coarse and fine
	Average Size		
Coarse	5.6E-04 (^)		
Coarse and fine	5.6E-04 (<)	8.2E-03 (<)	
Fine	1.1E-01 (^)	3.8E-07 (<)	6.6E-04 (^)
	Ancient sequences per mg		
Coarse	6.5E-05 (^)		
Coarse and fine	2.3E-04 (^)	1.9E-15 (<)	
Fine	6.0E-04 (^)	1.3E-18 (<)	3.5E-02(<)
	Library preparation efficiency		
Coarse	2.6E-03 (<)		
Coarse and fine	6.7E-02 (<)	1.4E-02 (^)	
Fine	2.1E-01 (<)	3.3E-03 (^)	7.9E-01 (<)

Supplementary Table 4 | p-values from two-sided Wilcoxon test comparing average DNA fragments size, ancient sequences recovered and inferred library preparation efficiency for different clast sizes. Significant p-values are highlighted in yellow. All p-values have been corrected for multiple testing using the Benjamini and Yekutieli ("BY") method. The clastic size with the longer average size, higher number of ancient sequences recovered or more efficient library preparation is pointed to (<).

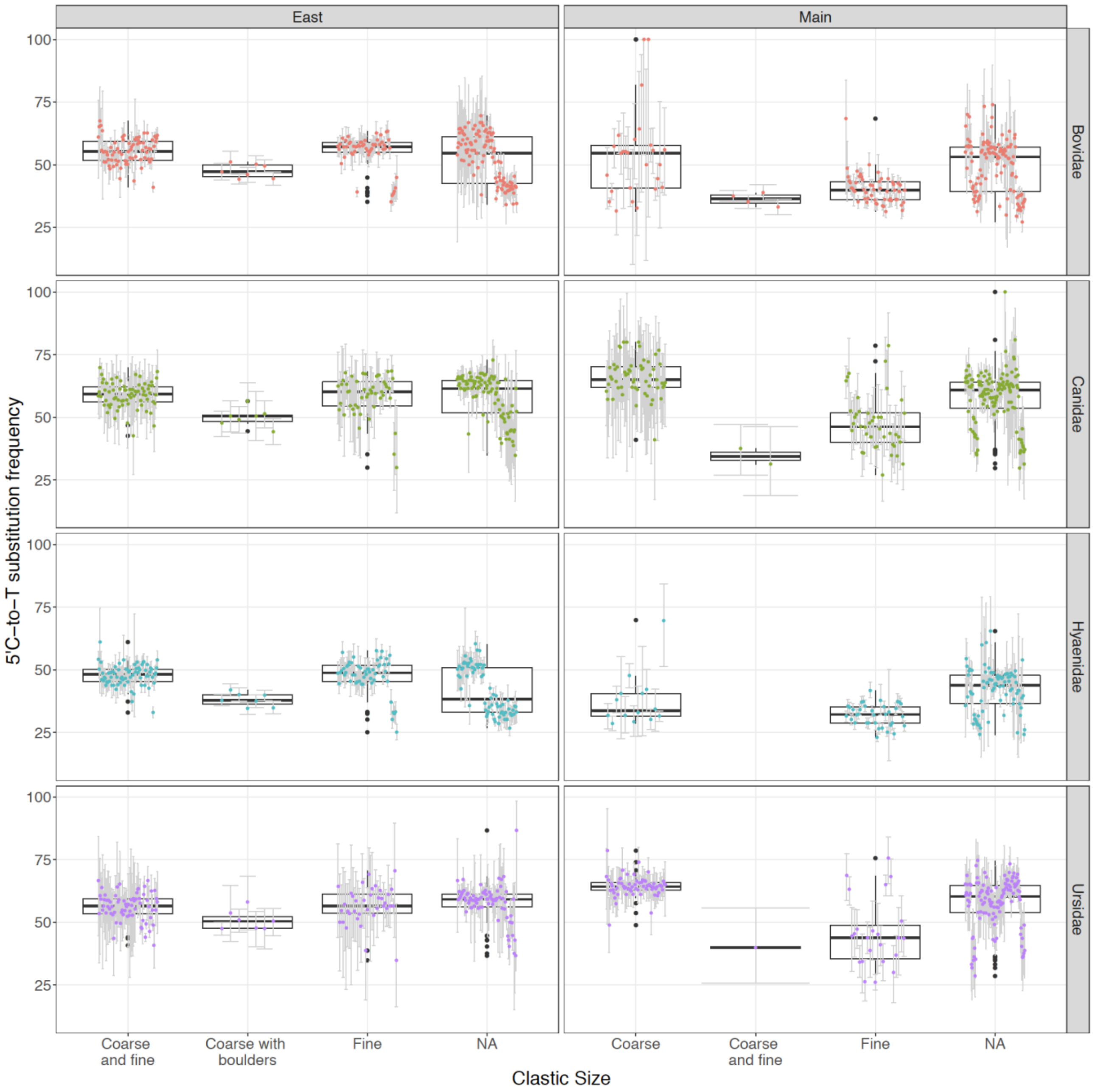

Supplementary Figure 8 | Inferred 5' deamination rates of ancient sequences assigned to Bovidae (n=435), Canidae (n=442), Hyaenidae (n=334) and Ursidae (n=393) across the layers in East and Main Chambers stratified by the clast size of sediments. Each point (centre of error bar) represents the average observed 5' C to T substitution frequency in a library from a specific layer in the relevant chamber. The grey bars represent the 95% binomial confidence intervals based on the number of alignments starting at a C in the reference genome. The box plots show the distribution of the observed 5' C to T substitution frequencies following the standard Tukey representation (box limits represent the lower and upper quartiles, whiskers represent 1.5 times the interquartile range, outliers are represented by black dots). Layers with no published information or where sub-units within in the layer had different descriptions were excluded and are represented by "NA".

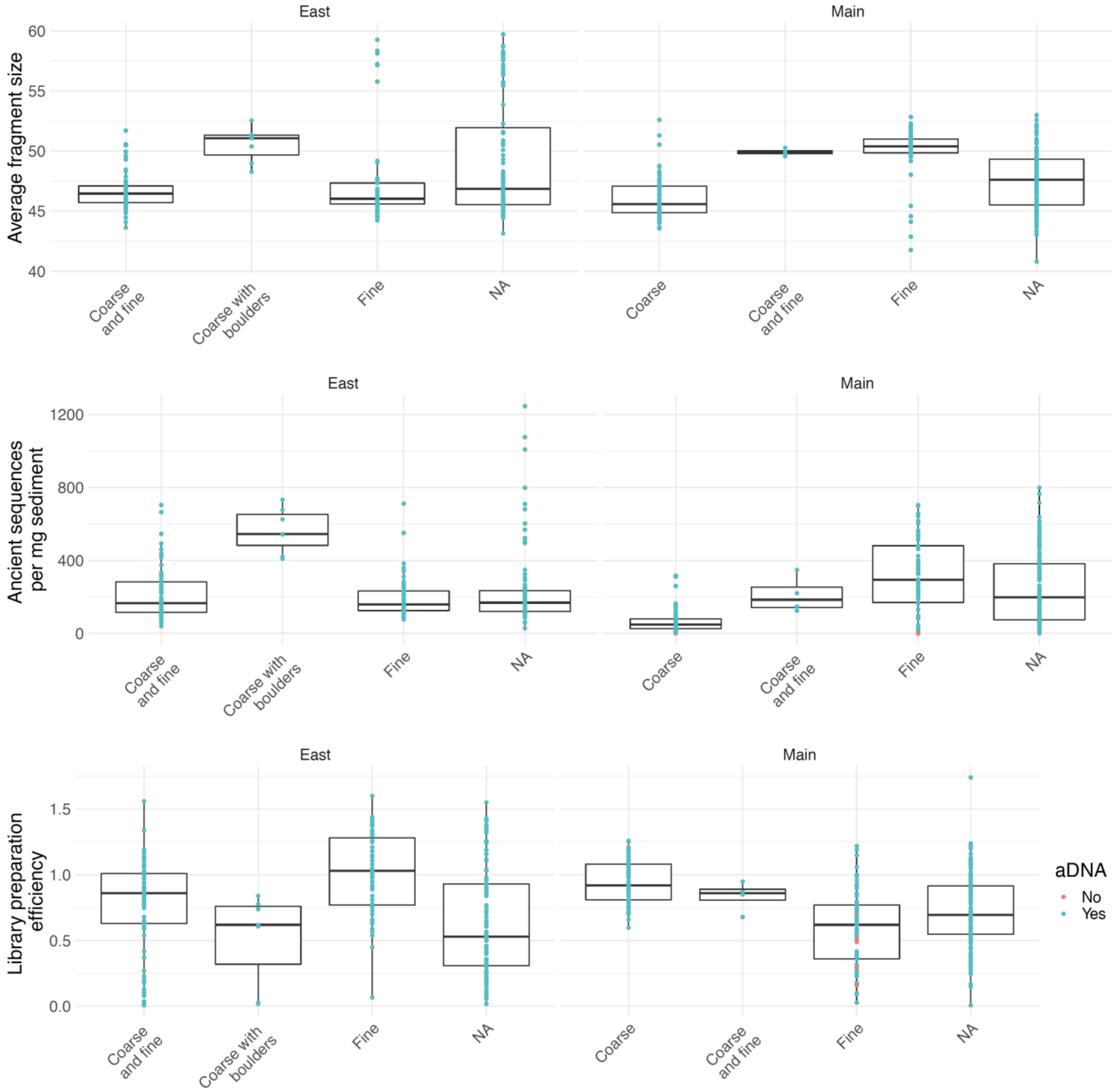

Supplementary Figure 9 | Average fragments size, the number of ancient mtDNA fragments recovered per milligram sediment and library preparation efficiency in samples from East and Main Chambers stratified by the clast size of sediments. The different clastic sizes were defined as coarse and fine (n=76), coarse with boulders (n=7), coarse (n=64), and fine (n=109). Each point represents a single sample and is coloured depending on if that sample was identified as containing ancient DNA. The box plots show the distribution of the observed average fragment lengths, number of fragments assigned to ancient taxa per mg of sediment, and library preparation efficiency following the standard Tukey representation (box limits represent the lower and upper quartiles, whiskers represent 1.5 times the interquartile range, outliers are represented by black dots). The “NA” assignment indicates that there was no published information for this layer (n=408). Note that technical variation in qPCR measurements may lead to library preparation efficiencies greater than 1.

	Black/brown	Brown	Brown/red	Grey	Greyish brown	Rusty ochre
	Canidae (n=442)					
Brown	6.25E-02					
Brown/red	7.20E-07	2.70E-09				
Grey	3.10E-08	1.10E-14	4.80E-07			
Greyish brown	1.01E-02	9.29E-02	5.10E-07	8.80E-13		
Rusty ochre	2.88E-01	2.10E-06	5.40E-14	5.80E-14	1.20E-08	
Yellow	9.36E-01	3.59E-02	6.00E-07	1.10E-09	6.10E-03	3.19E-01
	Bovidae (n=435)					
Brown	6.89E-02					
Brown/red	9.70E-06	9.60E-16				
Grey	7.80E-07	< 2e-16	2.20E-07			
Greyish brown	7.81E-03	4.60E-04	7.70E-08	3.20E-13		
Rusty ochre	3.53E-02	2.30E-02	2.69E-02	1.50E-05	5.15E-01	
Yellow	1.38E-02	2.66E-02	7.77E-02	6.50E-05	3.12E-01	8.88E-01
	Hyaenidae (n=334)					
Brown	4.89E-01					
Brown/red	1.00E-04	5.80E-14				
Grey	4.50E-06	8.50E-10	5.00E-04			
Greyish brown	7.52E-02	8.24E-03	5.30E-12	1.10E-09		
Rusty ochre	3.07E-03	8.40E-05	3.69E-01	3.28E-02	1.90E-04	
Yellow	6.70E-03	3.10E-04	7.50E-01	5.98E-02	3.70E-04	7.07E-01
	Ursidae (n=393)					
Brown	1.42E-02					
Brown/red	2.30E-04	5.80E-04				
Grey	2.60E-07	9.40E-11	4.60E-05			
Greyish brown	7.26E-03	4.35E-01	4.02E-03	3.40E-10		
Rusty ochre	7.10E-02	2.50E-15	7.20E-11	1.20E-12	1.90E-14	
Yellow	1.66E-02	6.80E-12	2.40E-09	5.30E-10	4.10E-11	5.42E-02

Supplementary Table 5 | p-values from two-sided Wilcoxon test comparing inferred 5' deamination rates for different sediment colours for Canidae, Bovidae, Hyaenidae, and Ursidae. Significant p-values are highlighted in yellow. All p-values have been corrected for multiple testing using the Benjamini and Yekutieli ("BY") method.

	Black/brown	Brown	Brown/red	Grey	Greyish brown	Rusty ochre
	Average Size					
Brown	1.90E-02					
Brown/red	4.10E-09	6.60E-20				
Grey	6.00E-08	7.10E-14	1.00E+00			
Greyish brown	1.10E-03	7.10E-02	4.90E-17	4.20E-13		
Rusty ochre	7.10E-01	9.00E-02	2.30E-17	6.80E-14	1.40E-03	
Yellow	1.00E+00	5.60E-03	6.10E-13	3.60E-11	3.70E-04	6.80E-01
	Ancient sequences per mg					
Brown	5.40E-05					
Brown/red	1.00E-05	3.30E-04				
Grey	6.60E-08	1.50E-05	1.00E+00			
Greyish brown	5.70E-04	1.00E+00	1.20E-04	2.60E-05		
Rusty ochre	3.80E-01	3.70E-20	1.80E-17	3.00E-14	8.40E-17	
Yellow	7.30E-01	1.00E-10	1.70E-10	7.70E-13	1.10E-08	1.00E+00
	Library preparation efficiency					
Brown	4.60E-02					
Brown/red	7.20E-03	3.30E-01				
Grey	1.70E-01	1.00E+00	8.90E-01			
Greyish brown	2.50E-03	1.70E-01	1.00E+00	2.50E-01		
Rusty ochre	7.90E-06	3.40E-07	8.60E-03	9.50E-11	3.40E-04	
Yellow	3.20E-04	3.90E-03	2.50E-01	7.50E-06	2.20E-01	7.80E-01

Supplementary Table 6 | p-values from two-sided Wilcoxon test comparing average DNA fragments size, ancient sequences recovered and inferred library preparation efficiency for different sediment colours. Significant p-values are highlighted in yellow. All p-values have been corrected for multiple testing using the Benjamini and Yekutieli (“BY”) method.

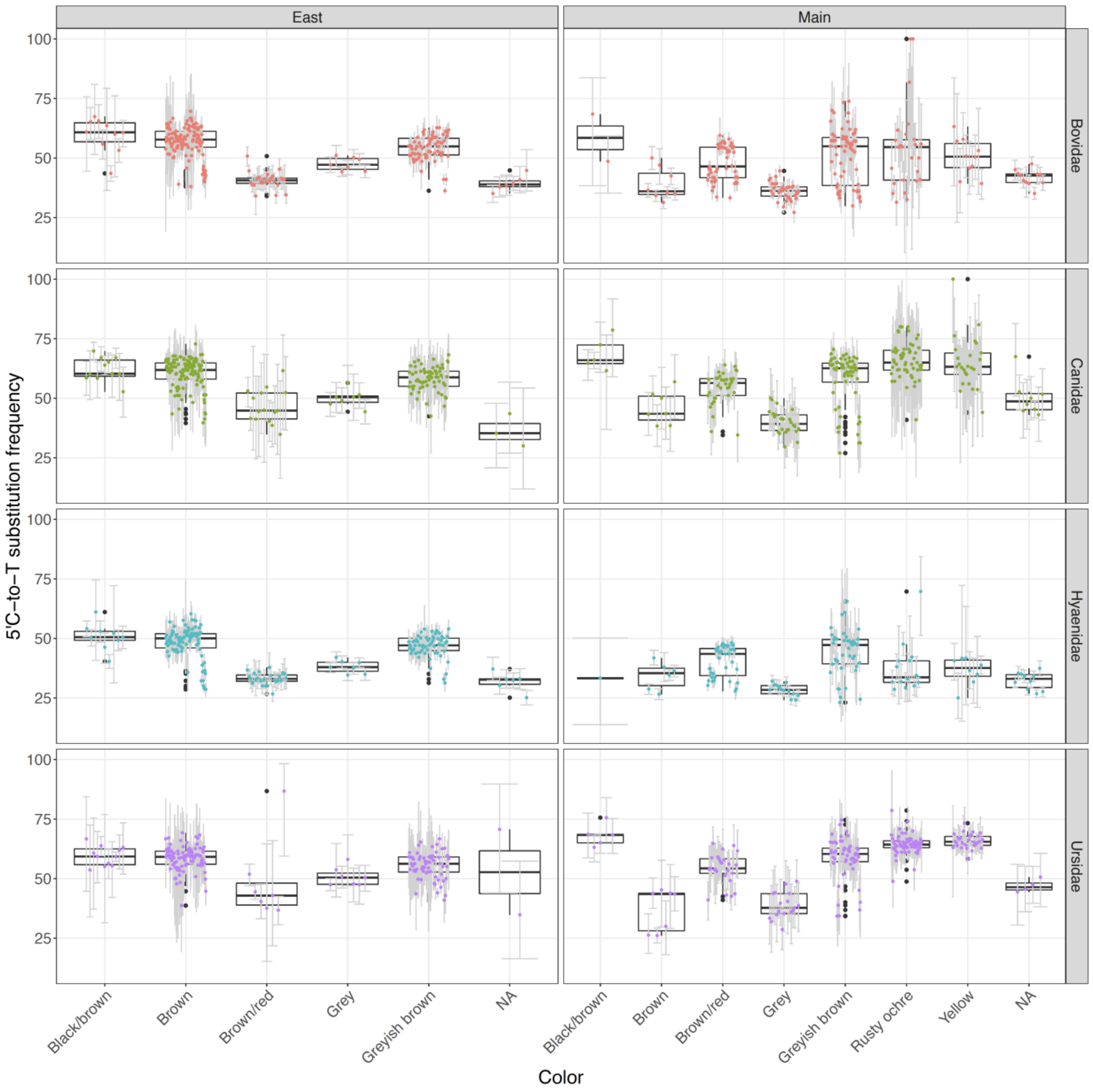

Supplementary Figure 10 | Inferred 5' deamination rates of ancient sequences assigned to Bovidae (n=435), Canidae (n=442), Hyaenidae (n=334) and Ursidae (n=393) across the layers in East and Main Chambers stratified by the recorded colour of sediments (black/brown, n=16; brown, n=131; brown/red, n=72; grey, n=39; greyish brown, n=132; rusty ochre, n=64; yellow, n=29). Each point (centre of error bar) represents the average observed 5' C to T substitution frequency in a library from a specific layer in the relevant chamber. The grey bars represent the 95% binomial confidence intervals based on the number of alignments starting at a C in the reference genome. The box plots show the distribution of the observed 5' C to T substitution frequencies following the standard Tukey representation (box limits represent the lower and upper quartiles, whiskers represent 1.5 times the interquartile range, outliers are represented by black dots). The "NA" assignment indicates that there was no published information for this layer.

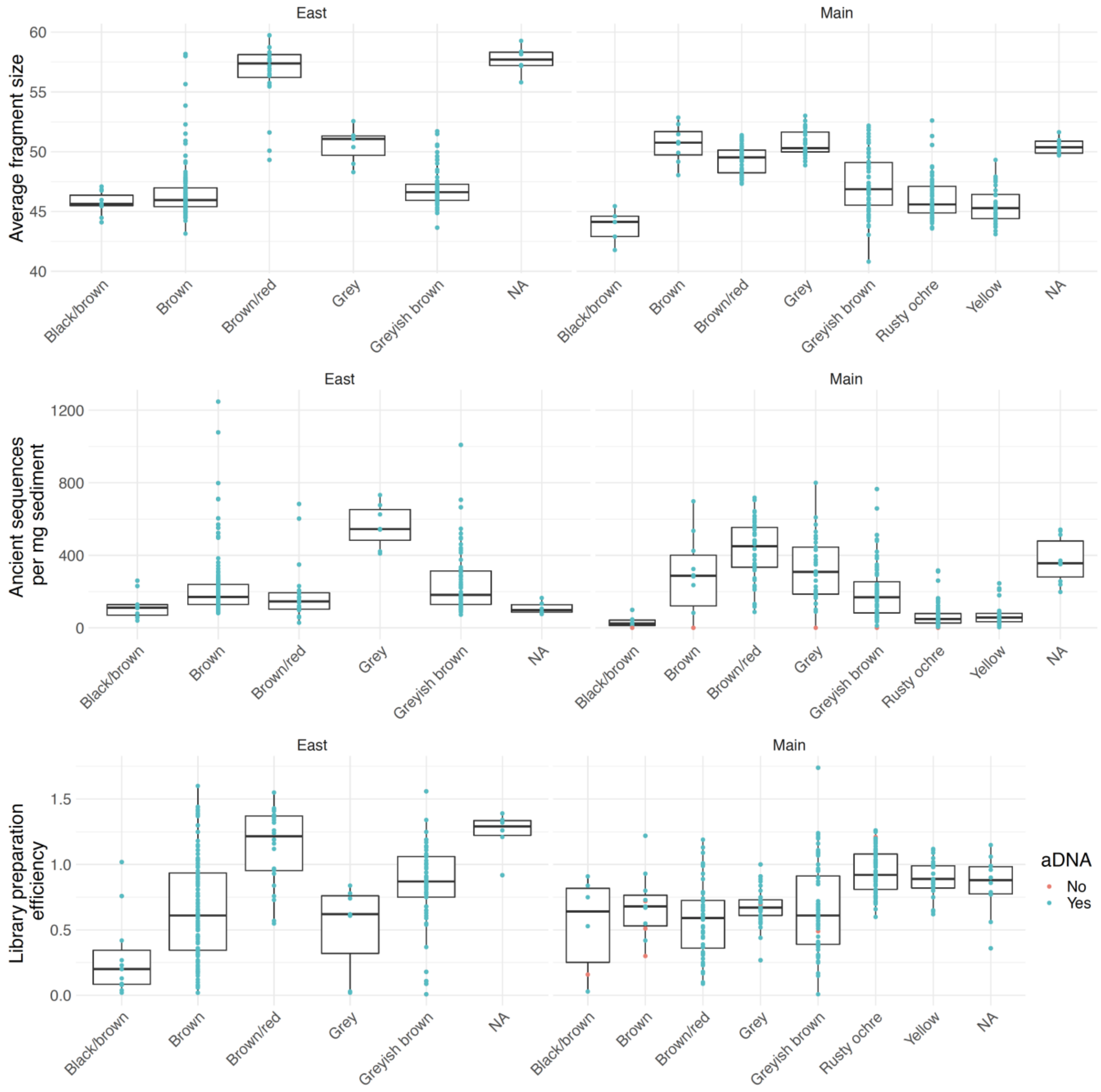

Supplementary Figure 11 | Average fragments size, the number of ancient mtDNA fragments recovered per milligram sediment and library preparation efficiency in samples from East and Main Chambers stratified by the recorded colour of sediments (black/brown, n=16; brown, n=131; brown/red, n=72; grey, n=39; greyish brown, n=132; rusty ochre, n=64; yellow, n=29). Each point represents a single sample and is coloured depending on if that sample was identified as containing ancient DNA. The box plots show the distribution of the observed average fragment lengths, number of fragments assigned to ancient taxa per mg of sediment, and library preparation efficiency following the standard Tukey representation (box limits represent the lower and upper quartiles, whiskers represent 1.5 times the interquartile range, outliers are represented by black dots). The "NA" assignment indicates that there was no published information for this layer (n=164). Note that technical variation in qPCR measurements may lead to library preparation efficiencies greater than 1.

SUPPLEMENTARY SECTION 4: IDENTIFICATION OF HUMAN MITOCHONDRIAL LINEAGES

4.1 Identification of diagnostic positions

In order to identify different groups of hominins throughout the stratigraphy, we determined diagnostic positions in the hominin mtDNA tree, following a strategy described earlier [22]. We first created a multiple sequence alignment, using MAFFT[23], of 19 Neanderthal mtDNA genomes [24-31], including that of the Hohlenstein-Stadel (HST) Neanderthal (which was retained as a separate branch due to its divergence from other, 'typical', Neanderthal mtDNA genomes)[32], a Middle Pleistocene hominin from Sima de los Huesos [33], 4 Denisovans[5,34-36], a chimpanzee [37], a set of 311 present-day modern humans from a wide geographical distribution [26] and the revised Cambridge Reference Sequence (rCRS)[38] (see Supplementary Data File 3 for a list of genomes used). We then determined positions where all mtDNA genomes representing one branch in the tree show a base difference to all other mtDNA genomes. In order to maximize the number of diagnostic positions available without reducing the accuracy of lineage assignment, two parameters were explored when determining diagnostic positions. First, we included or excluded the chimpanzee, and second, we required either at least 99% or 100% of the 311 humans to share the same state in order to call a diagnostic position. The combination of these parameters led to four sets of diagnostic positions which were tested further (Supplementary Figure 12).

Each set of diagnostic positions was tested in two different ways. First, we determined the support for each branch using previously published sequence data from a sediment sample from Denisova Cave, which had been shown to contain Neanderthal DNA[4], as well as unpublished data from an ancient modern human bone. Sequences from hominin mtDNA fragments were isolated using the analysis pipeline described in Methods and the support for each branch was examined using fragments overlapping the diagnostic positions. Only mtDNA fragments carrying C-to-T substitutions at the 5' and/or 3' end (putatively deaminated fragments) were included in the analysis in order to deplete DNA fragments originating from present-day human contamination. With all four sets of diagnostic positions, we observed high support (greater than 96%) for the typical Neanderthal and modern human branches, respectively, and consistently less than 10% for all other branches, for most even less than 2% (Supplementary Figures 13 and 14). Based on the above test, we concluded that all four sets of diagnostic positions produced valid and nearly indistinguishable results. We therefore chose the set with the largest number of diagnostic positions for further analyses, i.e. the set that was generated without the chimpanzee and for which 99% of the modern human mtDNA genomes were required to agree on one base.

Second, the homogeneity of coverage along the mitochondrial genome was evaluated by merging all putatively deaminated sequences generated in this study from sediment samples that were identified as containing ancient hominin DNA and determining the coverage for each position in the mtDNA genome. To evaluate the possibility of increased coverage at more conserved areas of the genome due to capture bias, we compared the observed coverage at each position to the phyloP conservation score [39-41]. A significant correlation between coverage and the phyloP score (Spearman's correlation test: rho = 0.0244, p-value = 1.7e-3) was found, but when we plotted a histogram of coverage at all diagnostic positions (determined without chimpanzee and requiring at least 99% of humans to be identical at the position) we observed only four positions with a coverage greater than 2 standard deviations from the mean. These diagnostic positions were removed to reduce imbalance in the contribution of individual positions in subsequent analyses (Supplementary Figure 15).

4.2 Assignment of mtDNA fragments to known hominin mitochondrial groups

Libraries that were identified as containing ancient hominin DNA were then evaluated for support of known hominin mitochondrial groups (human, Denisovan, Sima de los Huesos, HST-like Neanderthal, other Neanderthals). This was done by determining the proportion of all hominin mtDNA fragments sharing the group-specific state at positions diagnostic for the respective group. The support for each group was

computed twice: (i) using all fragments overlapping diagnostic positions and (ii) using fragments carrying a C-to-T substitution within the first or last three positions in their alignment to the rCRS (putatively deaminated fragments). In order to assign mtDNA fragments from a library to one or more groups, the following criteria had to be met: (i) Fragments had to support the group-specific state at three or more diagnostic positions, and (ii) significantly more than 10% of the fragments had to support the group-specific state based on 95% binomial confidence intervals. For the identification of modern humans, only deaminated fragments were used in order to minimize the impact of present-day contamination with modern human DNA.

In the initial screening, which included only one library per sediment sample, 124 of the 168 samples (74%) that showed evidence for the preservation of ancient hominin mtDNA could be assigned to specific hominin groups (Supplementary Data File 1). In order to understand why the lineage assignments were not resolved for all samples, the 44 libraries identified as containing ancient hominin DNA, but not assigned to a specific lineage were examined further. Thirty-two of these libraries contained high amounts of modern human contamination (≥ 50% based on the support for the modern human branch when using all fragments, i.e., without restricting to putatively deaminated fragments only), low numbers of hominin mtDNA fragments (≤40 deaminated fragments), and/or low-level support for multiple lineages (>5%, but non-significantly more than 10%), all of which likely hindered the ability to make a lineage assignment.

To test the reproducibility of identifying ancient hominin DNA within the same sediment sub-samples, we repeated the DNA extraction, library preparation, hybridization capture and sequencing on aliquots of lysates that originally produced libraries that tested negative (n = 19) and positive (n = 61) for ancient hominin DNA. Of the 61 samples that initially tested positive, 28 (46%) were positive in the second experiment (Extended Data Figure 6). However, our ability to replicate results varied strongly with the number of putatively deaminated DNA fragments that were recovered in the first screening: 16 out of 17 (94%) of the samples that initially yielded more than 100 fragments tested positive again (the sample that failed replication produced 105 fragments in the first screening), whereas positive results could be replicated for only 2 out of 31 (6%) of samples that showed less than 50 fragments in the first screening. Lineage assignments were consistent across experiments for libraries from the same lysates where these could be made, with the exception of a single lysate from the deformed Middle Palaeolithic portion of South Chamber (S149) that showed Denisovan support in the first and both Denisovan and Neanderthal support in the second library. Of the 19 lysates that initially tested negative, 5 (26%) turned positive in the second screening. These results indicate that the hominin DNA content of many samples is close to the detection limit, where our ability to detect ancient hominin DNA is hampered by stochasticity in the sampling of molecules, small fluctuations in the efficiency of sample preparation and variations in the amount of present-day human contamination that is introduced during laboratory work. It should also be noted that not all libraries from the second lysate aliquot were sequenced deeply enough (duplication rate > 3) to recover most of the unique fragments present in the library, which likely further impaired our ability to replicate positive results from the first screening.

After the sequencing of additional libraries produced to test for reproducibility, sequenced DNA fragments from each original lysate were merged for the remaining analyses (see Supplementary Data File 1 for more information on which samples had data merged). Based on these data, 142 of the 174 samples (82%) identified as containing ancient hominin mtDNA were assigned to a specific mitochondrial group. Of these, 45 were assigned to non-HST-like Neanderthals, 66 to Denisovans, 2 to both, 35 to modern humans and 2 to both modern humans and non-HST-like Neanderthals. No samples were assigned to HST-like Neanderthals or the Sima de los Huesos lineage.

The homogeneity of DNA preservation within samples was also tested by taking between two and seven additional sub-samples from 42 of the sediment samples that had been shown to contain ancient hominin DNA. All of the 7 samples that had more than 100 deaminated fragments in the initial library yielded hominin DNA in at least 50% of the sub-samples. In contrast, this was the case for only 10 out of 20 samples

(50%) that yielded less than 50 deaminated fragments in the first library that was prepared. The lineage assignments were not always consistent for sub-samples of the same sediment samples, especially for those with low amounts of deaminated fragments in the original library (Extended Data Figure 6c). These observations indicate that even though some samples tend to produce richer libraries upon repeated sub-sampling than others, preservation of ancient hominin DNA varies substantially within samples.

When combining all data, 244 sediment sub-samples (out of a total of 868) were found to contain ancient hominin DNA, of which 194 (80%) could be assigned to a lineage. From these 194 sediment sub-samples, 79 were assigned to non-HST-like Neanderthals, 87 to Denisovans, 6 to both Neanderthals and Denisovans, 2 to both modern humans and Neanderthals, 36 to modern humans, and none to HST-like Neanderthals or the Sima de los Huesos lineage (see Figure 1 and Extended Data Figure 3 for an overview of lineage support across the layers of all three chambers).

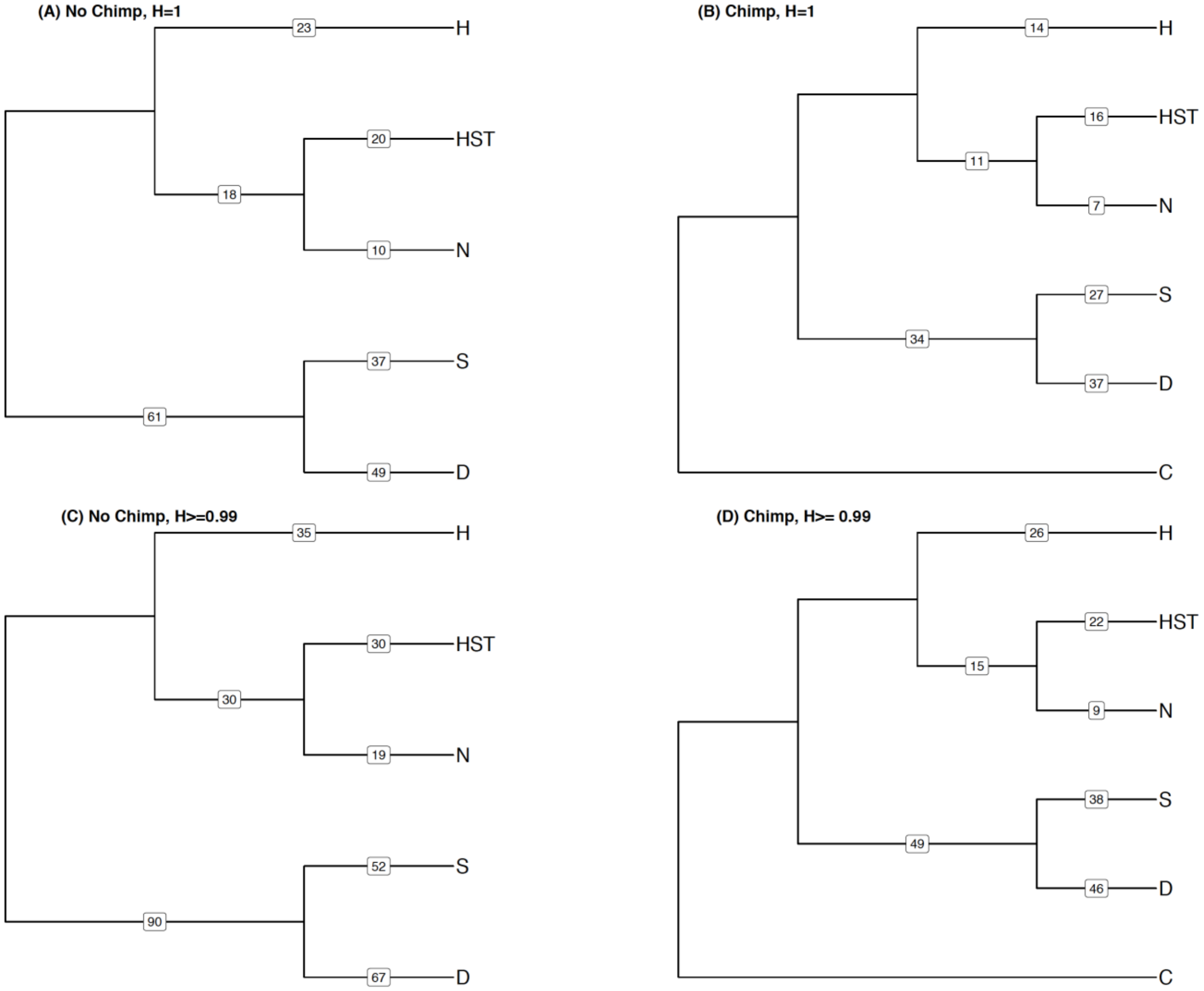

Supplementary Figure 12 | Number of diagnostic positions determined for each branch in the hominin mtDNA tree, with and without using chimpanzee as outgroup ('Chimp'/'No chimp') and requiring at least 99% (0.99) or all (1) modern human mtDNA genomes to be identical at a diagnostic position. H: modern humans; HST: Hohlenstein-Stadel Neanderthal; N: other Neanderthals; S: Sima de los Huesos; D: Denisovans.

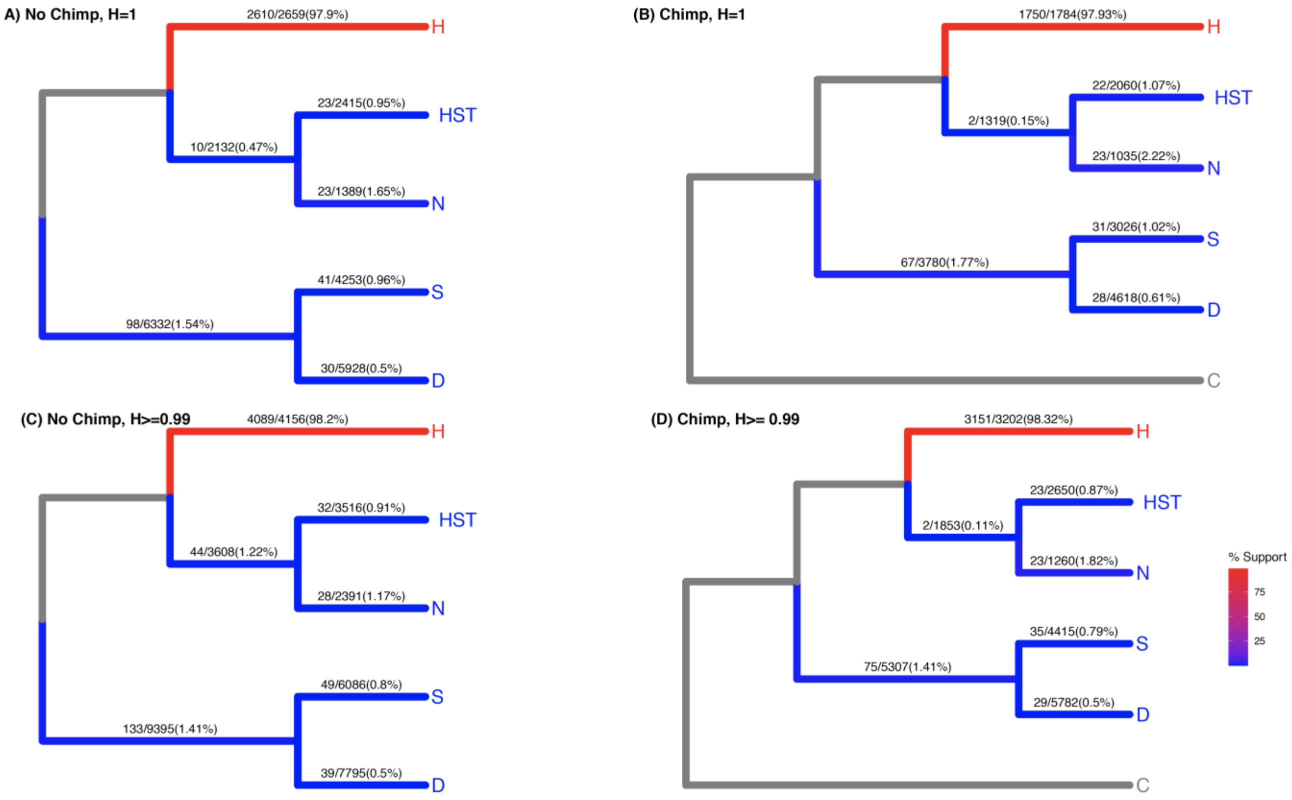

Supplementary Figure 13 | The observed support for different hominin mitochondrial lineages using four different sets of diagnostic positions and deaminated sequences from an unpublished early modern human bone. Each branch is coloured according to the support of the derived state. The number of fragments sharing the derived state, the total number of fragments overlapping lineage-specific diagnostic sites and the branch support are printed in red. H: modern humans; HST: Hohlenstein-Stadel Neanderthal; N: other Neanderthals; S: Sima de los Huesos; D: Denisovans.

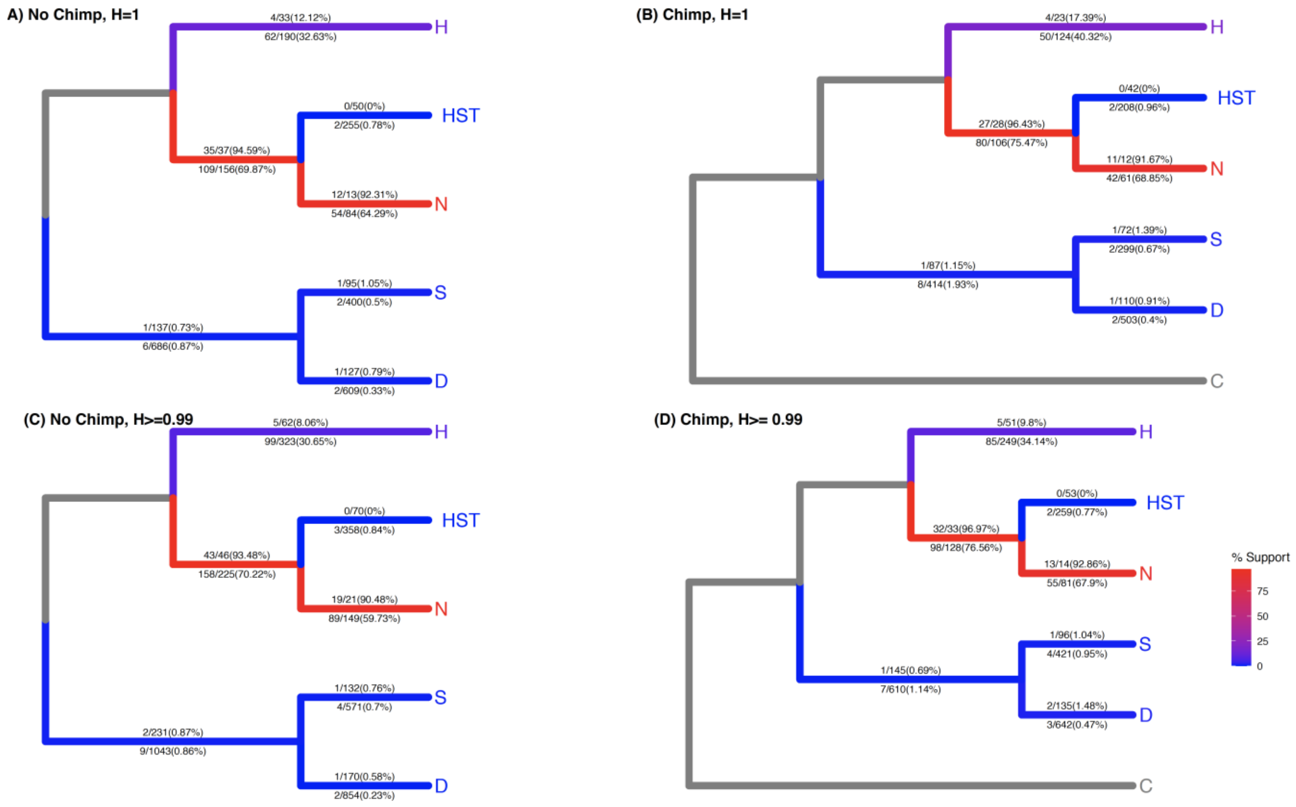

Supplementary Figure 14 | The observed support for different hominin mitochondrial lineages using four different sets of diagnostic positions and deaminated sequences from Denisova cave published in Slon et al, 2017 (D5276). Each branch is coloured according to the support of the derived state. The number of fragments sharing the derived state, the total number of fragments overlapping lineage-specific diagnostic sites and the branch support are printed in red. H: modern humans; HST: Hohlenstein-Stadel Neanderthal; N: other Neanderthals; S: Sima de los Huesos; D: Denisovans.

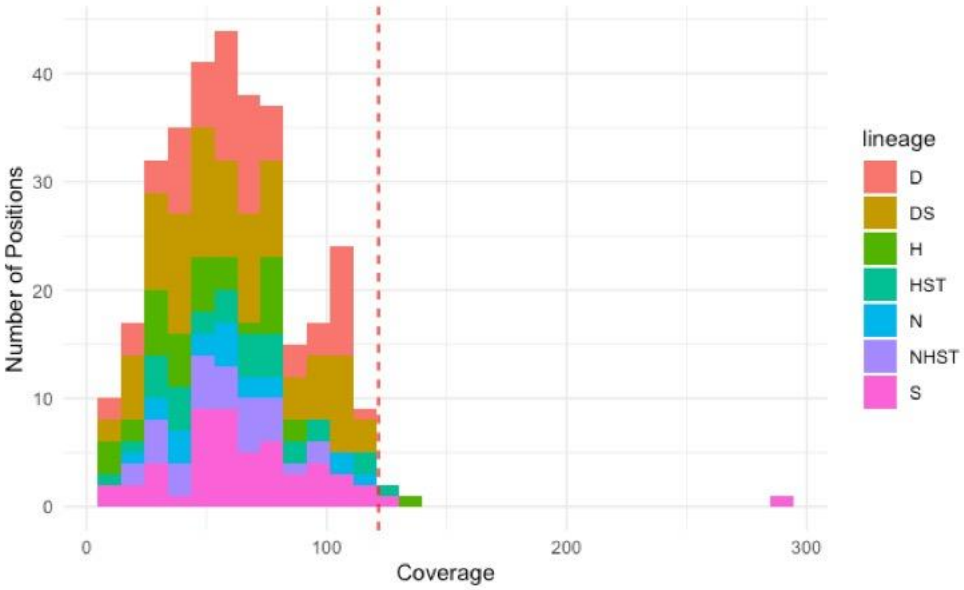

Supplementary Figure 15 | Histogram of the coverage of diagnostic positions for all deaminated reads mapped to the human mtDNA genome from libraries identified as containing ancient hominid DNA. Diagnostic positions were determined without using chimpanzee as outgroup and requiring at least 99% modern human mtDNA genomes to be identical at a diagnostic position. The red line represents two standard deviations from the mean coverage. The coverage is separated into 30 equal bins for the range of observed coverage (9 to 289). H: modern humans; HST: Hohlenstein-Stadel Neanderthal; N: other Neanderthals; S: Sima de los Huesos; D: Denisovans.

SUPPLEMENTARY SECTION 5: DETERMINING THE NUMBER OF SEQUENCE VARIANTS

Previous work on hominin mtDNA from sediments from Denisova Cave has shown that in most cases multiple individuals contributed to the DNA that was isolated from sediment. However, in one instance a single sequence variant was recovered, likely originating from only one individual[4]. We thus aimed to determine whether additional such cases can be detected in the much larger data set generated in the present study. We repeated the processing of hominin mtDNA sequences from sub-samples identified as containing either Neanderthal and Denisovan DNA using the Vindija 33.19 Neanderthal mtDNA genome (NC 011137.1) and the Denisova 3 mtDNA genome (NC_013993.1), respectively, as reference for mapping instead of the rCRS in order to minimize the loss of fragments due to sequence divergence to the reference. We estimated present-day human contamination based on the support for the modern human lineage in all fragments (Supplementary Section 4). For sub-samples with less than 5% estimated present-day human contamination all mtDNA fragments were used, for sub-samples with more than 5% estimated present-day human contamination putatively deaminated fragments were used. We then computed the mtDNA coverage from each sub-sample. Only sub-samples with at least 3x coverage (n = 7) were used for estimating the number of sequence variants (Supplementary Table 7).

We then used a previously published maximum likelihood method[4] that uses the consistency of the observed bases at each position to estimate the likelihood for a given library to contain one, two or three sequence variants. Hominin DNA fragments from three samples were estimated to originate from single contributors. In the remaining four samples, two sequence variants are most likely present (Supplementary Table 7). In all cases, it was estimated that the major sequence variant makes up at least 90% of the sequences generated from each sample. We therefore used all seven libraries for consensus calling (Supplementary Section 6) and reconstructing phylogenetic relationships with previously published mtDNA genomes (Supplementary Section 7).

Field sample ID	Sample ID	Data Source	Chamber	Layer	Lineage assignment	Average fragment size (bp)	Unique hominid sequences	Estimated depth of coveragae of mt genome (fold)	Support for human lineage (all fragments)	Number of deaminated fragments (based on 3 terminal bases)	Estimated deaminated depth of coveragae of mt genome (fold)	Support for human lineage (deaminated fragments)	Most likely number of contributors	Proportion of Contribution of major haplotype
S90	SP7214	Lysate	South	11	Human	55.6	11,258	37.76	99.0% (1126 / 1137)	1,686	5.65	100.0% (152 / 152)	1	NA
E254	SP7574	Lysate	East	11.1	Human	53.4	10,220	32.92	98.5% (980 / 995)	2,014	6.49	97.9% (191 / 195)	1	NA
E250	SP7570	Lysate	East	11.2	Human	54.2	8,412	27.50	97.3% (893 / 918)	2,200	7.19	95.8% (226 / 236)	1	NA
E213	SP7533	Lysate	East	11.4/12.1	Neanderthal	47.5	3,755	10.76	2.8% (6 / 212)	1,085	3.11	0.0% (0 / 59)	2	0.912
E202	SP7522	Library	East	11.4	Neanderthal	63	1,707	6.49	1.2% (2 / 165)	520	1.98	1.9% (1 / 52)	2	0.914
M65	SP6720	Lysate	Main	19	Neanderthal	50.3	9,093	27.59	4.2% (25 / 599)	2,810	8.53	3.1% (5 / 161)	2	0.915
M71	SP6726	Lysate	Main	20	Denisovan	48.8	7,699	22.66	21.2% (110 / 519)	2,439	7.18	6.3% (10 / 159)	2	0.909

Supplementary Table 7 | Estimated coverage and number of sequence variants present based on a maximum likelihood analysis for libraries with at least 3-fold coverage. The yellow highlights indicate when deaminated or all fragments were used for consensus calling. The support for the human lineage was used as a proxy for evaluating contamination. Data source indicates if data from a single library or merged data from the same lysate (sub-sample) were used.

SUPPLEMENTARY SECTION 6: RECONSTRUCTING ARCHAIC HUMAN MTDNA CONSENSUS SEQUENCES

To perform phylogenetic analyses, we attempted to reconstruct consensus genome sequences from the seven sub-samples identified as containing DNA predominantly from a single individual (Supplementary Section 5). For sub-samples containing Neanderthal or Denisovan DNA, alignments to the Denisova 3 or Vindija 33.19 mtDNA genomes, respectively, were used for consensus calling, while for samples containing ancient human mtDNA we used the alignment to the rCRS. Consensus calls were made at positions covered by at least 3 fragments if ≥60% of the fragments supported the majority base. In order to minimize the impact of deamination, T's on the terminal three bases of each fragment were disregarded. This strategy yielded mtDNA consensus sequences with between 217 and 6745 missing positions due to low coverage and up to 11 missing positions per sample due to low support of the majority base (Supplementary Table 8).

As the mtDNA capture probes were designed based on the human mtDNA genome, they may cause a bias towards modern human contamination in regions of the mtDNA genome where the divergence between modern and archaic humans is highest (the D-loop). Undetected contamination with mammalian mtDNA, on the other hand, is likely to be highest in the most conserved regions of the mtDNA genome (the 12S and 16S rRNA genes). We therefore decided to only use the 13 protein-coding genes of the mtDNA genome for further analysis to minimize the potential impact of erroneous consensus calls on phylogenetic reconstructions.

After limiting our analysis to the 13 protein-coding genes, we examined the missing positions that were due to low support of the majority base. We found that some of them were positions where some fragments and the reference genome carried a C and other fragments a T. This indicates that these positions likely remained unresolved due to deamination that occurred outside of the terminal three bases of DNA fragments. We manually corrected positions in the consensus sequences that showed low consensus support due to deamination in the 4th to 6th position at fragments ends (5 instances), resulting in consensus sequences with between 13 and 3889 missing calls due to low coverage and between up to 8 missing calls due to low consensus support for the approximately 11.3 kb of protein-coding genes in the mtDNA genome (Supplementary Table 8). Only the four consensus genome sequences that covered at least 80% of the mtDNA genome (three of Neanderthal and one of Denisovan type) were used for tree building (Supplementary Section 5). Among those, the most complete consensus sequence, a Neanderthal mtDNA sequence from layer 19 of Main Chamber, contained only one missing call due to low consensus support, indicating the consensus calling was not impacted by the presence of multiple mtDNA types. This consensus sequence was used for molecular dating (Supplementary Section 6). Attempts to determine haplogroups from the modern human mtDNA sequences using HaploGrep 2 (ref. 42) did not yield conclusive results. This is likely due to each sequence covering only about 60% of the mtDNA genome and/or because the ancient sequences fall basal to haplogroup-defining branches in the mtDNA tree.

Mitochondrial DNA									Fraction of mtDNA reconstructed						
									Complete mtDNA		Protein-coding region				
Field sample ID	Sample ID	Data Source	Chamber	Layer	Lineage assignment	Type of DNA fragments used for analysis	Estimated depth of coveragae of mt genome (fold)	Fraction of mt genome covered by reconstructed consensus sequences	Failed calls, low coverage (<3x)	Failed calls, low support (<60%)	Failed calls, low coverage (<3x)	Failed calls, low support (<60%)	Failed calls, low supp(<60%), after manual correction	Used for tree building	Used for molecular dating
S90	SP7214	Lysate	South	11	Human	Deaminated	5.65	0.59	6,745	2	3,889	1	1	Yes	No
E254	SP7574	Lysate	East	11.1	Human	Deaminated	6.49	0.63	6,118	6	3,814	6	3	Yes	No
E250	SP7570	Lysate	East	11.2	Human	Deaminated	7.19	0.62	6,331	8	3,805	5	5	Yes	No
E213	SP7533	Lysate	East	11.4/12.1	Neanderthal	All	10.76	0.89	1,891	5	711	3	3	Yes	No
E202	SP7522	Library	East	11.4	Neanderthal	All	6.49	0.82	2,966	0	1,540	0	0	Yes	No
M65	SP6720	Lysate	Main	19	Neanderthal	All	27.59	0.99	217	5	13	1	1	Yes	Yes
M71	SP6726	Lysate	Main	20	Denisovan	Deaminated	7.18	0.63	6,072	11	3,812	10	8	Yes	No

Supplementary Table 8 | Consensus calls for mitochondrial genomes from libraries identified as containing one major contributor. Data source indicates if data from a single library or merged data from the same lysate (sub-sample) was used.

SUPPLEMENTARY SECTION 7: BUILDING PHYLOGENETIC TREES

7.1 Constructing a Neighbour-Joining tree

To place the four consensus mtDNA genome sequences we reconstructed within the variation of hominin mtDNA genomes, we generated a multiple sequence alignment containing the mtDNA genome sequences of 55 present-day (from[26] and the rCRS) and 10 ancient modern humans[43-51], 24 Neanderthals[2,24-32], 4 Denisovans[5,34-36], and a chimpanzee[52] using MAFFT[23]. We then removed all non-protein-coding regions and positions containing gaps or missing data in one or more sequences. Trees were inferred using the Neighbour-Joining method in MEGAX[53], with 500 bootstrap replicates to estimate the support for each node. This analysis was carried out separately for the three Neanderthal and the one Denisovan mtDNA genomes reconstructed from sediment sub-samples (Extended Data Figure 6a,b).

All three newly reconstructed Neanderthal mtDNA genomes cluster together. This cluster falls outside of the Altai-like Neanderthal clade, and outside of the branches leading to other Neanderthal mtDNA genomes recovered from ancient individuals living in the Altai (Chagryskaya 8, Denisova 11 and Okladnikov 2), further emphasizing the Neanderthal mitochondrial diversity in the region during the late Pleistocene. The newly reconstructed Denisovan mtDNA genome falls outside of both the Denisova 2 and 8 and Denisova 3 and 4 clades, implying that there is more diversity in Denisovan mtDNA genomes than previously known within Denisova Cave.

7.2 Branch shortening estimates

One of the sub-samples (from sample M65, layer 19 of Main Chamber) produced a Neanderthal consensus mtDNA genome sequence of a sufficient quality to date it by molecular methods. We therefore constructed a phylogenetic tree using BEAST2[54] and the multiple sequence alignment described in the previous section but excluding other consensus sequences from sediment samples. We identified the best fitting clock and tree model for the analysis by using a path sampling approach from the MODEL_SELECTION package[55-57] in BEAST2[54]. For each model combination, 40 path steps were used, each with a chain length of 25,000,000 iterations, parameter alpha of 0.3, pre-burn-in of 75,000 iterations and an 80% burn-in of the whole chain. A mutation rate 1.57 X 10E-8 was used as the analysis was restricted to the protein-coding region. For both the relaxed log normal and strict clock models, a normal distribution was used with the mean set to the mutation rate mentioned above and a sigma of 1.00E-10[45]. For all models, the substitution model Tamura-Nei 1993 (TN93)[58] was used as this was estimated to be the best model for archaic hominins[32]. Previously published radiocarbon dates for ancient modern humans and Neanderthals [2,24-32,59] were used to calibrate the tree. All modern samples were set to present day (date = 0). The ages of Neanderthals of unknown age, including the sediment sample, were constrained to a range of 30,000 to 200,000 years, with the exclusion of Sima de los Huesos which was constrained to 200,000 to 780,000 years. As has been done elsewhere[32], *Denisova 3* was restricted to the range of 30,000 to 100,000 years based on previous molecular dating of its nuclear genome[60,61]. For the other Denisovans (*Denisova 2*, *4* and *8)*, ranges of 30,000 to 300,000 years were used. For each individual, we used a uniform prior over the allowed range of dates. Neanderthals, modern humans, and Denisovans were constrained to monophyletic groups and the time to their most recent common ancestor (TMRCA) was estimated for each group.

Both the strict and relaxed clocks with a constant population size were found to be significantly better than using a Bayesian skyline population model (Bayes factors[56] 7.8 and 6.08 for strict and relaxed clocks, respectively). No significant difference was found between the strict and relaxed clocks with a constant population (Bayes factor 0.76, Supplementary Table 9), therefore the combination of a strict clock and a constant population size was used as it is the simplest model. Three Markov chain Monte Carlo (MCMC) runs of 75,000,000 iterations, with a pre burn-in of 10,000,000 iterations and sampling every 2,000 trees were then performed. The log and tree files of the runs were then merged using logcombiner2 from BEAST2[54]. The merged tree file was subsequently annotated using the program treeannotater from

BEAST2[54], summarizing the output into a single tree. Tracer from BEAST2[54] and Figtree from BEAST2[54] were used to examine the resulting tip dates and TMRCA estimates (Supplementary Table 10 and Supplementary Figure 16). The estimated date for the mtDNA genome reconstructed from sediment sample M65, which was collected from layer 19 of Main Chamber, is 140 ka (highest posterior density, HPD: 98-181 ka). This coincides with the range of optical ages determined for this layer (151 ± 17 ka for the bottom of layer 19 and 128 ± 13 ka for the top of layer 17)[1].

Clock Model	Tree Model	Marginal log likelihood
Strict	Constant	-27327.1522
Strict	Bayesian Skyline	-27345.9693
Relaxed Log Normal	Constant	-27325.3955
Relaxed Log Normal	Bayesian Skyline	-27339.9102

Supplementary Table 9 | The marginal log likelihoods for the trees based on the mitochondrial protein-coding genes generated from testing different clock and tree models with a path sampling approach.

Parameter	Mean	95% HPD lower	95% HPD upper	ESS
Denisova2	189,630	119,360	260,380	1,324
Denisova3	72,671	40,852	99,999	666
Denisova4	78,658	38,423	111,870	440
Denisova8	150,240	74,658	224,550	1369
M65 (Denisova Cave, Main Chamber Layer 19)	139,860	97,890	181,050	1,638
Altai	135,490	87,815	184,490	1,405
Chagyrskaya08	87,989	52,162	123,690	2,738
Denisova11	113,140	82,937	144,280	1,736
Denisova15	130,360	82,592	180,490	1474
El Sidron	65,924	41,460	92,740	913
Goyet Q305-7	40,334	30,192	46,639	273
Goyet Q374a-1	40,345	30,302	46,751	265
HST	132,990	71,798	199,980	2,293
Mezmaiskaya 1	94,653	47,960	140,900	2,320
Okladnikov2	107,740	76,339	140,480	2,138
Scladina	120,220	70,277	170,850	1,617
Vindija 33.17	53,566	38,668	66,964	375
Vindija 33.19	45,168	32,418	55,784	325
Vindija 33.25	45,423	31,561	59,274	384
Sima de los Huesos	346,850	212,070	470,580	660
TMRCA Denisovans + Sima	671,210	575,160	768,290	891
TMRCA Humans	177,770	145,860	210,760	26,471
TMRCA Neanderthals	311,540	260,960	363,020	2,649

Supplementary Table 10 | The estimated tip dates and divergence times (in years) reported from the Tracer program from BEAST2 using a strict clock and constant population size for the protein-coding region. HPD, highest posterior density; ESS, effective sample size.

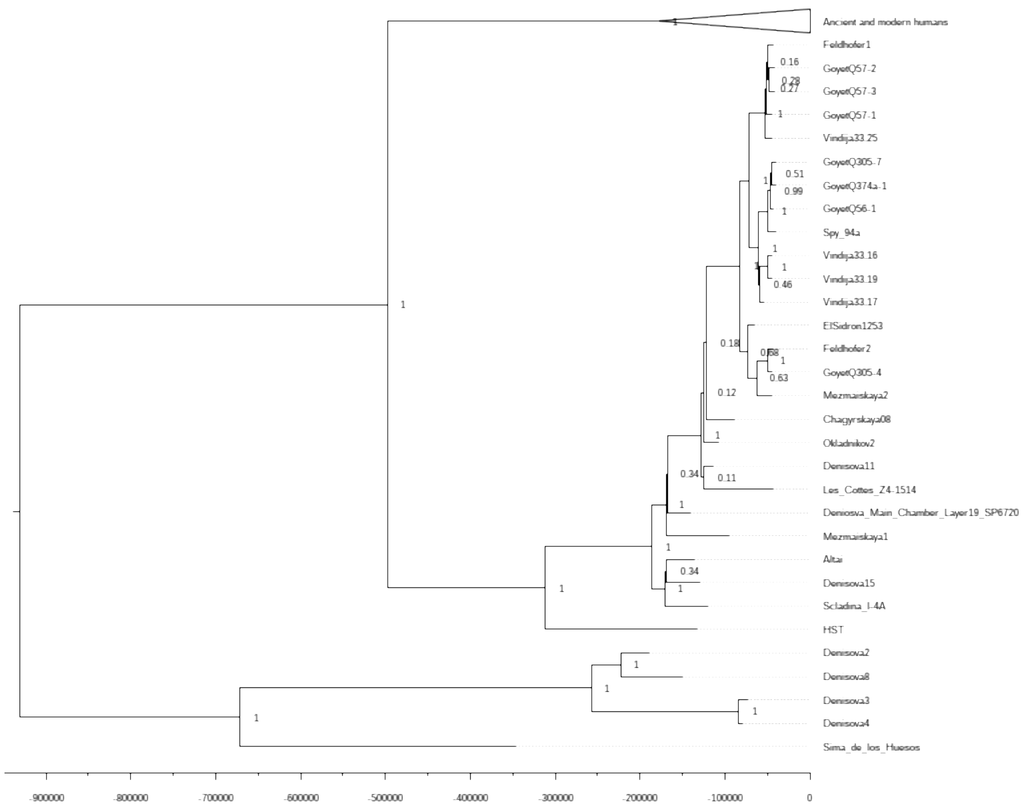

Supplementary Figure 16 | The phylogenetic mtDNA tree determined from Bayesian analysis with Beast2 using approximately 11.3 kb of protein-coding genes from the mitochondrial genome. Each node shows the corresponding posterior probability of the branch and the x-axis represents the time in years from the present. The chimpanzee branch used to root the tree is not shown.

SUPPLEMENTARY SECTION 8: MTDNA HAPLOTYPE IDENTIFICATION FROM SPARSE DATA USING KALLISTO

As described in Supplementary Section 5, many sediment libraries contain low coverage of the mtDNA genome, or appear to contain mtDNA from multiple haplotypes, limiting the ability to construct consensus sequences. In this study, DNA from 244 sub-samples could be assigned to either the modern human, Neanderthal or Denisovan lineage via diagnostic sites (Supplementary Section 4); of which only 7 were suitable for constructing a consensus sequence of the mtDNA genome (Supplementary Section 4). Here, we use a method based on the program kallisto[62] to assign a library (or its major component in case of mixtures) to specific branches in the hominin mtDNA tree, extending the categorization of these samples beyond the identification of broad hominin groups.

We first constructed a multiple sequence alignment (MSA) for the full length of 95 mtDNA genomes, comprised of: 25 Neanderthals (including *Denisova 11*), one Neanderthal consensus sequence inferred from Denisova cave sediment sample M65 (Supplementary Section 6), 4 Denisovans, *Sima de los Huesos*, 54 modern humans and 10 ancient modern humans, as described in Supplementary Section 6 (full list of genomes in Supplementary Data File 3). To aid in analysis, we have partitioned the tree into sets of "major groups" that are closely related to each other (Supplementary Figure 17, node colours; Supplementary Figure 18, red boxes). Each of these groups is composed of mtDNA genomes which form a clade, or diverge from the larger tree at around the same time. When constructing kallisto references out of tip mtDNA genomes, we first remove all columns from the MSA in which any genome has an unknown base (i.e., an "N").

We next simulated 5,000 ancient DNA reads from each of the Sima, Neanderthal or Denisovan mtDNA genomes, as described in Supplementary Section 9. In order to simulate DNA from the entire mtDNA genome – that is, to avoid removing N columns – we inferred ancestral states across the full phylogeny (Supplementary Figure 17) with the software treetime[63], using a TN93 mutation model and a mutation rate of 1×10^{-10} bases per year. These ancestral states then resolve any unknown bases in the tip genomes.

We then tested the ability of kallisto to correctly place simulated DNA reads. Using the full set of mtDNA genomes as a reference set, we find that the correct genome is always the most abundant hit, receiving approximately 80-95% of the total estimated abundance (Supplementary Figure 18). The only exception is when multiple genomes are identical, in which case the abundance is equally distributed between them (e.g., Goyet individuals).

A more realistic scenario is one where the correct genome is now known – i.e., because the aDNA originates from a new sediment or skeletal sample, and is a novel mtDNA haplotype. We therefore repeated this analysis, each time dropping the source genome from the set of reference genomes. In almost all cases, the reference genome with the highest abundance is closely related to the source genome, and falls within the same major group (Supplementary Figure 19). The primary exceptions are *HST* and *Sima de los Huesos*, which have no closely related genomes – in these cases, the abundance falls on a distantly related genome. Some mtDNA references seem to "attract" abundance from distantly related genomes (e.g. Chagyrskaya 8 often has low levels of abundance - up to 21% of all non- modern human abundance), and thus may be more challenging to classify.

8.2 Probabilistic genome assignment with ancestralized mitochondrial genomes

We next used the method from Vernot et al.[64] to assign each sample to the mtDNA phylogeny (Supplementary Figure 17). In short, we simulated DNA from ancestralized genomes spanning the phylogeny, and for each reference we calculated abundance thresholds at which there is a >90%, 95% or 99% probability that the DNA originated from a closely related genome. Here, "closely related" is defined as on the same branch, or within 50,000 years of the root of the branch. These thresholds vary based on the reference (Supplementary Figure 20), with some references (e.g. Chagyrskaya 8, discussed above) requiring larger proportions of the total abundance for there to be considered strong evidence of a closely related

genome. These thresholds vary depending on the number of reads used in the analysis, with larger amounts of reads generally requiring lower abundance to reach given certainty. We therefore calculated thresholds for each reference using 100, 150, 200, 250 and 1000 reads (Supplementary Figure 20, upper and lower rows show thresholds for 250 and 1000 reads), and for each of these 90, 95 and 99% thresholds (Supplementary Figure 20, three dotted red lines, from bottom to top). The black dotted line in Supplementary Figure 20 shows an evolutionary distance of 50,000 thousand years.

We then calculated the number of ancient hominin reads for each sub-sample as (1 - modern human contamination) * (number of unique reads). The modern human contamination proportion was calculated as described in Supplementary Section 5, using diagnostic positions. Finally, we ran kallisto on all sediment sub-samples (Supplementary Figure 21) with >100 ancient hominin reads. For each sample we calculated the proportion of non- modern human abundance assigned to each reference, under the assumption that any modern human signal largely originates from contamination. To convert these proportions in to probabilities, we applied the thresholds as calculated above. For samples with 250-999 ancient hominin reads, we conservatively use the 250 read thresholds. The results are presented in Supplementary Figure 22 and shown within the stratigraphy in Main Figure 1 for East and Main Chambers and Extended Figure 3 for South Chamber.

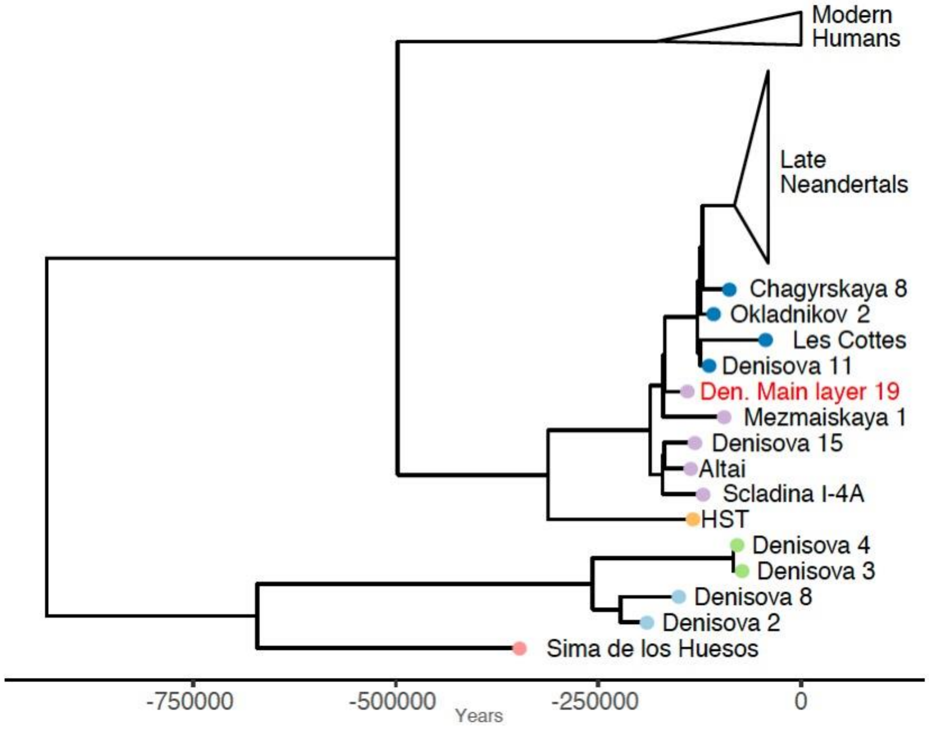

Supplementary Figure 17 | mtDNA phylogenetic tree of 95 hominin mtDNA genomes. mtDNA genomes include: 25 Neanderthals (including *Denisova 11*), one Neanderthal consensus sequence inferred from Denisova cave sediments (red text), 4 Denisovans, *Sima de los Huesos*, 54 modern humans and 10 ancient modern humans.

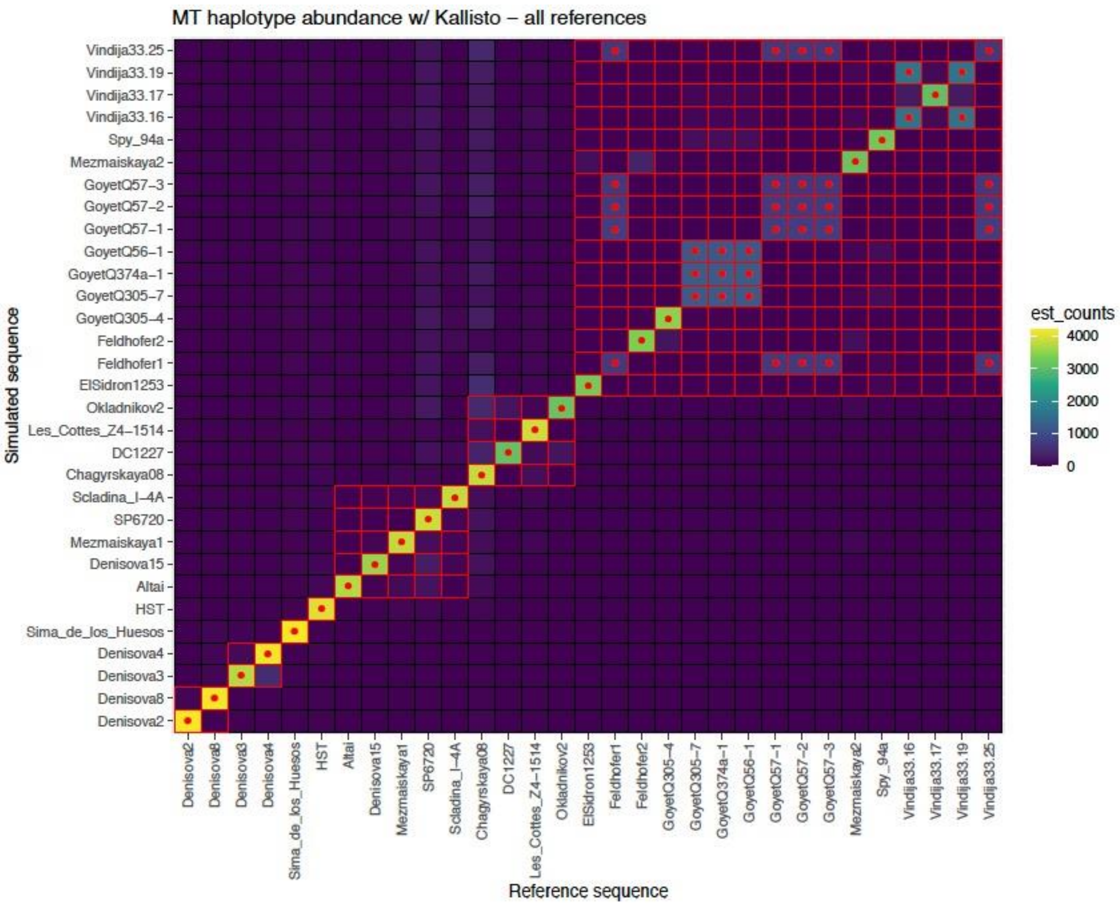

Supplementary Figure 18 | kallisto abundance estimates for simulated mtDNA with full knowledge of reference genomes. Tile colour is estimated abundance. Genome from which aDNA was simulated shown on y-axis, and reference genomes are on the x-axis. Reference genomes are the same as in Supplementary Figure 17. DC1227 is Denisova 11, and SP6720 (M65) is a sediment consensus sequence from Denisova Cave Main Chamber layer 19. Red boxes denote major mtDNA groupings, and are the same as groupings in Supplementary Figure 17. Red dots denote the highest abundance reference(s).

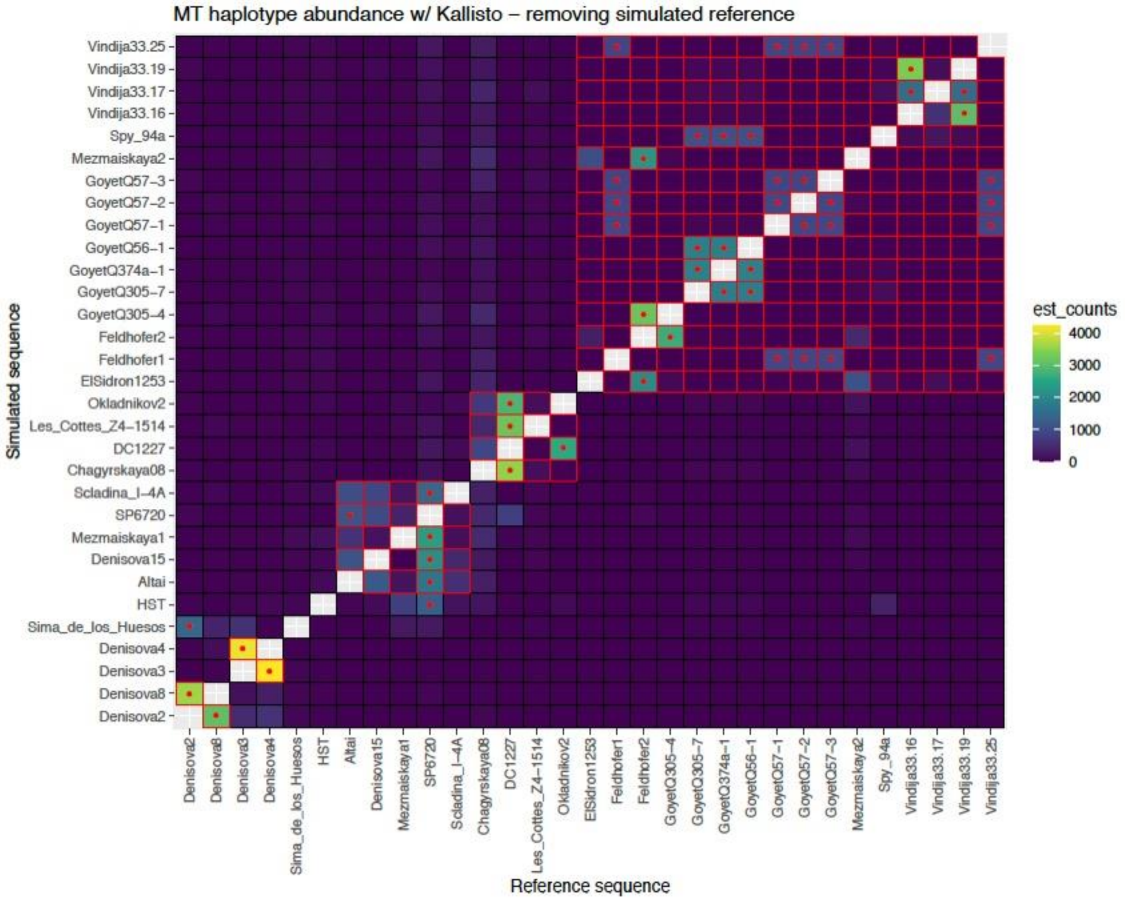

Supplementary Figure 19 | kallisto abundance estimates for simulated mtDNA with simulated genome removed from reference genomes. Tile colour is estimated abundance. Genome from which aDNA was simulated shown on y-axis, and reference genomes are on the x-axis. Reference genomes are the same as in Supplementary Figure 17. DC1227 is Denisova 11, and SP6720 (M65) is a sediment consensus sequence from Denisova Cave Main Chamber layer 19. Red boxes denote major mtDNA groupings, and are the same as groupings in Supplementary Figure 17. Red dots denote the highest abundance reference(s).

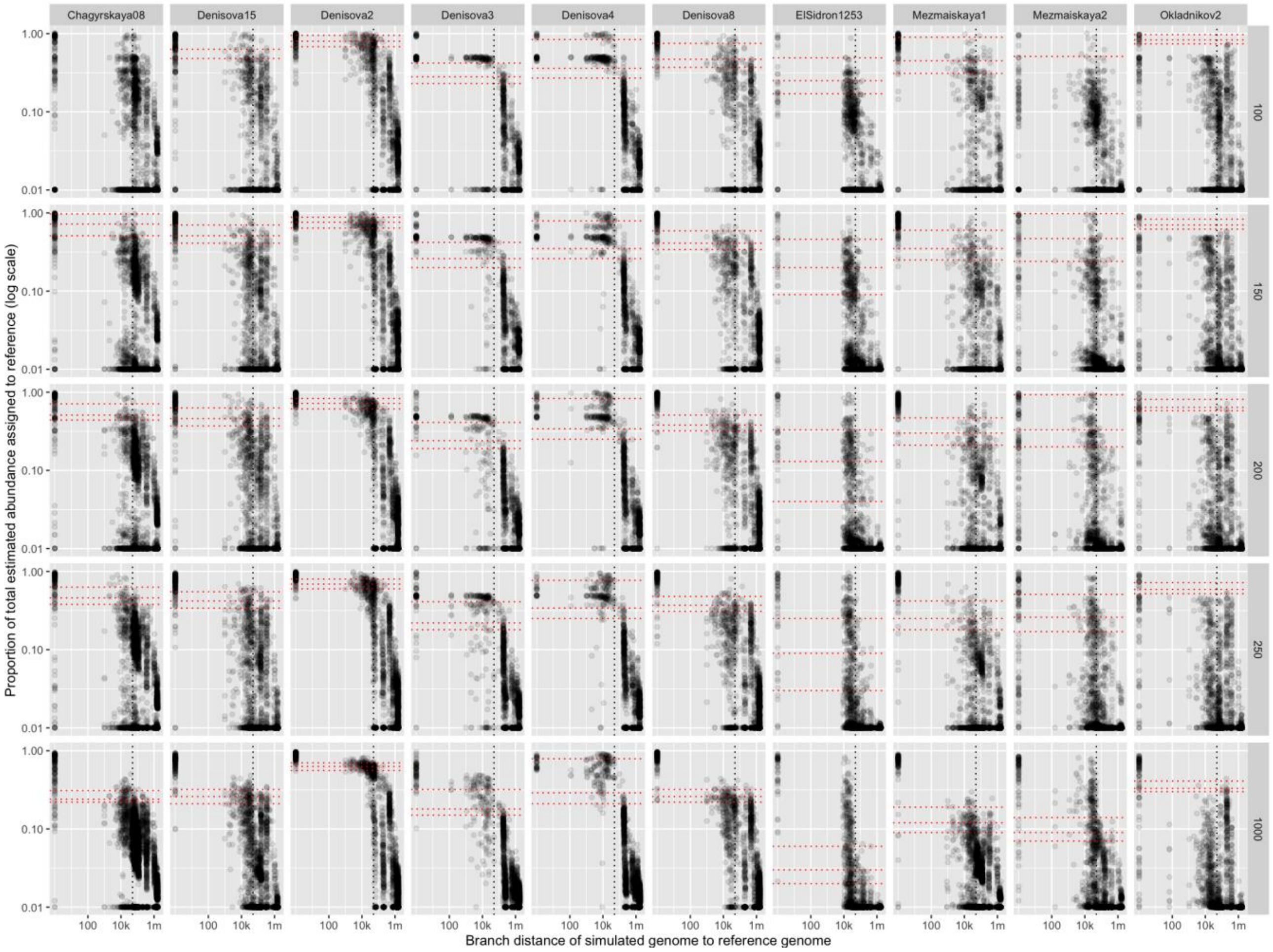

Supplementary Figure 20 | Example abundance thresholds for 10 mtDNA references. Normalized abundance for each of 10 reference genomes (columns), for simulated ancient DNA from ancestralized genomes spanning the mitochondrial tree. Each point is one simulated mtDNA genome, and its abundance for that particular target. Red vertical line at 50,000 years divergence – points to the left of this line are from ancestralized genomes that are less than 50,000 years diverged from the reference genome branch, and are considered "closely related". For each reference, three abundance thresholds *X* were calculated (red dotted lines), such that 90%, 95% and 99% (bottom to top) of all simulated sequences with at least *X* abundance are more closely related than 50,000 years. Thresholds shown for 100-1000 simulated reads (rows).

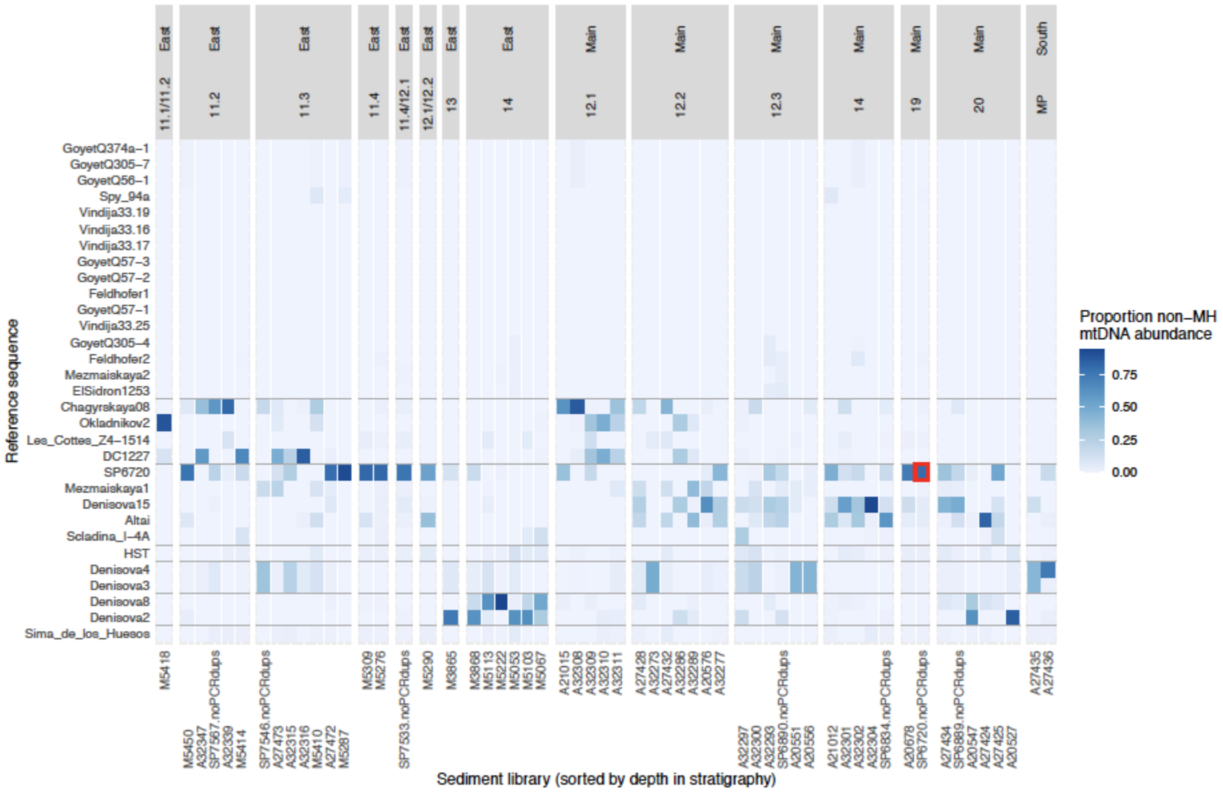

Supplementary Figure 21 | kallisto mtDNA abundances for Denisova Cave sediments. kallisto abundances for all Denisova Cave sediment sub-samples with at least 100 ancient hominin DNA fragments. Abundances are normalized to the total non- modern human abundance. Reference sequences are the same as in Supplementary Figure 18 and 19. DC1227 is Denisova 11, and SP6720 (M65) is a sediment consensus sequence from Denisova Cave Main Chamber layer 19. Sub-samples from the same sample grouped by white lines. Grey horizontal lines denote major phylogenetic groups.

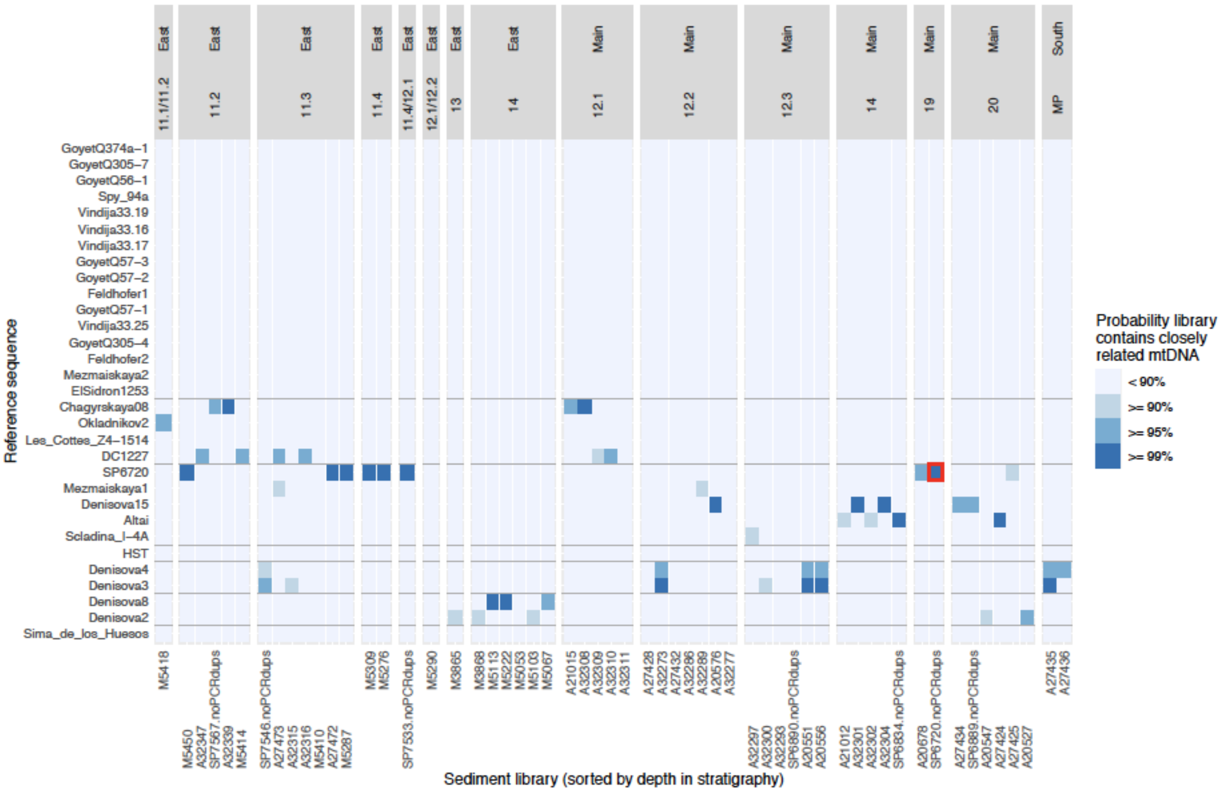

Supplementary Figure 22 | Probabilistic phylogenetic placement of sediment samples. Probabilistic phylogenetic placement of mtDNA from 57 sediment sub-samples from 37 sediment samples. Sub-samples from the same sample grouped by white lines. Grey horizontal lines denote major phylogenetic groups. The sub-sample of SP6720 (M65) from which the consensus haplotype was inferred is denoted with a red box.

SUPPLEMENTARY SECTION 9: IDENTIFICATION OF PREVIOUSLY UNKNOWN HOMININ MTDNA LINEAGES

Even though diagnostic positions have to be defined using known mtDNA sequences (which are also required for analyses based on kallisto), archaic hominin mtDNA lineages that are yet unknown can in principle also be detected by identifying sub-samples with relatively high numbers of hominin deaminated fragments that could not be assigned to any of the known lineages. To investigate whether there is evidence for the presence of such unknown lineages in our data, we plotted the distribution of the number of deaminated fragments in sub-samples that were identified as containing ancient hominin mtDNA, but which were not assigned to any lineage (n = 51). All except two sub-samples contained less than 100 putatively deaminated fragments, compatible with that their assignment to a hominin lineage was prevented by limited statistical power. However, two sub-samples (lysate IDs: Lys565 and Lys10333), both originating from sample M76, contained 246 and 770 deaminated fragments (996 and 2646 unique hominid sequences), respectively, more than any of the other unassigned sub-samples (Supplementary Figure 23).

A closer examination of the support of diagnostic positions observed for these two sub-samples revealed significantly more than 10% support (based on 95% binomial confidence intervals) for the branch shared by all Neanderthals (i.e., the shared Neanderthal-Hohlenstein-Stadel branch, subsequently referred to as 'N-HST' branch; 31-33% support), but not for the HST branch (0.5-1.4% support) or the typical Neanderthal branch (8-13% support) (Supplementary Figure 24). Support for the N-HST branch increases to between 39% and 45% when using deaminated fragments only, while support for the HST branch and typical Neanderthal branch remains low (0% and 13-18%, respectively), suggesting the presence of Neanderthal mtDNA that shares only few derived sites with known Neanderthal mtDNA genomes. In addition to Neanderthal mtDNA, both sub-samples show significantly more than 10% support for the modern human branch (40-54%), which reduces to insignificant levels after filtering for deaminated fragments (3-14%), indicating contamination with present-day human DNA. Both sub-samples also show low but insignificant levels of support for the Denisovan branch, including after filtering for deaminated fragments (6-9% and 6-15%, respectively).

To investigate if the observed difference in branch support between the internal (N-HST) and external (N) Neanderthal branches is unique to these sub-samples, we examined all sub-samples presenting significant support for the internal (N-HST) branch and containing at least 100 deaminated fragments and tested whether there is a significant difference in support between the two branches. In order to maximize the amount of available data this comparison was performed using all fragments. Of the 37 sub-samples where this test could be performed, only the aforementioned sub-samples originating from sample M76 showed a significant difference of support between the two branches (Fisher exact test with a BH correction[65] for multiple testing, Lys565 p-value = 1.5E-2 and Lys10333 p-value = 1.2E-03). Using the percent support for the modern human mtDNA lineage as an estimation of contamination, these 37 sub-samples contained a range of present-day human contamination from 5% to 88% with a mean of 29%. The sub-samples from M76 contained an estimated 40 and 54% of human mtDNA contamination, indicating that this signal is not due to modern human contamination.

In order to test if the presence of both Neanderthal and Denisovan mtDNA in one sample (as likely is the case for M76) could lead to an imbalance in support between the internal (N-HST) and external (N) Neanderthal branches, we also examined 10 sub-samples that contained mtDNA from both groups. These sub-samples contained between 27 and 403 deaminated fragments (114 and 2,150 unique hominid sequences) and 0-52% present-day human mtDNA contamination. Based on a fisher exact test no libraries showed a significant difference in support between the two branches, indicating the presence of both Neanderthal and Denisovan mtDNA in a sample does not result in an imbalance of support between the external and internal branches.

We next investigated whether the increased support for the N-HST branch displayed by sample M76 is consistent with a mitochondrial genome sequence falling basal to the known mtDNA diversity of known

Neanderthals. To do this, we simulated six ancestralized mtDNA sequences based on the Altai Neanderthal (*Denisova 5*) mtDNA genome, going back to six time points between 40,000 and 200,000 years before the Altai Neanderthal lived (approximately 130 ka). This was done using a tree of published mitochondrial genomes constructed from Bayesian analysis with Beast2 [54] and the program treetime[63], following the procedure outlined in Supplementary Section 8. Briefly, the Tamura-Nei 1993 (TN93)[58] mutation model was used with a mutation rate of 1e-10 mutations per base pair per year. The tree built using Beast2 estimated the time between the tip point of the Altai Neanderthal mtDNA genome to the shared node between all non-HST Neanderthals at 39,700 years and to the shared node between all Neanderthals, including HST, at 145,000 years (Supplementary Figure 25). The output of this program was a single fasta file which was then used to generate different numbers of simulated reads (100, 300, 500, 1000 and 2,000) mimicking ancient DNA fragments, using a read length distribution observed in a sample previously enriched for human mitochondrial DNA via hybridization capture (limits of 30 bp and 100 bp) and deamination rates as used in ref[61]. The simulated fragments were then processed as described in the Methods section.

The log ratio of support between the N and NHST branch was calculated for each set of ancestralized fragments and compared to the log ratio of the combined fragments from both sub-samples Lys565 and Lys10333 (Supplementary Figure 26). The difference in support between the shared N-HST branch and N branch for the sub-sample is consistent with a genome diverging from the N branch 100,000-125,000 years before Altai lived, i.e., between 20,000 and 45,000 years after the divergence of HST from other Neanderthals (255-230 ka). Sample M76 is from layer 20 of Main Chamber, which is dated to 250-170 ka [1]. The shared node between all Neanderthals (including HST) is dated at 275 ka (Supplementary Figure 25), implying that a Neanderthal mtDNA genome branching near this point would have a branch length of between 5000 and 85,000 years.

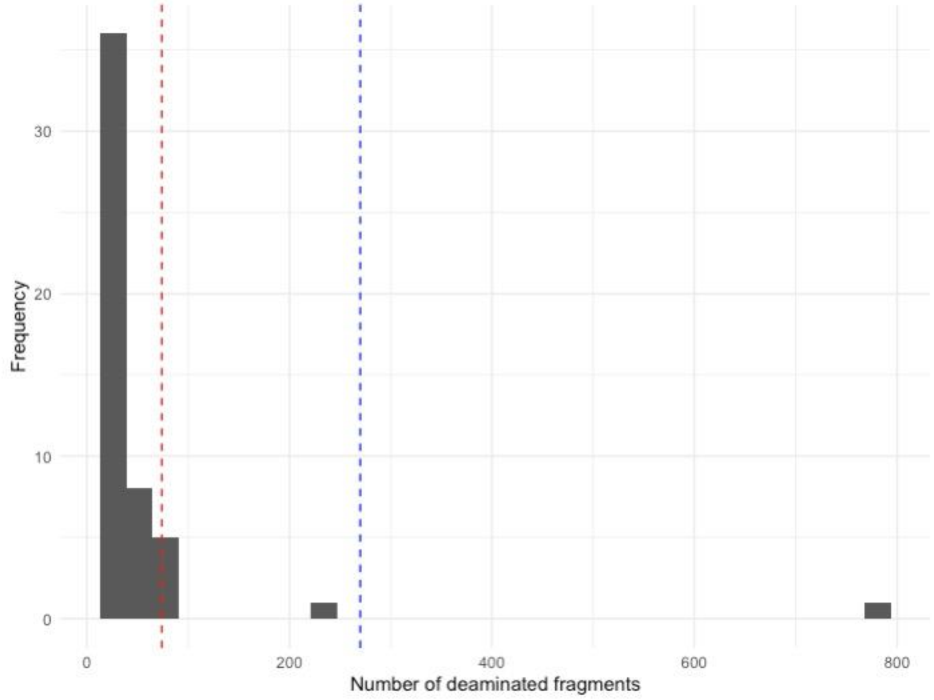

Supplementary Figure 23 | Histogram of the number of deaminated fragments in sub-samples containing ancient hominin DNA that were not assigned for a lineage. The dotted red line represents two standard deviations from the mean number of deaminated fragments for sub-samples containing less than 100 deaminated fragments. The dotted blue line represents two standard deviations from the mean number of deaminated fragments for all unassigned sub-samples.

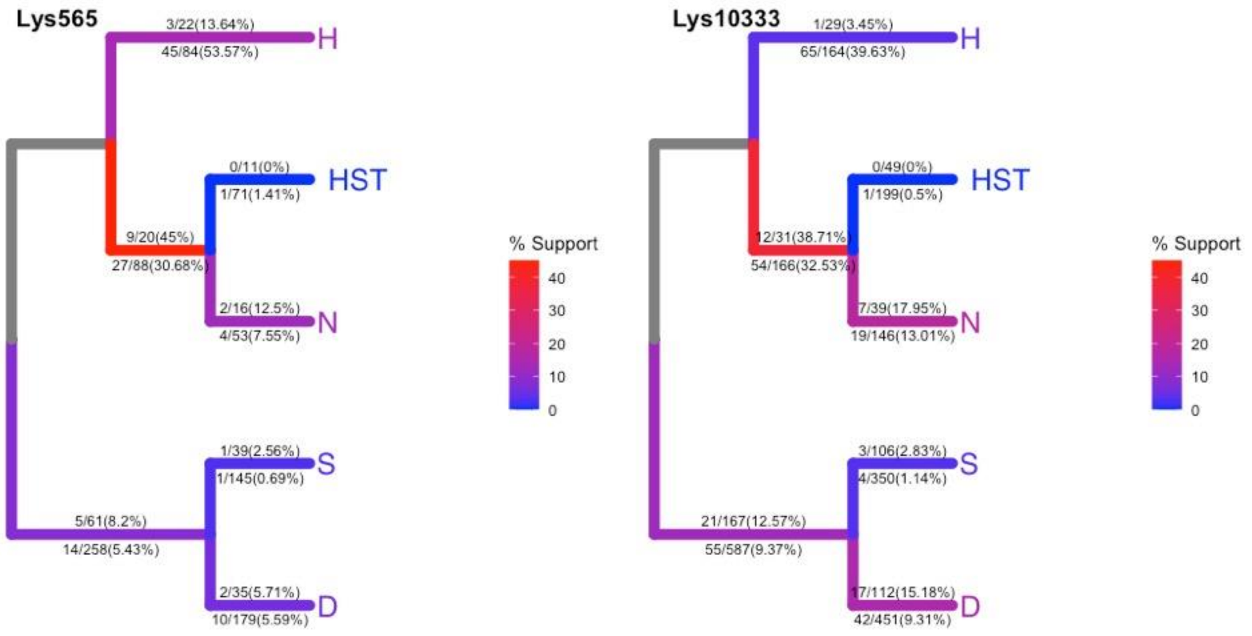

Supplementary Figure 24 | The observed support for different mitochondrial lineages for two sub-samples from sample M76. Each branch is coloured according to the support of the derived state using deaminated fragments only. The number of fragments sharing the derived state and the total number of fragments overlapping lineage-specific diagnostic sites are shown for all (below) and deaminated only fragments (above) each respective branch. H: modern humans; HST: Hohlenstein-Stadel Neanderthal; N: other Neanderthals; S: Sima de los Huesos; D: Denisovans.

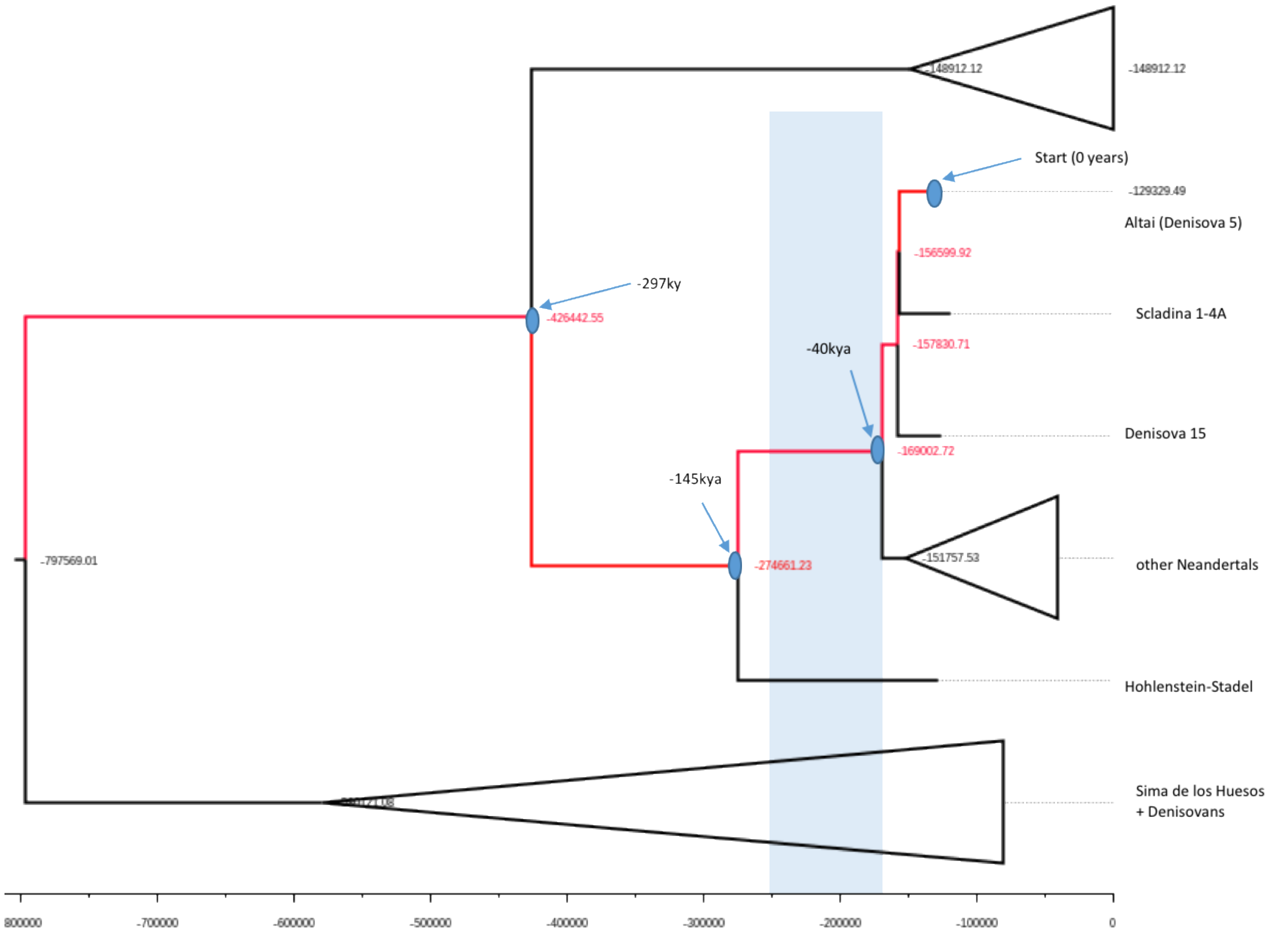

Supplementary Figure 25 | Time points relative to the estimated tip date of the Altai (Denisova 5) Neanderthal. The node dates as determined via BEAST analysis are shown at each node as well as the tip date for the Altai (Denisova 5) Neanderthal. The shaded blue interval represents the time interval of layer 20 in Main Chamber (Jacobs et al., 2019), the layer that SP6731 was sampled from.

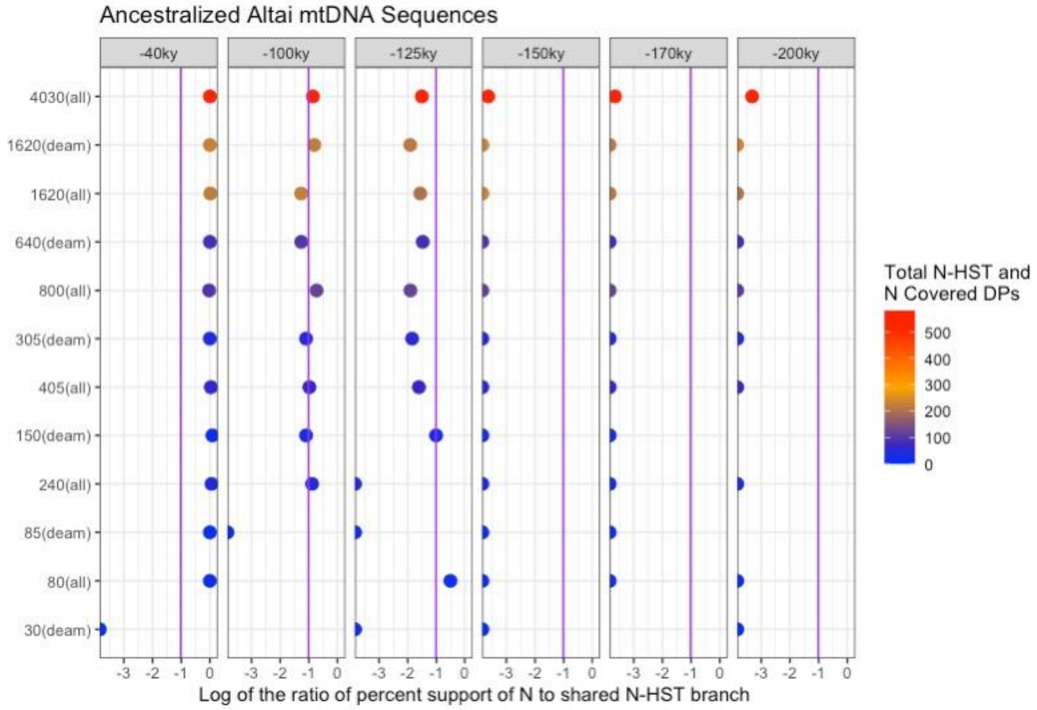

Supplementary Figure 26 | The log ratio between support for the non-HST (N) Neanderthal branch and N-HST branch for simulated sequences from an ancestralized Altai mtDNA genome to different points back in time. Each point is coloured based on the number of fragments covering N-HST and N diagnostic positions (DPs) covered. The purple line represents the log ratio between the outer and inner lineages for the combined fragments from Lys565 and Lys10333 of sample M76. The Y-axis indicates the number of simulated reads (rounded to the nearest 10) used in the analysis after processing and if all or deaminated only fragments were used.

SUPPLEMENTARY SECTION 10: FAUNAL MITOCHONDRIAL DNA ANALYSIS

10.1 Identification of mammalian taxa

Of the 728 samples test, 685 contained traces of ancient DNA pertaining to at least one mammalian family. In our initial analysis, 22 families were identified. Whereas 12 of the identified ancient taxa were present in over 100 libraries, some taxa (n = 10) were detected in less than 10 libraries. These families included Eupleridae (Malagasy carnivores), which are not expected for the region and time period. We visually inspected the sequences assigned to the 10 least abundant families and found that they often concentrated in short, highly conserved parts of the mtDNA genome. This contradicts the expectation that recovered DNA fragments would originate from random parts of the mtDNA genome, and thus be distributed randomly across the reference genome when assigned correctly to a taxon of origin. We therefore excluded assignments to families for each library that were based on alignments to less than 500 bp of the mtDNA reference genome. Using this filter, we removed assignments to the Malagasy carnivores. Summary statistics for each sample and their associated negative controls are provided in Supplementary Data File 3. The relative locations of samples identified as containing ancient mammalian mtDNA, the number of DNA fragments recovered as well as their taxonomic assignments are shown in Extended Data Figures 8 and 9 for Main and East Chamber and Supplementary Figure 27 for South Chamber.

10.2 Comparison to zooarchaeological records

In depth evaluations of the zooarchaeological record have been published throughout the excavations of Denisova Cave for Main and East Chambers [1,66-71]. In comparing the proportion of mtDNA fragments assigned to taxa identified as ancient to the published zooarchaeological records we observed that small mammals (e.g. squirrels (Sciuridae), rodents (Spalacidae), etc.), are poorly represented by the genetic data and are often entirely absent (Extended Data Figure 7a,b). In contrast, all large mammals represented by skeletal remains were detected through genetic analysis, even though there are differences in the proportions of skeletal remains and DNA fragments recovered (Extended Data Figure 7c,d). One library contained ancient DNA from Camelidae, which has not been identified in the skeletal record, but is known to have been present in the region during the middle and late Pleistocene[72]. Comparisons on a per-layer basis, which were performed only for large mammals (excluding hominids) that were observed in more than one library, largely show a good congruence between the genetic and zooarchaeological data. For example, as one moves towards lower layers in the stratigraphy, the concentration of bovids decreases in Main and East Chambers in both types of data whereas the concentration of ursids and canids increases (Extended Data Figure 7c,d). For South Chamber, no published zooarchaeological records are available for comparison with the genetic data (see Extended Data Figure 3).

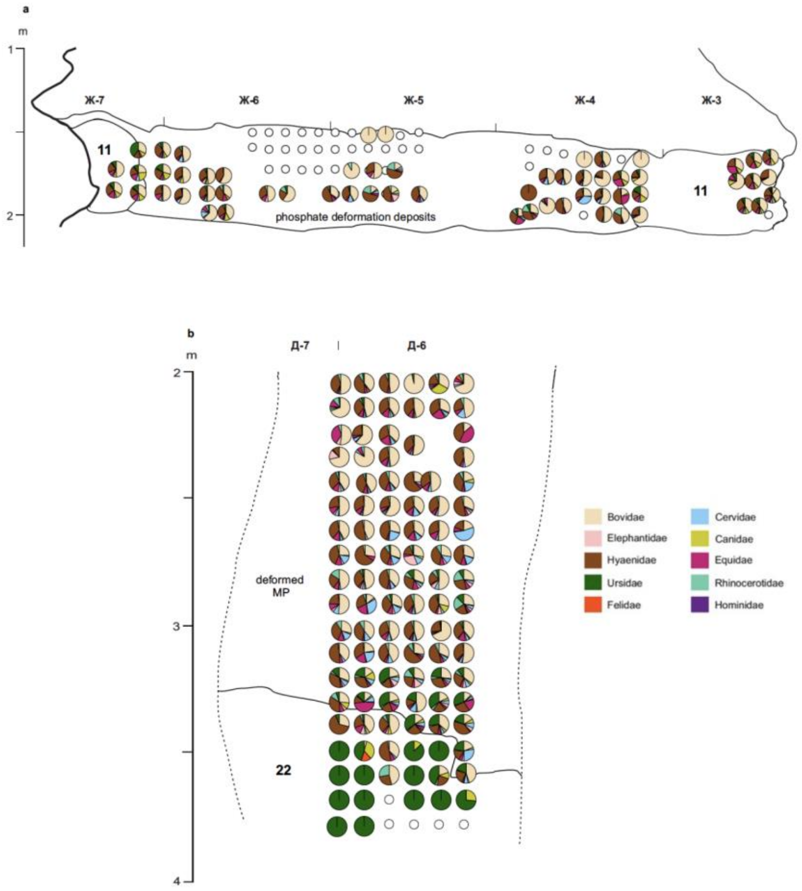

Supplementary Figure 27 | Proportions of ancient mtDNA fragments of large mammals in South Chamber. Pie charts showing proportions of mtDNA fragments assigned to specific mammalian families for each sample; empty circles denote samples that yielded no ancient mtDNA.

SUPPLEMENTARY SECTION 11: FAUNAL IDENTIFICATIONS ON THE SPECIES LEVEL

To investigate potential faunal transitions based on genetic data, it is necessary to assign DNA sequences to specific species. However, analyses beyond the family level rely on the availability of complete mitochondrial genome sequences that cover the genetic diversity of extant and extinct species within a family, as well as the grouping of mtDNA sequences from the relevant species in monophyletic clades. Based on these considerations, we selected for further analysis three mammalian families for which abundant mtDNA sequence data were recovered from multiple layers throughout the stratigraphy of Denisova Cave: ursid (bears), elephantid (elephants), and hyaenids (hyaenas) (Supplementary Figures 28 to 30, respectively).

In an attempt to assign sequences to specific species, we first identified lineage-specific diagnostic positions in the mtDNA genome, following the approach in ref. 22. This was achieved by creating multiple sequence alignments for species within ursids (bears), elephantids (elephants), and hyaenids (hyaenas) using a selection of published complete mitochondrial genomes from each family. At least two different mitochondrial genomes were used per species whenever possible, with additional genomes used for the parts of the species trees in which the remains from Denisova Cave are expected to fall (brown and cave bears for ursids and woolly mammoth for elephantids, all spotted and cave hyaenas for hyaenids)[1,66-69] (Supplementary Data File 3). These genome sequences were then aligned to each other using MAFFT[23]. To visualize the phylogenetic relationships between the genome sequences used for identifying diagnostic positions, we used Mega X[53] to build a Neighbour-Joining tree. Missing positions in any of the genome sequences were removed and evolutionary distances were measured by counting the number of differences[73]. A bootstrap test with 500 replicates was used and the support noted for each branch [74] (Supplementary Figures 31 to 33).

Diagnostic positions were determined by identifying positions where all mtDNA genomes in one group showed a base difference to all other groups. We note that the probe design for the mammalian mitochondrial capture consisted of 5-bp tiling across the mitochondrial genomes of 242 mammals[75]. This could lead to a bias for the increased recovery of fragments from more conserved portions of the mitochondrial genome, which may in turn negatively impact the ability to identify species based on the recovered DNA fragments. To evaluate this possibility, we compared the observed coverage across the mtDNA genome to phyloP conservation scores[39-41]. This was carried out by merging all sequences attributed to ursid or elephantid from all samples were these families were identified as ancient (for a total of 860,488 and 336,506 sequences, respectively) and mapping them to the reference mtDNA genome available for the phyloP value determination (panda bear and African elephant, respectively). No reference with phlyoP values was available for the hyaenid family so this analysis was not performed for these sequences. The correlation between the coverage and relative conservation at each position was then tested with a Spearman's correlation test, resulting in a positive correlation for both families (bears: rho = 0.167, p-value <2.2e-16; elephants: rho = 0.178, p-value <2.2e-16 for all sequences). The sequences for each family were then mapped to mtDNA reference genomes for species identified at Denisova Cave based on skeletal remains (cave bear for bears, NC_011112.1; woolly mammoth for elephants, NC_007596.2; spotted hyaena for hyaenas, NC_020670.1). We then visualized the variation in coverage across positions with histograms, which showed that a small proportion of positions had more than twice the mean observed coverage (Supplementary Figures 34 to 36). To avoid the dominance of a small number of highly covered diagnostic positions in subsequent analyses, the diagnostic positions showing a sequence coverage of more than twice the mean were removed from each set of positions (Supplementary Figures 37 to 39).

Following the identification of diagnostic positions, the number of DNA fragments that support each mitochondrial group were determined separately for each sample containing ancient ursid and elephant DNA (Supplementary Figures 40 and 41) using alignments to the cave bear mtDNA genome (NC_011112.1) for bears, the woolly mammoth mtDNA genome (NC_007596.2) for elephants, and the spotted hyaena

(NC_020670.1) for hyaenas respectively. Support for a specific species was considered significant if at least 3 diagnostic positions were covered by DNA fragments from a given sample and if significantly more than 10% (using 95% confidence intervals from a binomial test) of DNA fragments covering the diagnostic positions shared the state diagnostic for the respective species.

From the 506 samples that were identified as containing ancient bear mtDNA, 413 (82%) were assigned to at least one bear mtDNA group (Supplementary Figure 40 for Main and East Chambers). From these 413 samples 174 contained cave bear mtDNA, 299 contained brown bear mtDNA and sixty of these samples contained both cave and brown bear mtDNA. Based on optical dating of layers [1] the lowest layers of Main Chamber are older than the lowest layers sampled in East Chamber. These layers predominantly contain cave bear DNA. Moving upward in the stratigraphy for Main Chamber and in the lower layers of East Chamber both brown and cave bears were identified. In the upper layers, predominantly brown bears were identified (Extended Data Figure 4). The change to predominantly brown bears when moving up the stratigraphy is consistent with the skeletal records[1], although it should be noted that we do not have mtDNA references for all the bear species identified in Denisova Cave.

Of the 408 samples that were identified as containing ancient elephant mtDNA 367 (90%) were assigned to at least one elephant mtDNA group (Supplementary Figure 41). All 367 of these samples contained significant support for woolly mammoth mtDNA. Fifty-one of these samples also contained significant support for the African and straight-tusked elephant mtDNA clade. Three of these samples also contained evidence for Asian elephant mtDNA, however only unique four diagnostic positions out of the 167 possible diagnostic positions for the Asian elephant mtDNA were covered in these samples. The sequences covering these diagnostic positions were also blasted[76] and showed similarities to both woolly mammoth and Asian elephant. As woolly mammoths and Asian elephants are sister clades it's possible that a lack of published mtDNA genomes of elephants from the relevant time periods and geography precludes the resolution of these samples. Due to the low level of support for the Asian elephant in each of the three samples (<30%) and few diagnostic positions covered, these assignments were removed from further analyses. While woolly mammoth mtDNA was identified throughout the stratigraphy, the presence of African elephant/straight-tusked elephant mtDNA (presumably reflecting straight-tusked elephant) was most concentrated between approximately 150 and 60 ka.

For the hyaenas, all 390 samples that could be assigned significantly to a mtDNA group were assigned to cave and spotted hyaena. To differentiate between the different mtDNA haplogroups within the cave and spotted hyaenas we identified new diagnostic positions for haplogroups A, B, C and D with the striped hyaena as an outgroup (Supplementary Figure 42). We defined diagnostic positions as described above and we removed positions that had above two standard deviations from the mean coverage of all merged ancient hyaena reads. The final number of diagnostic positions used for the identification of different mtDNA groups within the bear, elephant, and hyaena families is shown in Supplementary Figure 43.

With this second set of diagnostic positions for hyaenas, we were able to assign 363 sample of the 490 samples that contained ancient hyaena mtDNA to a mtDNA haplogroup (Supplementary Figure 44). Two hundred and twenty-two of the samples contained significant support for haplogroup A, a clade containing cave and spotted hyaenas from Africa and Eurasia. Twelve samples contained significant support for haplogroup B, a European cave hyaena clade. One hundred and fifty-eight samples contained support for haplogroup D, a clade of Asian cave hyaenas. Eight samples contained support for both haplogroups A and B, twenty-on for both haplogroups A and D, and one sample contained support for both haplogroups B and D. Haplogroup A is dominant throughout much of the stratigraphy, except for the period between 200 and 100 ka when haplogroup D was dominant.

This analysis demonstrates the ability to use sediment DNA to understand transitions between mtDNA groups within different mammalian families. It also emphasizes the importance of having relevant mtDNA reference genomes in the portions of the tree that one is interested in. We are also able to identify that

there is a change in mtDNA groups for bears, elephants and hyaenas between approximately 200 and 100 ka (Extended Data Figure 10).

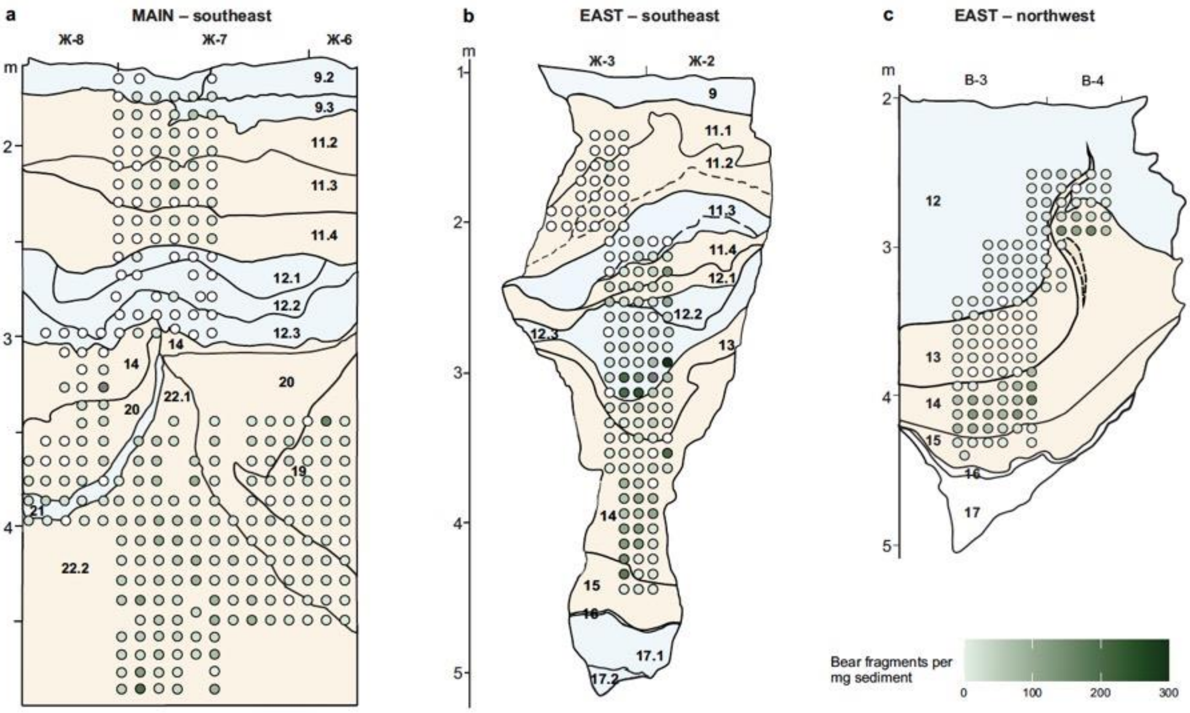

Supplementary Figure 28 | The preservation of ancient bear DNA in the stratigraphy of Main Chamber and southeast and northwest faces of East Chamber. Each filled circle represents a sample identified as containing ancient bear DNA and the shade is scaled according to the number of mtDNA fragments assigned per milligram sediment for each sample. Negative samples are white circles.

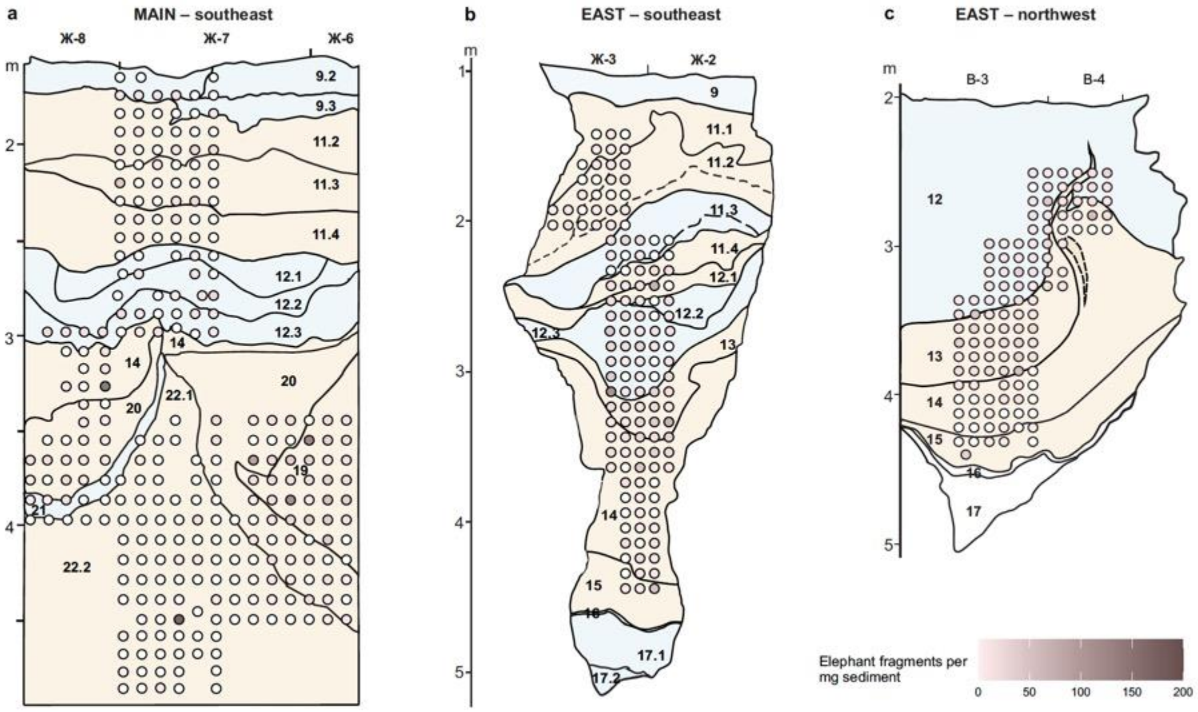

Supplementary Figure 29 | The preservation of ancient elephant DNA in the stratigraphy of Main Chamber and southeast and northwest faces of East Chamber. Each filled circle represents a sample identified as containing ancient elephant DNA and the shade is scaled according to the number of mtDNA fragments assigned per milligram sediment for each sample. Negative samples are white circles.

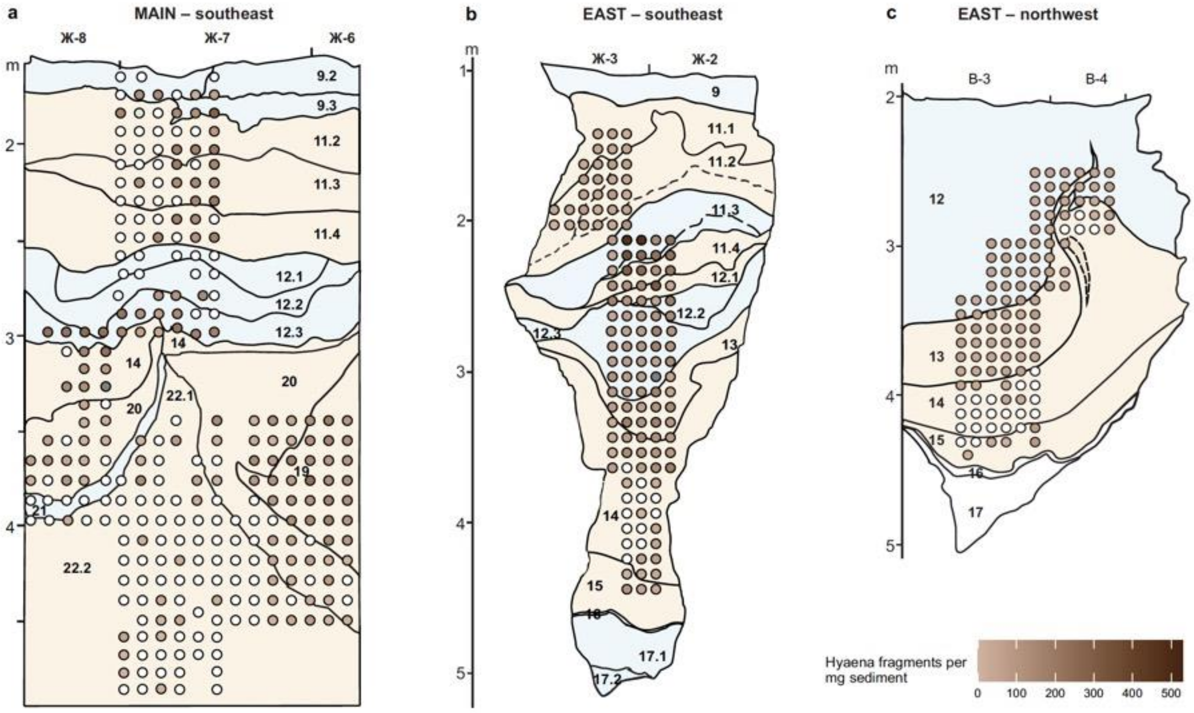

Supplementary Figure 30 | The preservation of ancient hyaena DNA in the stratigraphy of Main Chamber and southeast and northwest faces of East Chamber. Each filled circle represents a sample identified as containing ancient hyaena DNA and the shade is scaled according to the number of mtDNA fragments assigned per milligram sediment for each sample. Negative samples are white circles.

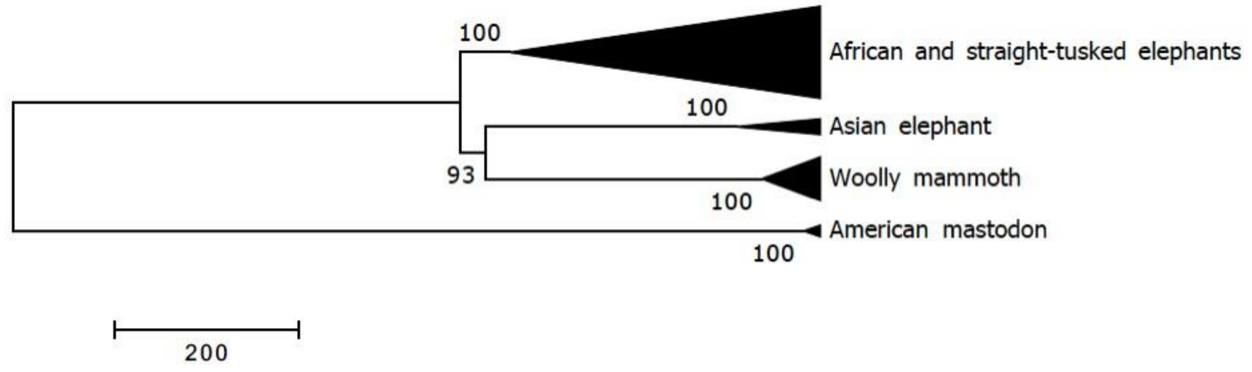

Supplementary Figure 31 | Phylogeny of elephant mitochondrial groups used for determining diagnostic positions. A Neighbour-Joining tree was reconstructed using previously published elephant mitochondrial genomes through MEGA X. Taxa were clustered base on pairwise differences. The support for each node is based on a bootstrap test (500 replicates). Sequences are grouped by how they were for used for determining diagnostic positions.

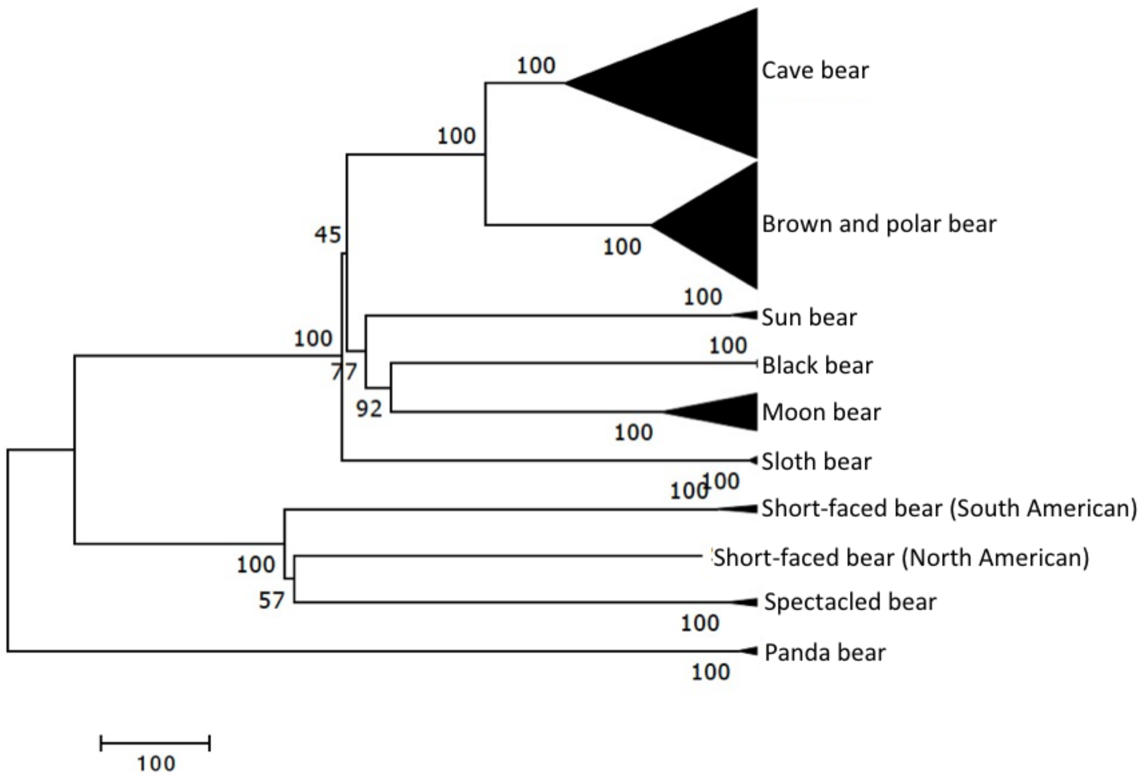

Supplementary Figure 32 | Phylogeny of bear mitochondrial groups used for determining diagnostic positions. A Neighbour-Joining tree reconstructed using previously published bear mitochondrial genomes through MEGA X. Taxa were clustered base on pairwise differences. The support for each node is based on a bootstrap test with 500 replicates. Sequences are grouped by how they were for used for determining diagnostic positions.

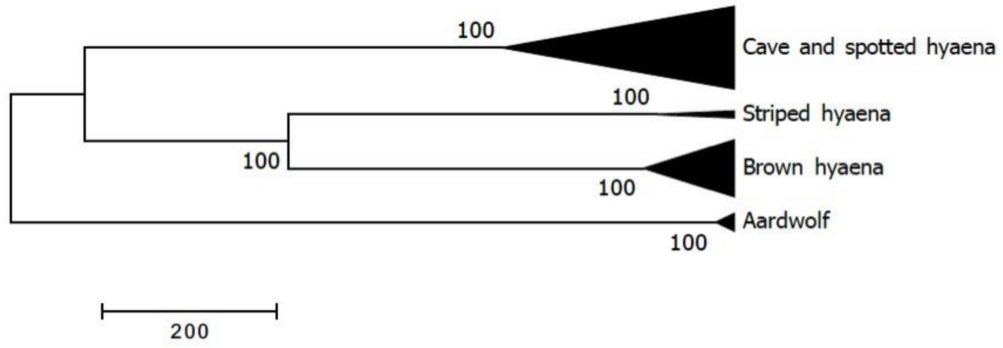

Supplementary Figure 33 | Phylogeny of hyaena mitochondrial groups used for determining diagnostic positions. A Neighbour-Joining tree reconstructed using previously published hyaena mitochondrial genomes through MEGA X. Taxa were clustered base on pairwise differences. The support for each node is based on a bootstrap test with 500 replicates. Sequences are grouped by how they were for used for determining diagnostic positions.

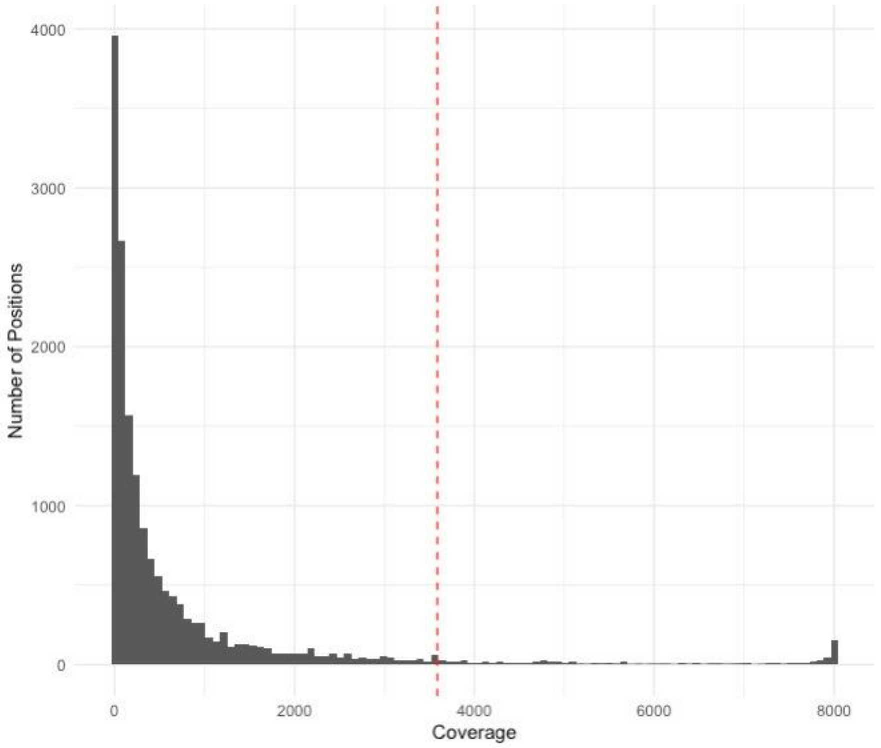

Supplementary Figure 34 | Coverage histogram of the woolly mammoth mtDNA genome using mtDNA fragments from all libraries identified as containing ancient elephantid DNA. The coverage is separated into 100 equal bins for the range of observed coverage (0 to 8008).

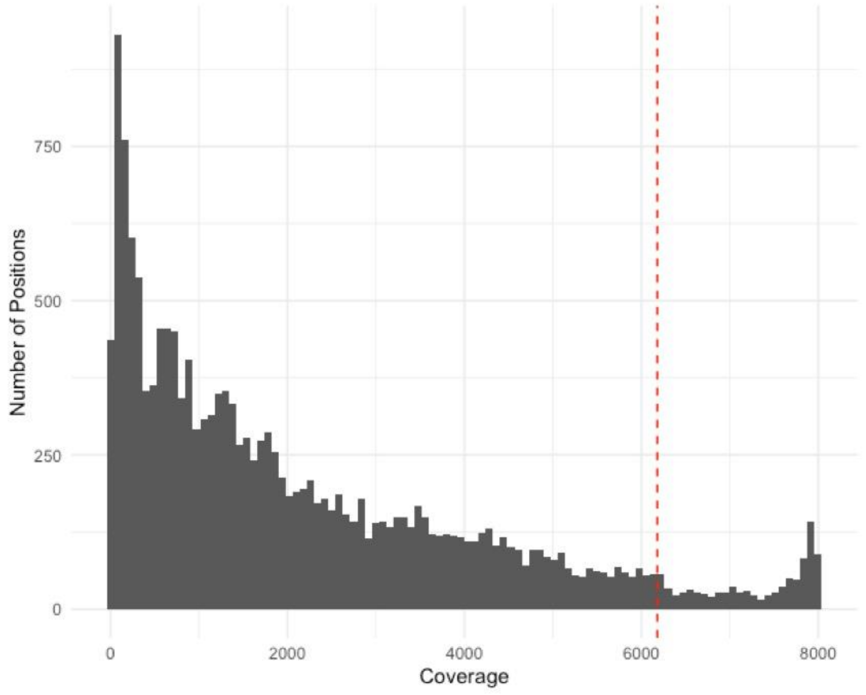

Supplementary Figure 35 | Coverage histogram of the cave bear mtDNA genome using mtDNA fragments from all libraries identified as containing ancient ursid DNA. The coverage is separated into 100 equal bins for the range of observed coverage (0 to 8000). The dotted red line represents two standard deviations from the mean coverage.

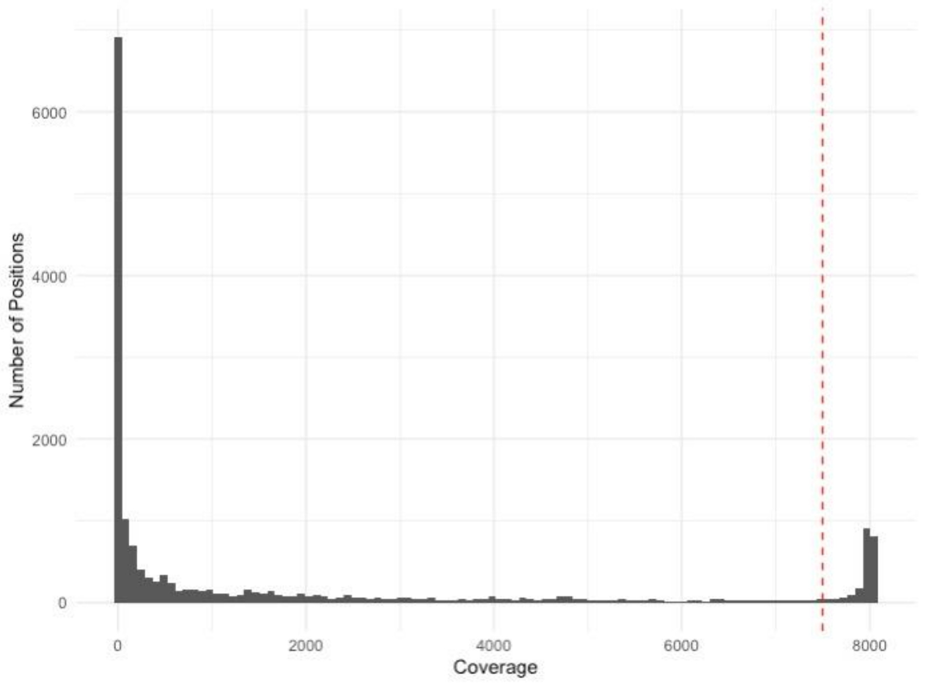

Supplementary Figure 36 | Coverage histogram of the spotted hyaena mtDNA genome using mtDNA fragments from all libraries identified as containing ancient hyaenid DNA. The coverage is separated into 100 equal bins for the range of observed coverage (0 to 8046). The dotted red line represents two standard deviations from the mean coverage.

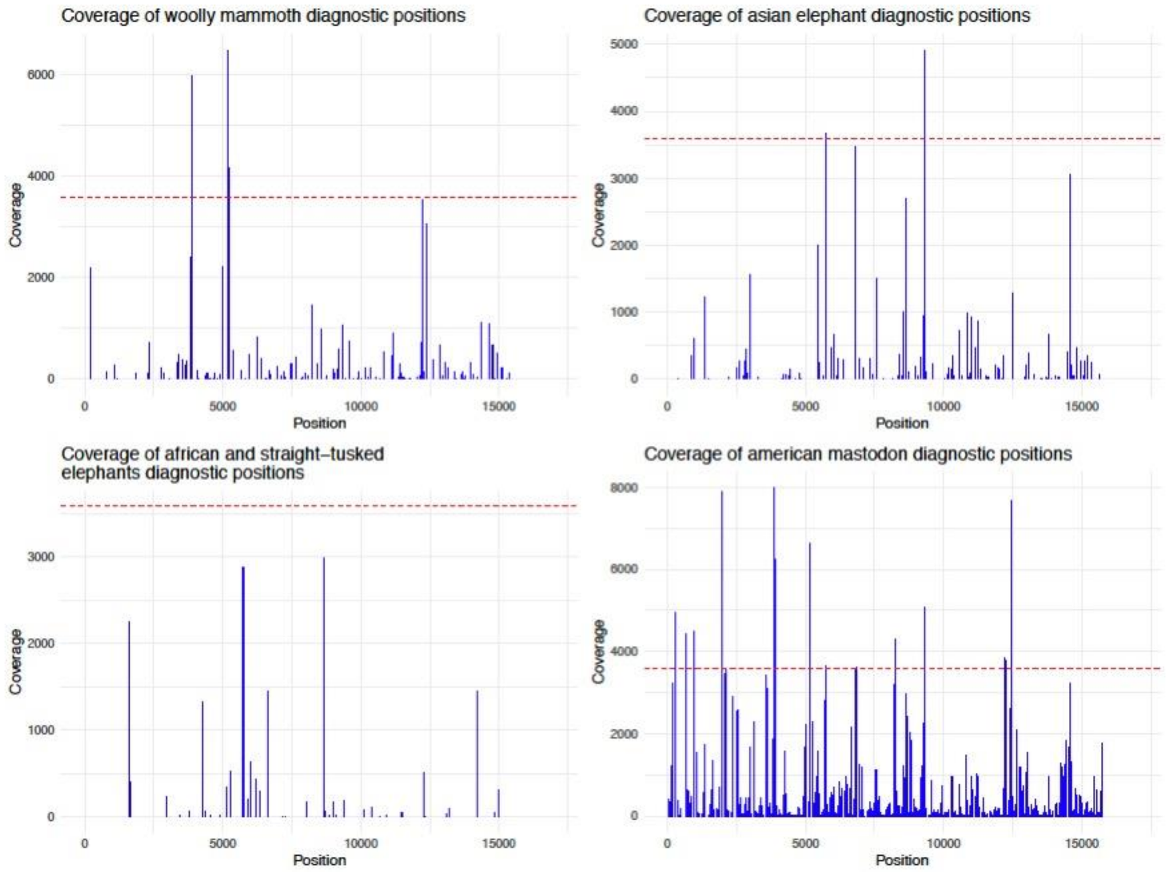

Supplementary Figure 37 | Coverage at positions in the mtDNA genome that are diagnostic for each elephantid mitochondrial group. Coverage plots are based on the alignment of all mtDNA fragments from samples identified as containing ancient elephant DNA to the woolly mammoth mtDNA genome. The dotted red line represents two standard deviations from the mean coverage, which was used as a cut-off for removing positions from further analysis.

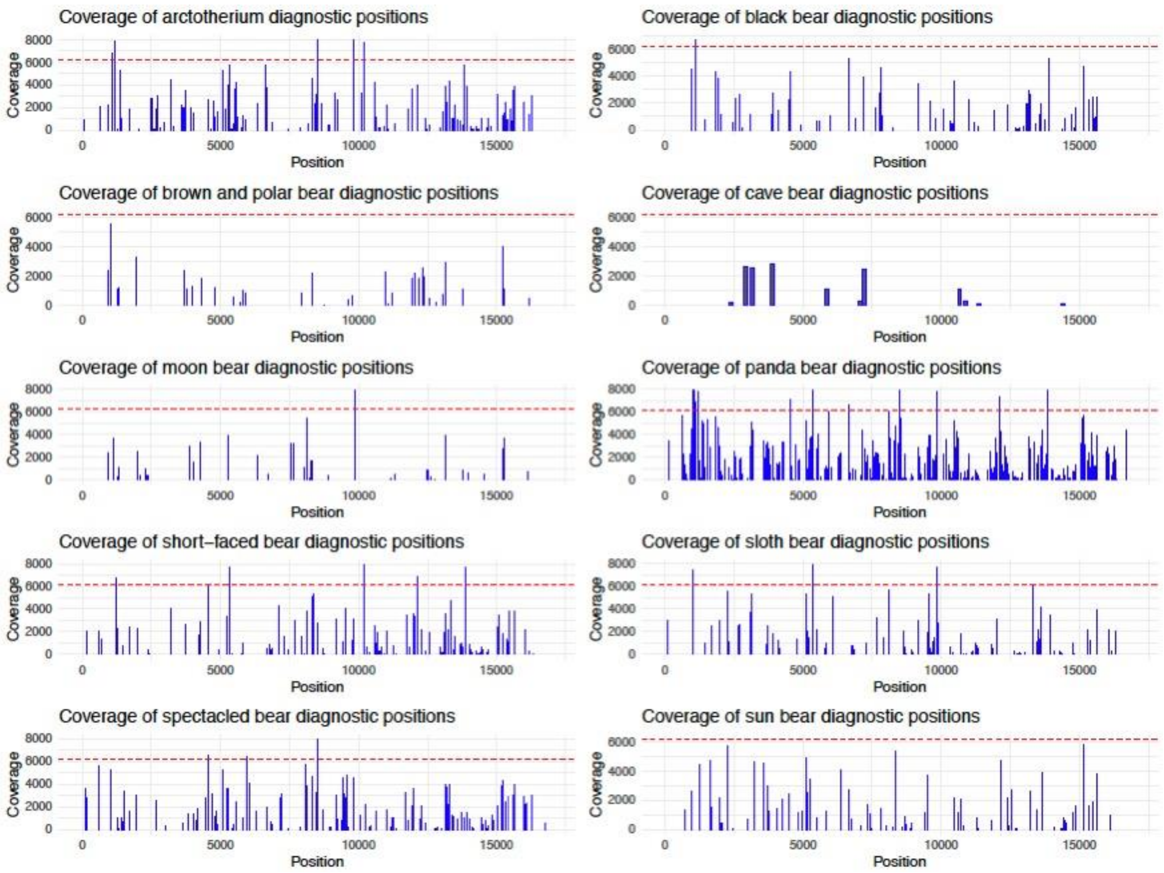

Supplementary Figure 38 | Coverage at positions in the mtDNA that are diagnostic for each bear mitochondrial group. Coverage plots are based on the alignment of all mtDNA fragments from samples identified as containing ancient ursid DNA to the cave bear mtDNA genome. The dotted red line represents two standard deviations from the mean coverage, which was used as a cut-off for removing positions from further analysis.

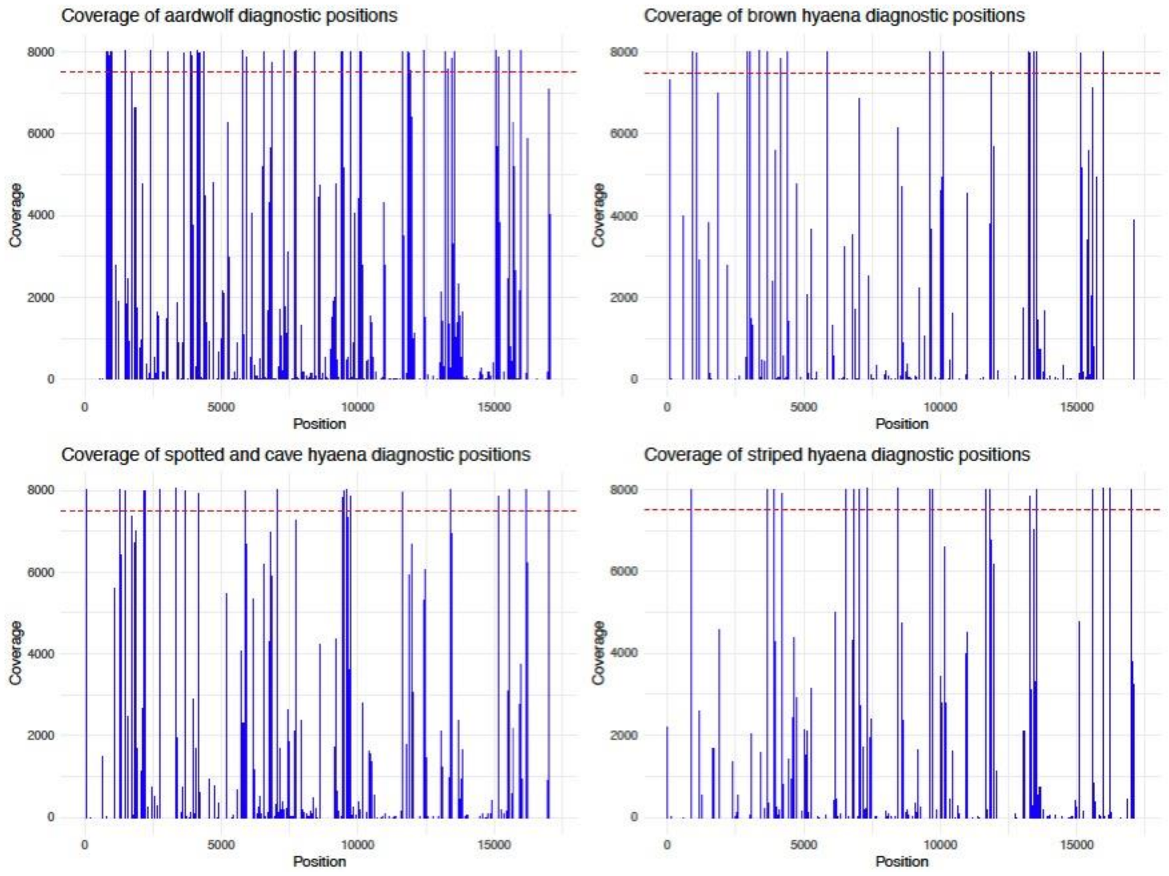

Supplementary Figure 39 | Coverage at positions in the mtDNA that are diagnostic for each hyaena mitochondrial group. Coverage plots are based on the alignment of all mtDNA fragments from samples identified as containing ancient hyaenid DNA to the spotted hyaena mtDNA genome. The dotted red line represents two standard deviations from the mean coverage, which was used as a cut-off for removing positions from further analysis.

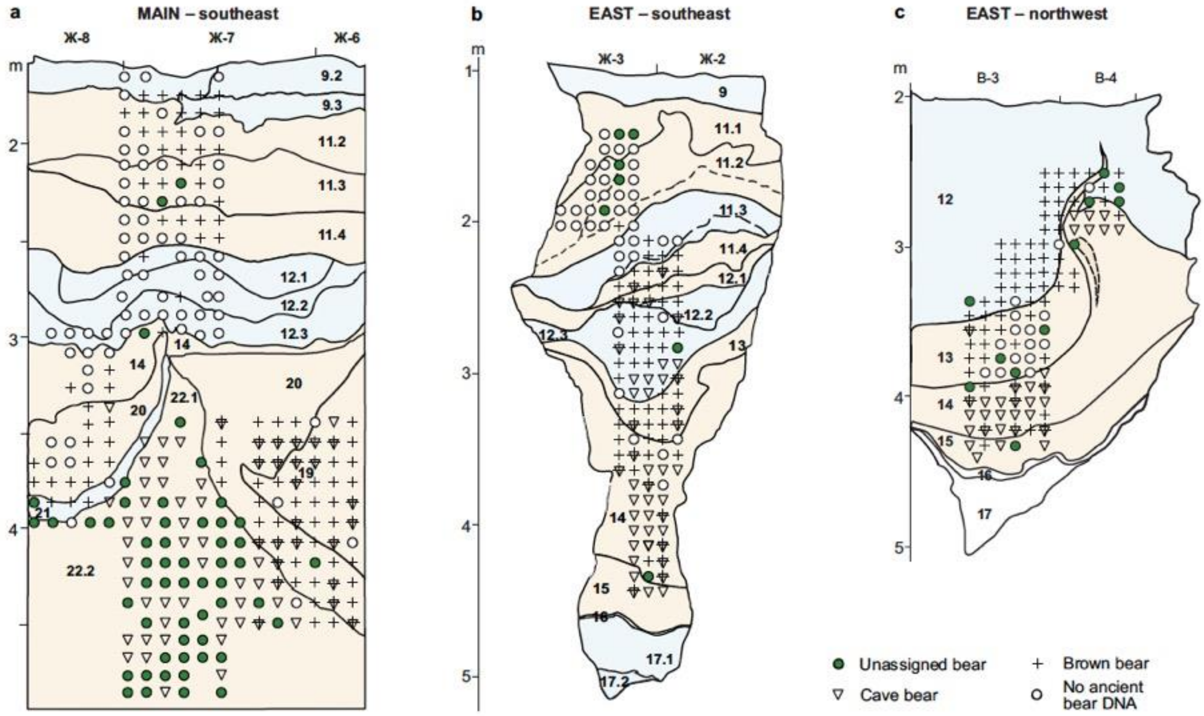

Supplementary Figure 40 | Identification of different bear mtDNA groups in the stratigraphy of Main Chamber and southeast and northwest faces of East Chamber. Samples with significant support for brown and cave bear are shown with crosses and triangles, respectively. Samples containing ancient bear DNA, but that were not assigned to a specific lineage are shown in green circles, negative samples are shown in white circles. No samples had significant support for other bear mtDNA groups. The layers coloured in blue represent relatively cold periods and in orange are relatively warm periods.

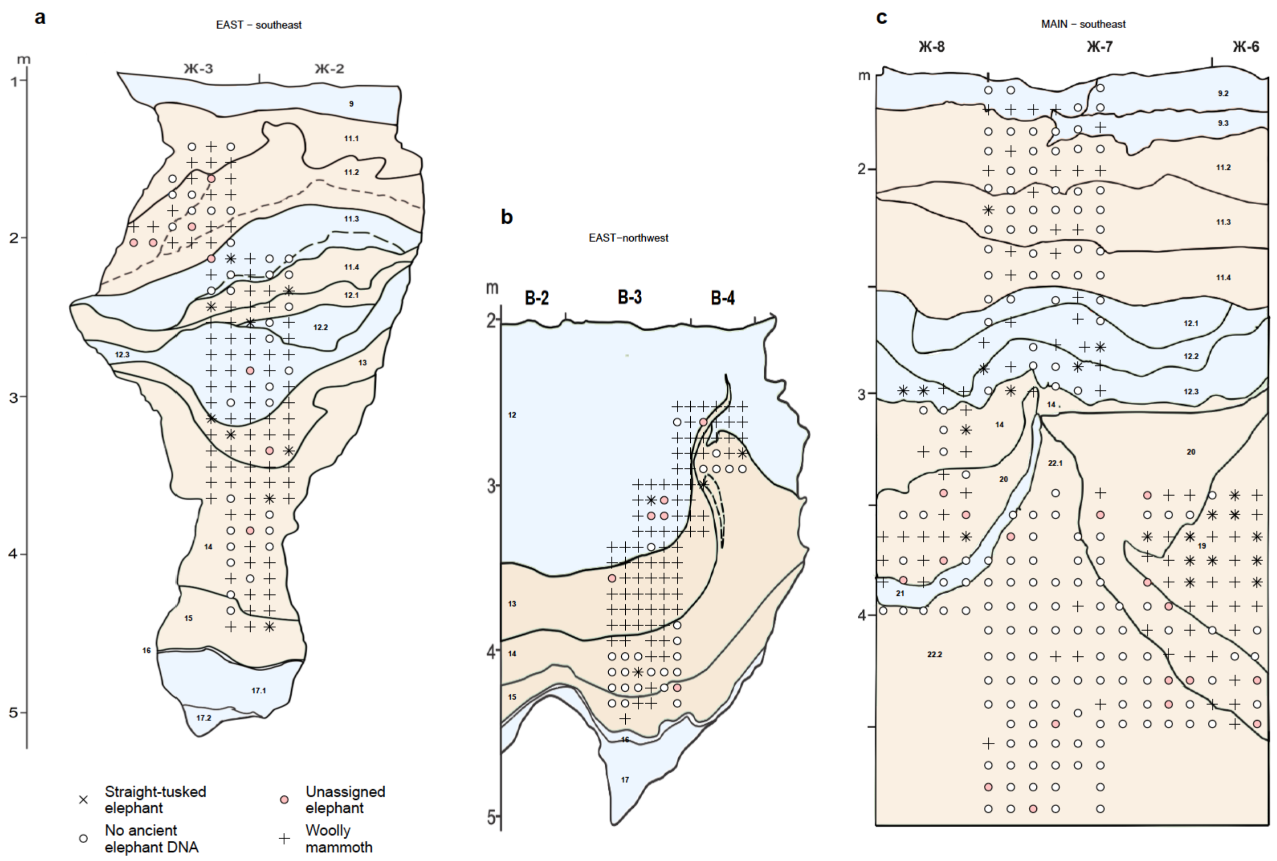

Supplementary Figure 41 | Identification of different elephant mtDNA groups in the stratigraphy of Main Chamber and southeast and northwest faces of East Chamber. Samples with significant support for straight-tusked elephant, Asian elephant and woolly mammoth are shown with x's, triangles and crosses, respectively. Samples containing ancient elephant DNA, but that were not assigned to a specific lineage are shown in pink circles, negative samples are shown in white circles. No samples had significant support for other elephant mtDNA groups. The layers coloured in blue represent relatively cold periods and in orange are relatively warm periods.

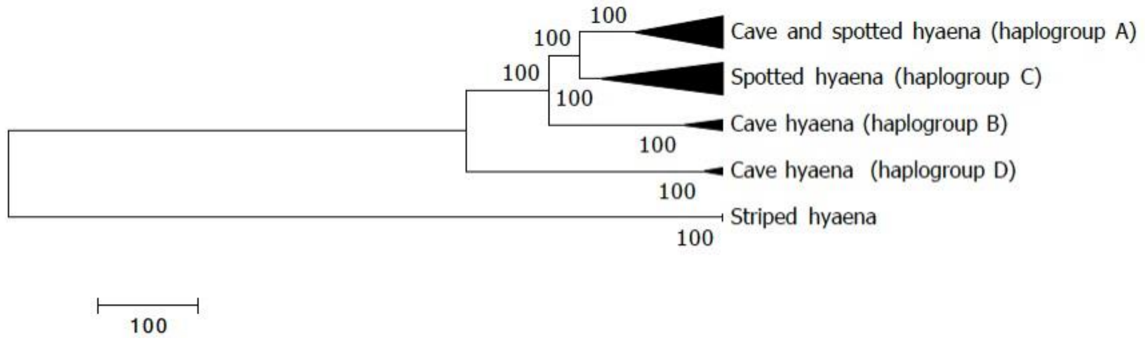

Supplementary Figure 42 | Phylogeny of cave and spotted hyaena mitochondrial haplogroups used for determining diagnostic positions. A Neighbour-Joining tree reconstructed using previously published hyaena mitochondrial genomes through MEGA X. Taxa were clustered base on pairwise differences. The support for each node is based on a bootstrap test with 500 replicates. Sequences are grouped by how they were for used for determining diagnostic positions.

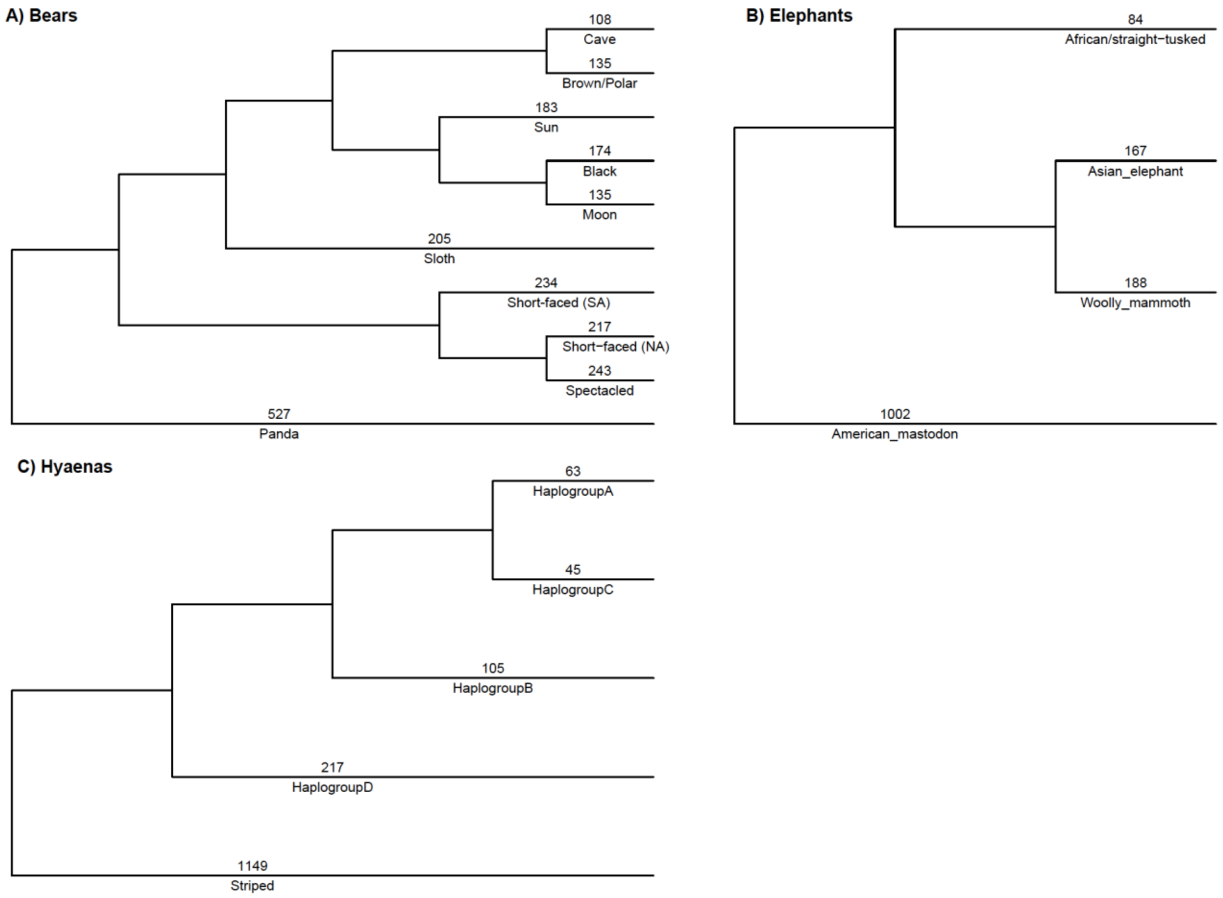

Supplementary Figure 43 | Number of identified diagnostic positions for mtDNA groups in the elephant, hyaena and bear families. The number of diagnostic positions is above and the relevant mtDNA group is below each respective branch. For the short-faced bears SA refers to South American and NA refers to North American.

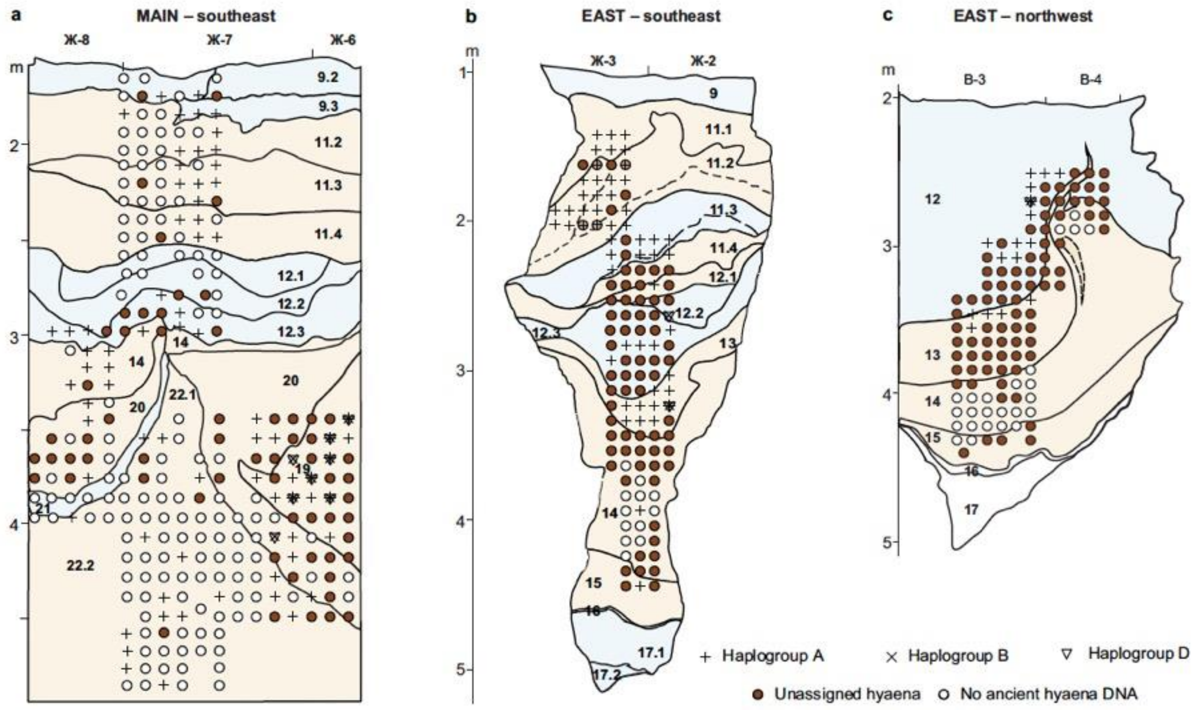

Supplementary Figure 44| Identification of different hyaena mtDNA groups in the stratigraphy of Main Chamber and southeast and northwest faces of East Chamber. Samples with significant support for haplogroups A, B and C are shown with crosses, x's, and triangles, respectively. Samples containing ancient hyaena DNA, but that were not assigned to a specific lineage are shown in brown circles, negative samples are shown in white circles. No samples had significant support for other hyaena mtDNA groups. The layers coloured in blue represent relatively cold periods and those in orange represent relatively warm periods.

REFERENCES

1 Jacobs, Z. *et al.* Timing of archaic hominin occupation of Denisova Cave in southern Siberia. *Nature* **565**, 594-599, doi:10.1038/s41586-018-0843-2 (2019).

2 Douka, K. *et al.* Age estimates for hominin fossils and the onset of the Upper Palaeolithic at Denisova Cave. *Nature* **565**, 640-644, doi:10.1038/s41586-018-0870-z (2019).

3 Morley, M. W. *et al.* Hominin and animal activities in the microstratigraphic record from Denisova Cave (Altai Mountains, Russia). *Sci Rep* **9**, 13785, doi:10.1038/s41598-019-49930-3 (2019).

4 Slon, V. *et al.* Neandertal and Denisovan DNA from Pleistocene sediments. *Science* **356**, 605-608, doi:10.1126/science.aam9695 (2017).

5 Slon, V. *et al.* A fourth Denisovan individual. *Sci Adv* **3**, e1700186, doi:10.1126/sciadv.1700186 (2017).

6 Lorenz, M. G. & Wackernagel, W. Adsorption of DNA to Sand and Variable Degradation Rates of Adsorbed DNA. *Appl Environ Microb* **53**, 2948-2952, doi:Doi 10.1128/Aem.53.12.2948-2952.1987 (1987).

7 Ogram, A., Sayler, G. S., Gustin, D. & Lewis, R. J. DNA Adsorption to Soils and Sediments. *Environmental Science & Technology* **22**, 982-984, doi:DOI 10.1021/es00173a020 (1988).

8 Solomon, S. *et al.* High-quality metagenomic DNA from marine sediment samples for genomic studies through a preprocessing approach. *3 Biotech* **6**, 160, doi:10.1007/s13205-016-0482-y (2016).

9 Kirkpatrick, J. B., Walsh, E. A. & D'Hondt, S. Fossil DNA persistence and decay in marine sediment over hundred-thousand-year to million-year time scales. *Geology* **44**, 615-618, doi:10.1130/g37933.1 (2016).

10 Boere, A. C., Rijpstra, W. I., De Lange, G. J., Sinninghe Damste, J. S. & Coolen, M. J. Preservation potential of ancient plankton DNA in Pleistocene marine sediments. *Geobiology* **9**, 377-393, doi:10.1111/j.1472-4669.2011.00290.x (2011).

11 Kistler, L., Ware, R., Smith, O., Collins, M. & Allaby, R. G. A new model for ancient DNA decay based on paleogenomic meta-analysis. *Nucleic Acids Res* **45**, 6310-6320, doi:10.1093/nar/gkx361 (2017).

12 Weiss, C. L. *et al.* Temporal patterns of damage and decay kinetics of DNA retrieved from plant herbarium specimens. *R Soc Open Sci* **3**, 160239, doi:10.1098/rsos.160239 (2016).

13 Sawyer, S., Krause, J., Guschanski, K., Savolainen, V. & Paabo, S. Temporal patterns of nucleotide misincorporations and DNA fragmentation in ancient DNA. *PLoS One* **7**, e34131, doi:10.1371/journal.pone.0034131 (2012).

14 Allentoft, M. E. *et al.* The half-life of DNA in bone: measuring decay kinetics in 158 dated fossils. *Proc Biol Sci* **279**, 4724-4733, doi:10.1098/rspb.2012.1745 (2012).

15 Briggs, A. W. *et al.* Patterns of damage in genomic DNA sequences from a Neandertal. *Proc Natl Acad Sci U S A* **104**, 14616-14621, doi:10.1073/pnas.0704665104 (2007).

16 Gansauge, M. T. *et al.* Single-stranded DNA library preparation from highly degraded DNA using T4 DNA ligase. *Nucleic Acids Res* **45**, e79, doi:10.1093/nar/gkx033 (2017).

17 Glocke, I. & Meyer, M. Extending the spectrum of DNA sequences retrieved from ancient bones and teeth. *Genome Res* **27**, 1230-1237, doi:10.1101/gr.219675.116 (2017).

18 Greer, S. & Zamenhof, S. Studies on depurination of DNA by heat. *J Mol Biol* **4**, 123-141, doi:10.1016/s0022-2836(62)80046-1 (1962).

19 Giguet-Covex, C. *et al.* New insights on lake sediment DNA from the catchment: importance of taphonomic and analytical issues on the record quality. *Sci Rep* **9**, 14676, doi:10.1038/s41598-019-50339-1 (2019).

20 Shunkov, M. V. The phosphates of Pleistocene–Holocene sediments of the Eastern Gallery of Denisova Cave. *Dokl. Earth Sci.* **478**, 46-50, doi:10.1134/S1028334X18010270 (2018).

21 Benjamini, Y. & Yekutieli, D. The control of the false discovery rate in multiple testing under dependency. *Annals of Statistics* **29**, 1165-1188 (2001).

22 Meyer, M. *et al.* Nuclear DNA sequences from the Middle Pleistocene Sima de los Huesos hominins. *Nature* **531**, 504-507, doi:10.1038/nature17405 (2016).

23 Katoh, K. & Standley, D. M. MAFFT multiple sequence alignment software version 7: improvements in performance and usability. *Mol Biol Evol* **30**, 772-780, doi:10.1093/molbev/mst010 (2013).

24 Hajdinjak, M. *et al.* Reconstructing the genetic history of late Neanderthals. *Nature* **555**, 652-656, doi:10.1038/nature26151 (2018).
25 Prufer, K. *et al.* The complete genome sequence of a Neanderthal from the Altai Mountains. *Nature* **505**, 43-49, doi:10.1038/nature12886 (2014).
26 Green, R. E. *et al.* A complete Neandertal mitochondrial genome sequence determined by high-throughput sequencing. *Cell* **134**, 416-426, doi:10.1016/j.cell.2008.06.021 (2008).
27 Rougier, H. *et al.* Neandertal cannibalism and Neandertal bones used as tools in Northern Europe. *Sci Rep* **6**, 29005, doi:10.1038/srep29005 (2016).
28 Briggs, A. W. *et al.* Targeted retrieval and analysis of five Neandertal mtDNA genomes. *Science* **325**, 318-321, doi:10.1126/science.1174462 (2009).
29 Skoglund, P. *et al.* Separating endogenous ancient DNA from modern day contamination in a Siberian Neandertal. *Proc Natl Acad Sci U S A* **111**, 2229-2234, doi:10.1073/pnas.1318934111 (2014).
30 Brown, S. *et al.* Identification of a new hominin bone from Denisova Cave, Siberia using collagen fingerprinting and mitochondrial DNA analysis. *Sci Rep* **6**, 23559, doi:10.1038/srep23559 (2016).
31 Gansauge, M. T. & Meyer, M. Selective enrichment of damaged DNA molecules for ancient genome sequencing. *Genome Res* **24**, 1543-1549, doi:10.1101/gr.174201.114 (2014).
32 Peyrégne, S. *et al*. Nuclear DNA from two early Neandertals reveals 80,000 years of genetic continuity in Europe. *Sci Adv* **5**, eaaw5873, doi:10.1126/sciadv.aaw5873 (2019).
33 Meyer, M. *et al.* A mitochondrial genome sequence of a hominin from Sima de los Huesos. *Nature* **505**, 403-406, doi:10.1038/nature12788 (2014).
34 Krause, J. *et al.* The complete mitochondrial DNA genome of an unknown hominin from southern Siberia. *Nature* **464**, 894-897, doi:10.1038/nature08976 (2010).
35 Reich, D. *et al.* Genetic history of an archaic hominin group from Denisova Cave in Siberia. *Nature* **468**, 1053-1060, doi:10.1038/nature09710 (2010).
36 Sawyer, S. *et al.* Nuclear and mitochondrial DNA sequences from two Denisovan individuals. *Proc Natl Acad Sci U S A* **112**, 15696-15700, doi:10.1073/pnas.1519905112 (2015).
37 U'Ren, J. M., Lutzoni, F., Miadlikowska, J., Laetsch, A. D. & Arnold, A. E. Host and geographic structure of endophytic and endolichenic fungi at a continental scale. *Am J Bot* **99**, 898-914, doi:10.3732/ajb.1100459 (2012).
38 Andrews, R. M. *et al.* Reanalysis and revision of the Cambridge reference sequence for human mitochondrial DNA. *Nat Genet* **23**, 147, doi:10.1038/13779 (1999).
39 Pollard, K. S., Hubisz, M. J., Rosenbloom, K. R. & Siepel, A. Detection of nonneutral substitution rates on mammalian phylogenies. *Genome Res* **20**, 110-121, doi:10.1101/gr.097857.109 (2010).
40 Siepel A., P. K. S., Haussler D. in *Research in Computational Molecular Biology* Vol. 3909 (ed Istrail S. Guerra C., Pevzner P.A., Waterman M.) (Springer, Berlin, Heidelberg, 2006).
41 Cooper, G. M. *et al.* Distribution and intensity of constraint in mammalian genomic sequence. *Genome Res* **15**, 901-913, doi:10.1101/gr.3577405 (2005).
42 Weissensteiner, H. *et al.* HaploGrep 2: mitochondrial haplogroup classification in the era of high-throughput sequencing. *Nucleic Acids Res* **44**, W58-63, doi:10.1093/nar/gkw233 (2016).
43 Fu, Q. *et al.* Genome sequence of a 45,000-year-old modern human from western Siberia. *Nature* **514**, 445-449, doi:10.1038/nature13810 (2014).
44 Fu, Q. *et al.* DNA analysis of an early modern human from Tianyuan Cave, China. *Proc Natl Acad Sci U S A* **110**, 2223-2227, doi:10.1073/pnas.1221359110 (2013).
45 Fu, Q. *et al.* A revised timescale for human evolution based on ancient mitochondrial genomes. *Curr Biol* **23**, 553-559, doi:10.1016/j.cub.2013.02.044 (2013).
46 Ermini, L. *et al.* Complete mitochondrial genome sequence of the Tyrolean Iceman. *Curr Biol* **18**, 1687-1693, doi:10.1016/j.cub.2008.09.028 (2008).
47 Krause, J. *et al.* A complete mtDNA genome of an early modern human from Kostenki, Russia. *Curr Biol* **20**, 231-236, doi:10.1016/j.cub.2009.11.068 (2010).
48 Marom, A., McCullagh, J. S., Higham, T. F., Sinitsyn, A. A. & Hedges, R. E. Single amino acid radiocarbon dating of Upper Paleolithic modern humans. *Proc Natl Acad Sci U S A* **109**, 6878-6881, doi:10.1073/pnas.1116328109 (2012).

49 Gilbert, M. T. *et al.* Paleo-Eskimo mtDNA genome reveals matrilineal discontinuity in Greenland. *Science* **320**, 1787-1789, doi:10.1126/science.1159750 (2008).
50 Rasmussen, M. *et al.* Ancient human genome sequence of an extinct Palaeo-Eskimo. *Nature* **463**, 757-762, doi:10.1038/nature08835 (2010).
51 Shang, H., Tong, H., Zhang, S., Chen, F. & Trinkaus, E. An early modern human from Tianyuan Cave, Zhoukoudian, China. *Proc Natl Acad Sci U S A* **104**, 6573-6578, doi:10.1073/pnas.0702169104 (2007).
52 Horai, S., Hayasaka, K., Kondo, R., Tsugane, K. & Takahata, N. Recent African Origin of Modern Humans Revealed by Complete Sequences of Hominoid Mitochondrial Dnas. *P Natl Acad Sci USA* **92**, 532-536, doi:DOI 10.1073/pnas.92.2.532 (1995).
53 Kumar, S., Stecher, G., Li, M., Knyaz, C. & Tamura, K. MEGA X: Molecular Evolutionary Genetics Analysis across Computing Platforms. *Mol Biol Evol* **35**, 1547-1549, doi:10.1093/molbev/msy096 (2018).
54 Bouckaert, R. *et al.* BEAST 2: A Software Platform for Bayesian Evolutionary Analysis. *Plos Computational Biology* **10**, doi:ARTN e1003537 10.1371/journal.pcbi.1003537 (2014).
55 Leache, A. D., Fujita, M. K., Minin, V. N. & Bouckaert, R. R. Species Delimitation using Genome-Wide SNP Data. *Syst Biol* **63**, 534-542, doi:10.1093/sysbio/syu018 (2014).
56 Kass, R. E. & Raftery, A. E. Bayes Factors. *J Am Stat Assoc* **90**, 773-795, doi:10.1080/01621459.1995.10476572 (1995).
57 Baele, G. *et al.* Improving the accuracy of demographic and molecular clock model comparison while accommodating phylogenetic uncertainty. *Mol Biol Evol* **29**, 2157-2167, doi:10.1093/molbev/mss084 (2012).
58 Tamura, K. & Nei, M. Estimation of the number of nucleotide substitutions in the control region of mitochondrial DNA in humans and chimpanzees. *Mol Biol Evol* **10**, 512-526, doi:10.1093/oxfordjournals.molbev.a040023 (1993).
59 Posth, C. *et al.* Deeply divergent archaic mitochondrial genome provides lower time boundary for African gene flow into Neanderthals. *Nat Commun* **8**, 16046, doi:10.1038/ncomms16046 (2017).
60 Meyer, M. *et al.* A high-coverage genome sequence from an archaic Denisovan individual. *Science* **338**, 222-226, doi:10.1126/science.1224344 (2012).
61 Prüfer, Kay, et al. A high-coverage Neandertal genome from Vindija Cave in Croatia. *Science* **358**, 655-658 (2017).
62 Bray, N. L., Pimentel, H., Melsted, P. & Pachter, L. Near-optimal probabilistic RNA-seq quantification. *Nat Biotechnol* **34**, 525-527, doi:10.1038/nbt.3519 (2016).
63 Sagulenko, P., Puller, V. & Neher, R. A. TreeTime: Maximum-likelihood phylodynamic analysis. *Virus Evol* **4**, vex042, doi:10.1093/ve/vex042 (2018).
64 Vernot, B. *et al.* Unearthing Neanderthal population history using nuclear and mitochondrial DNA from cave sediments. *Science* **372**, eabf1667, doi:10.1126/science.eabf1667 (2021).
65 Benjamini, Y. & Hochberg, Y. Controlling the false discovery rate: a practical and powerful approach to multiple testing. *Journal of the Royal Statistical Society* **57**, 289-300 (1995).
66 Agadjanian, A. K. & Serdyuk, N. V. History of mammalian communities and paleogeography of Altai Mountains in the Paleolithic. *Paleontol J* **39**, S645-S821 (2005).
67 Vasiliev, S. K., Shunkov, M. V. & Tsybankov, A. A. in *Problems of Archaeology, Ethnography and Anthropology of Siberia and Neighbouring Territories* Vol. 14 (eds Derevianko, A. P. et al.) 26-31 (Institute of Archaeology and Ethnography, Siberian Branch of the Russian Academy of Sciences, Novosibirsk, 2008).
68 Vasiliev, S. K., Shunkov, M. V. & Tsybankov, A. A. in *Problems of Archaeology, Ethnography and Anthropology of Siberia and Neighbouring Territories* Vol. 16 (eds Derevianko, A. P. et al.) 28-32 (Institute of Archaeology and Ethnography, Siberian Branch of the Russian Academy of Sciences, Novosibirsk, 2010).
69 Vasiliev, S. K., Shunkov, M. V. & Tsybankov, A. A. in *Problems of Archaeology, Ethnography and Anthropology of Siberia and Neighbouring Territories* Vol. 19 (eds Derevianko, A. P. et al.) 32-38 (Institute of Archaeology and Ethnography, Siberian Branch of the Russian Academy of Sciences, Novosibirsk, 2013).

70 Agadjanian, A. K., Shunkov, M. V. & Kozlikin, M. B. in *Problems of Archaeology, Ethnography and Anthropology of Siberia and Neighbouring Territories* Vol. 23 (eds Derevianko, A. P. et al.) 7-10 (Institute of Archaeology and Ethnography, Siberian Branch of the Russian Academy of Sciences, Novosibirsk, 2017).
71 Vasiliev, S. K., Shunkov, M. V. & Kozlikin, M. B. in *Problems of Archaeology, Ethnography and Anthropology of Siberia and Neighbouring Territories* Vol. 23 (eds Derevianko, A. P. et al.) 60-64 (Institute of Archaeology and Ethnography, Siberian Branch of the Russian Academy of Sciences, Novosibirsk, 2017).
72 Titov, V. V. Habitat conditions for Camelus knoblochi and factors in its extinction. *Quaternary International* **179**, 120-125, doi:10.1016/j.quaint.2007.10.022 (2008).
73 Nei, M. & Kumar, S. *Molecular evolution and phylogenetics*. (Oxford University Press, 2000).
74 Felsenstein, J. Confidence Limits on Phylogenies: An Approach Using the Bootstrap. *Evolution* **39**, 783-791, doi:10.1111/j.1558-5646.1985.tb00420.x (1985).
75 Slon, V. *et al.* Mammalian mitochondrial capture, a tool for rapid screening of DNA preservation in faunal and undiagnostic remains, and its application to Middle Pleistocene specimens from Qesem Cave (Israel). *Quaternary International* **398**, 210-218, doi:10.1016/j.quaint.2015.03.039 (2016).
76 Altschul, S. F., Gish, W., Miller, W., Myers, E. W. & Lipman, D. J. Basic local alignment search tool. *J Mol Biol* **215**, 403-410, doi:10.1016/S0022-2836(05)80360-2 (1990).
