# Supplementary material for: Pleistocene sediment DNA reveals hominin and faunal turnovers at Denisova Cave
Source: Nature. 2021 Jun 23;595(7867):399–403. doi: 10.1038/s41586-021-03675-0 (PMC8277575; doi:10.1038/s41586-021-03675-0)
Supplement: Supplementary file 2 — Reporting Summary [file 41586_2021_3675_MOESM2_ESM.pdf]

nature research

Corresponding author(s): Elena I. Zavala, Zenobia Jacobs, Richard G. Roberts and Matthias Meyer

Last updated by author(s): Apr 29, 2021

Reporting Summary

Nature Research wishes to improve the reproducibility of the work that we publish. This form provides structure for consistency and transparency in reporting. For further information on Nature Research policies, see our Editorial Policies and the Editorial Policy Checklist.

Statistics

For all statistical analyses, confirm that the following items are present in the figure legend, table legend, main text, or Methods section.

n/a	Confirmed	
☐	☒	The exact sample size (*n*) for each experimental group/condition, given as a discrete number and unit of measurement
☐	☒	A statement on whether measurements were taken from distinct samples or whether the same sample was measured repeatedly
☐	☒	The statistical test(s) used AND whether they are one- or two-sided<br>*Only common tests should be described solely by name; describe more complex techniques in the Methods section.*
☐	☒	A description of all covariates tested
☐	☒	A description of any assumptions or corrections, such as tests of normality and adjustment for multiple comparisons
☐	☒	A full description of the statistical parameters including central tendency (e.g. means) or other basic estimates (e.g. regression coefficient) AND variation (e.g. standard deviation) or associated estimates of uncertainty (e.g. confidence intervals)
☐	☒	For null hypothesis testing, the test statistic (e.g. *F*, *t*, *r*) with confidence intervals, effect sizes, degrees of freedom and *P* value noted<br>*Give P values as exact values whenever suitable.*
☐	☒	For Bayesian analysis, information on the choice of priors and Markov chain Monte Carlo settings
☒	☐	For hierarchical and complex designs, identification of the appropriate level for tests and full reporting of outcomes
☒	☐	Estimates of effect sizes (e.g. Cohen's *d*, Pearson's *r*), indicating how they were calculated

Our web collection on statistics for biologists contains articles on many of the points above.

Software and code

Policy information about availability of computer code

Data collection	No software was used for the collection of data.
Data analysis	Data was analysed using the leehom package that is available at https://bioinf.eva.mpg.de/, bam-rmdup package that is available at https://github.com/mpieva/biohazard-tools, MEGAN, BLAST, kallisto and R version 3.5.1.

For manuscripts utilizing custom algorithms or software that are central to the research but not yet described in published literature, software must be made available to editors and reviewers. We strongly encourage code deposition in a community repository (e.g. GitHub). See the Nature Research guidelines for submitting code & software for further information.

Data

Policy information about availability of data

All manuscripts must include a data availability statement. This statement should provide the following information, where applicable:

- Accession codes, unique identifiers, or web links for publicly available datasets
- A list of figures that have associated raw data
- A description of any restrictions on data availability

The mitochondrial consensus sequences reported from Main Chamber, layer 19 (M65), layer 20 (M71), East Chamber layer 11.4 (E202) and layer 11.4/12.1 (E213) are available in Dryad digital repository(doi:10.5061/dryad.k3j9kd567) and the raw data for each all mammalian mtDNA and human mtDNA enriched libraries are available in the European Nucleotide Archive under accession number PRJEB44036.

Field-specific reporting

Please select the one below that is the best fit for your research. If you are not sure, read the appropriate sections before making your selection.

☒ Life sciences ☐ Behavioural & social sciences ☐ Ecological, evolutionary & environmental sciences

For a reference copy of the document with all sections, see nature.com/documents/nr-reporting-summary-flat.pdf

Life sciences study design

All studies must disclose on these points even when the disclosure is negative.

Sample size	No sample size was determined in advance. The total number of samples was the result of a grid-like sampling scheme comprising at least three columns (when possible) designed to cover the Pleistocene layers from all the three chambers in Denisova Cave. The resulting number of 728 samples was determined to be sufficient as each Pleistocene layer in each chamber was sampled at least twice.
Data exclusions	No samples were excluded from the study.
Replication	The experiment of recovering hominin mtDNA from sediment was replicated from 80 samples by generating two libraries from the same sub-sample. The recovery of hominin mtDNA was also tested by taking varying numbers of independent sediment subsamples (8, 6 and 4) from different sediment samples (5, 10, and 49 respectively).The results of these experiments are summarized in Extended Data Figure 6 and described at length in Supplementary Section 4.
Randomization	No randomization was performed as this was not relevant for our study. All samples were evaluated for the presence of ancient faunal and hominin mitochondrial DNA and analysis continued for those that contained ancient DNA.
Blinding	Blinding was not relevant for data collection as samples were selected based on their location within the stratigraphy. Blinding was also not relevant for downstream analysis as previously established analysis pipelines was used for the processing of the data and results were interpreted based on expectations from and comparisons to previously published ancient and modern mitochondrial genomes.

Reporting for specific materials, systems and methods

We require information from authors about some types of materials, experimental systems and methods used in many studies. Here, indicate whether each material, system or method listed is relevant to your study. If you are not sure if a list item applies to your research, read the appropriate section before selecting a response.

Materials & experimental systems

n/a	Involved in the study	
☒	☐	Antibodies
☒	☐	Eukaryotic cell lines
☐	☒	Palaeontology and archaeology
☒	☐	Animals and other organisms
☒	☐	Human research participants
☒	☐	Clinical data
☒	☐	Dual use research of concern

Methods

n/a	Involved in the study	
☒	☐	ChIP-seq
☒	☐	Flow cytometry
☒	☐	MRI-based neuroimaging

Palaeontology and Archaeology

Specimen provenance	Materials were acquired as part of an agreement of scientific cooperation between the Institute of Archaeology and Ethnography, Siberian Branch of the Russian Academy of Sciences and the Max Planck Institute for Evolutionary Anthropology for projects in the field of palaeogenetics in North Asia, signed on December 25, 2018 and valid for a duration of five years. The Institute of Archaeology and Ethnography, Siberian Branch of the Russian Academy of Science oversees the excavation of Denisova Cave and obtained all permits necessary for conducting archaeological fieldwork and research associated with this project from the Ministry of Culture of the Russian Federation.
Specimen deposition	All sediment samples remain stored at the Max Planck Institute for Evolutionary Anthropology in Leipzig, Germany
Dating methods	No new dates are provided in this study. All ages referred to in this paper are from Jacobs et al. (Nature 565, 594–599, 2019) and Douka et al. (Nature 565, 640–644, 2019).

☐ Tick this box to confirm that the raw and calibrated dates are available in the paper or in Supplementary Information.

Ethics oversight	All necessary permits for excavations at Denisova Cave were obtained by the Institute of Archaeology and Ethnography, Siberian Branch of the Russian Academy of Science from the Ministry of Culture of the Russian Federation.

Note that full information on the approval of the study protocol must also be provided in the manuscript.
